# An α‐Helix‐Mimicking 12,13‐Helix: Designed α/β/γ‐Foldamers as Selective Inhibitors of Protein–Protein Interactions

**DOI:** 10.1002/anie.201604517

**Published:** 2016-07-28

**Authors:** Claire M. Grison, Jennifer A. Miles, Sylvie Robin, Andrew J. Wilson, David J. Aitken

**Affiliations:** ^1^ CP3A Organic Synthesis Group, ICMMO, CNRSUniversité Paris Sud, Université Paris Saclay15 Rue George Clemenceau91405Orsay CedexFrance; ^2^ School of ChemistryUniversity of LeedsWoodhouse LaneLeedsLS2 9JTUK; ^3^ Astbury Centre for Structural Molecular BiologyUniversity of LeedsWoodhouse LaneLeedsLS2 9JTUK; ^4^ UFR Sciences Pharmaceutiques et BiologiquesUniversité Paris Descartes4 Avenue de l'Observatoire75270Paris cedex 06France

**Keywords:** α-helix mimetics, foldamers, inhibitors, peptidomimetics, protein–protein interactions

## Abstract

A major current challenge in bioorganic chemistry is the identification of effective mimics of protein secondary structures that act as inhibitors of protein–protein interactions (PPIs). In this work, *trans*‐2‐aminocyclobutanecarboxylic acid (*t*ACBC) was used as the key β‐amino acid component in the design of α/β/γ**‐**peptides to structurally mimic a native α‐helix. Suitably functionalized α/β/γ‐peptides assume an α‐helix‐mimicking 12,13‐helix conformation in solution, exhibit enhanced proteolytic stability in comparison to the wild‐type α‐peptide parent sequence from which they are derived, and act as selective inhibitors of the p53/*h*DM2 interaction.

Foldamers are unnatural oligomers that adopt well‐defined secondary and tertiary conformations.^1^, ^2^, ^3^, ^4^ As bioinspired structures, some of them have been validated as useful reagents to modulate (therapeutically important) biological processes and systems,^4^, ^5^, ^6^, ^7^, ^8^, ^9^ and others as building blocks for use in synthetic biology^10^, ^11^, ^12^, ^13^, ^14^ or the construction of functional materials.^15^, ^16^ A particularly fertile area is centred on the search for foldamers that mimic natural secondary structures (specifically α‐helices) and thereby act as inhibitors of protein–protein interactions (PPIs).^17^, ^18^, ^19^, ^20^, ^21^ However, there is still a need to develop ligands that more effectively mimic the conformation and molecular recognition capabilities of the α‐helix. Herein, we present the bottom‐up design of hybrid α/β/γ‐peptides that assume an α‐helix‐mimicking 12,13‐helical conformation and function as effective inhibitors of the p53/*h*DM2 interaction.

Amongst a multitude of foldamer classes where structural/ conformational determinants have been mapped,^1^, ^2^, ^3^, ^4^ β‐peptides and hybrid α/β‐peptides, in which β‐amino acids are dispersed along an α‐peptide backbone, can inhibit α‐helix‐mediated protein–protein interactions^22^, ^23^, ^24^, ^25^, ^26^, ^27^, ^28^, ^29^ and mimic the structure and the function of protein surfaces.^30^, ^31^ Nonetheless foldamers that more accurately mimic the topology and topography of the α‐helix might prove advantageous in comparison to β‐ and α/β‐peptides, which may not fully mimic the spatial presentation of α‐helix side chains. Several foldamer scaffolds have been hypothesized to have potential for the inhibition of α‐helix‐mediated PPIs,^32^, ^33^, ^34^, ^35^ but they have not yet been shown to do so experimentally. β/γ‐Peptide sequences fall into this category: a dipeptide of β‐ and γ‐residues forming a 13‐membered hydrogen‐bonded ring (C=O(*i*)‐NH(*i*+3)) is analogous to a tripeptide of α‐amino acids forming the 13‐membered hydrogen‐bonded ring (C=O(*i*)‐NH(*i*+4)) of the native α‐helix. The 13‐helix represents a more accurate topographical mimic of the natural α(4^13^)‐helix and represents an attractive template on which to elaborate inhibitors of protein–protein interactions. Whilst both the Gellman and Balaram groups have previously demonstrated that the introduction of β and γ residues is tolerated within sequences of α‐amino acids, which retain the secondary structure of the α‐helix,^36^, ^37^, ^38^ the approach described herein is quite distinct in that a novel‐fold is designed in a bottom‐up manner to mimic the topology and side‐chain presentation of an α‐helix.

We have recently demonstrated that an alternating sequence of the β‐amino acid *trans*‐2‐aminocyclobutanecarboxylic acid (*t*ACBC) and γ‐amino acids can adopt a 9/8‐ribbon^39^ or a robust 13‐helix^40^ in solution, depending on the absence or presence of branching within the γ‐amino acid monomer. We therefore examined the ability of β/γ‐peptide manifolds to behave as α‐helix mimetics by designing mimetics of the N‐terminal helical domain (residues 19–26) of the transcription factor p53 (Figure 1). β/γ‐Peptide mimetics were designed to display three known hot‐spot residues of p53 at the correct positions: Phe (*i*), Trp (*i*+4), and Leu (*i*+7). The primary sequence of p53_19–26_ and a β/γ‐peptide backbone with alternating *t*ACBC and γ‐amino acids were aligned in order to map appropriately positioned side chains. Although Ser20 from p53 appears to align well with the γ‐residue at position 2 in the α/β/γ‐peptides, it is not a hot‐spot residue and hence for this first generation, γ^4^‐Ala was used in this position to ensure that a helical conformation would be promoted.^40^ Two series of four α/β/γ‐hexapeptides (**1**–**8**; Figure 1) were proposed: *N*‐Boc‐protected (**1**–**4**) and *N*‐acetamide (**5**–**8**) peptides. Both series featured γ^4^‐Leu and Leu at the C terminus, since only the amino acid side chain was a prerequisite, as well as γ^4^‐Trp and γ^4^‐Phe, since previous studies have shown that the replacement of indole with phenyl in the Trp21‐mimicking position does not necessarily alter the affinity of peptidomimetics for hDM2.^18^ Peptides **1**–**8** were prepared by using standard solution‐state methods (see the Supporting Information), and conformational analysis was performed by using solution‐state spectroscopic techniques and molecular modelling.

**Figure 1:**
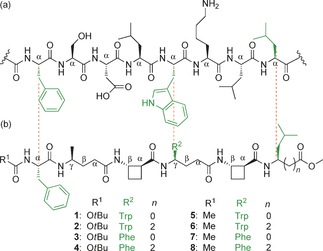
Alignment of key side chains. a) The p53(19—26) segment. b) Structures of the α/β/γ‐peptide helix mimetics (**1**–**8**) studied in this work.

The ^1^H NMR spectra of the *N*‐Boc‐protected peptides **1**–**4** in CDCl_3_ were well‐defined and the signals were conveniently dispersed, thus allowing complete residue assignment and unambiguous attribution of all signals pertinent for conformational analysis by using standard 1D and 2D NMR sequences. ROESY experiments revealed correlations between non‐adjacent residues (Figure 2 a); similar ROE correlation patterns were observed for all four *N*‐Boc‐protected α/β/γ‐peptides. In β/γ‐segments, ROEs characteristic of 13‐membered hydrogen‐bonded rings were detected: Hβ(*i*)‐NH(*i*+2), Hβ(*i*)‐Hα(*i*+2), Hγ(*i*)‐NH(*i*+2).^40^ In N‐terminal α/γ/β‐segments, related ROEs were detected: Hα(*i*)‐NH(*i*+2), Hα(*i*)‐Hα(*i*+2).^40^ These latter correlations are indicative of a 12‐membered hydrogen‐bonded ring in this segment of the peptide, which was corroborated by the down‐field chemical shift values and the high [D_6_]DMSO titration coefficients observed for the amide NH signals from Phe1 and γ^4^‐Ala2 in the ^1^H NMR spectra (see the Supporting Information for more details). These data fully support the proposal that the α/β/γ‐peptides **1**–**4** predominantly adopt a well‐defined folded conformation containing one C12 and three C13 features (Figure 2 b), which we refer to as a 12,13‐helix.

**Figure 2:**
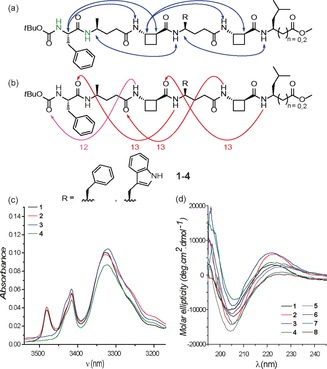
Conformational analyses of peptides **1**–**8**. a) Structures of *N*‐Boc‐protected α/β/γ‐peptides **1**–**4**, highlighting significant ^1^H NMR data (10 mm in CDCl_3_): amide NH signals with high [D_6_]DMSO titration coefficients (in green) and ROEs observed between non‐adjacent residues (blue arrows). b) The same structures of **1**–**4** showing the interpretation of the above data in terms of a 12,13‐helix, comprised of a succession of 12‐ and 13‐membered hydrogen‐bonded rings (pink and red, respectively). c) IR absorption spectra (10 mm in CDCl_3_) of *N*‐Boc‐protected α/β/γ‐peptides **1**–**4**. d) CD spectra (0.2 mm in MeOH) of α/β/γ‐peptides **1**–**8**.

Solution‐state IR absorption spectra of the *N*‐Boc‐protected peptides **1**–**4** recorded in CDCl_3_ (Figure 2 c) further support these conclusions. In all cases, in addition to a free NH absorption (around 3425 cm^−1^), a strong, low‐frequency amide NH absorption band was observed (around 3325 cm^−1^), as would be expected for 12‐ and 13‐membered H‐bonded features. A free indole NH absorption band was also observed (around 3480 cm^−1^) for peptides **1** and **2**.

The lower solubility of peptides **5**–**8** in aprotic solvents (<1 mm in CDCl_3_) precluded similar NMR and IR studies for these compounds. However, the far‐UV CD spectra of all α/β/γ‐peptides **1**–**8** were recorded in 0.2 mm MeOH solution and each showed a marked Cotton effect, presenting a minimum around 206 nm and a maximum around 224 nm (Figure 2 d). These data compare closely with the methanol‐solution signatures of both the 13‐helix adopted by β/γ‐peptides and the 12‐helix adopted by β‐peptides,^40^, ^41^, ^42^ thus suggesting that α/β/γ‐peptides **1**–**8** adopt a similar folded conformation in the same solvent. Collectively, the NMR, IR, and CD data provide strong evidence that α/β/γ‐peptides **1**–**8** are capable of adopting a hydrogen‐bonded helical conformation in hydrogen‐bonding and non‐hydrogen‐bonding solvents.

A hybrid Monte Carlo multiple minima (MCMM) molecular mechanics conformational search^43^ was carried out on α/β/γ‐peptides using MacroModel 10.6 and the MMFFs force field without restraints; in chloroform, octanol, or water for peptides **1**–**4**, and in octanol or water for peptides **5**–**8**. The conformational landscapes were largely dominated by a well‐defined 12,13‐helix (relative abundance >67 % in chloroform, 100 % in octanol) comprised of the C12 and C13 features as deduced from the spectroscopic analyses. As we anticipated, a solvent with a higher dielectric constant (water) does not significantly change the conformational landscape of the α/β/γ‐peptides; some fraying at the N‐terminus is observed, which reduces the population of 12,13‐ and 13‐helical conformers to the range 47–79 %. However, the key central residues are essentially locked in a 13‐helical conformation (see the Supporting Information). Peptides **1**–**8** were subjected to ab initio geometry optimization by DFT using Gaussian09 at the B3LYP/6‐311G(d,p) level of theory. The lowest‐energy structures of **1**–**4** and **5**–**8** were superimposed (Figure 3 a,b) and the backbone of peptide **2** (as a representative example) was overlaid with the crystal structure of p53_16–29_ (Figure 3 c) using γ^4^‐amino acid C(α) atoms as the basis of the alignment. The superimposition gave an excellent RMSD value of around 0.9 Å (Figure 3 d,e), thus strongly suggesting that the α/β/γ‐peptides **1**–**8** effectively mimic an α‐helix. Gratifyingly, selected side chains can be accommodated in the required positions to mimic those of a native α‐peptide without affecting the ability to adopt a helix, which suggests that α/β/γ‐peptides might have wider use as α‐helix‐mimetic scaffolds.

**Figure 3:**
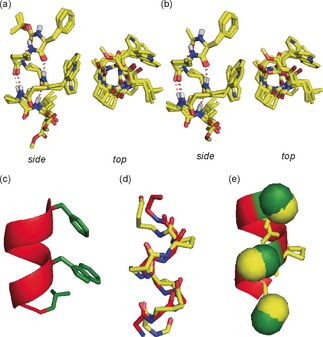
Molecular modelling of α/β/γ‐peptides. a) Superimposition of the calculated lowest‐energy conformers of α/β/γ‐peptides **1—4**. b) The same such structures of peptides **5—8**. C yellow, N blue and O red. c) Representation of the crystal structure of p53_16–29_ excised from its complex with *h*DM2 (PDB ID: 1YCR) highlighting the helical backbone (in red) and the hot‐spot side chains (in green). d) Overlays of the backbones of α/β/γ‐peptide **2** (yellow backbone) and of p53_16–29_ peptide (red backbone); RMSD is 0.89 Å for the α‐carbons. e) Hot‐spot matching. Colors as for (c) and (d).

The proteolytic stability of α/β/γ‐peptides **1**–**8** was investigated using α‐chymotrypsin (α‐CT) as a representative protease, since this enzyme selectively hydrolyses the amide bond on the C‐terminal side of hydrophobic residues such as Leu, Trp, and Phe, all of which are present in the α/β/γ‐peptide sequences. The p53_15–31_ peptide was also studied for comparison (see the Supporting Information). Using HPLC to assess the progress of peptide digestion, it transpired that all eight α/β/γ‐peptides displayed considerably greater resistance to α‐CT than did the native p53_15–31_ (≥32‐fold for **2**, **4**, **6**, and **8** and ≥10‐fold for **1**, **3**, **5**, and **7**; Figure 4 a). For the latter four peptides, the proteolytic activity corresponded to methyl ester hydrolysis of the C‐terminal Leu residue; in all eight cases, therefore, the hexapeptide core remained largely intact for the duration of the experiments.

**Figure 4:**
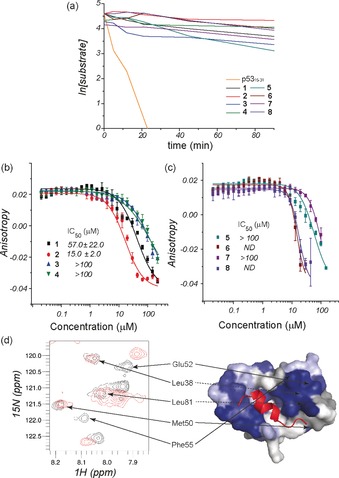
Biophysical analysis of the α/β/γ‐peptides. a) proteolytic degradation of peptides **1**–**8** and p53_15–31_. b, c) Dose–response curves for **1**–**4** (b) and **5**–**8** (c) in an FA competition assay against p53/*h*DM2 (50 nm of FITC–p53 and 150 nm of *h*DM2 in 40 mm phosphate buffer, pH 7.5, 200 mm NaCl, 0.02 mg mL^−1^ BSA, 5 % DMSO). d) ^1^H‐^15^N HSQC spectra of ^15^N‐labelled *h*DM2 (87 μm in 100 mm sodium phosphate buffer, pH 7.3, 2.5 % glycerol, 1 mm DTT, 5 % DMSO), recorded in the absence (black) and the presence (red) of 200 μm **2** (left), with cross‐peaks that move mapped onto the surface of *h*DM2 and shown in blue (right). BSA=bovine serum albumin, DMSO=dimethyl sulfoxide, DTT=1,4‐dithiothreitol.

The ability of the α/β/γ‐peptides to act as functional mimics of p53 was examined by using a fluorescence anisotropy (FA)‐based competition assay wherein the displacement of fluorescein‐labelled p53 (FITC–p53_15–31_) from the binding groove of *h*DM2 is monitored upon titration with the competitor peptide, with the objective of determining a half‐maximal inhibitory concentrating (IC_50_) value (Figure 4 b,c). Peptides **3**, **4**, **5**, and **7** were found to bind to *h*DM2 but were not sufficiently potent ligands to generate full competition curves (IC_50_>100 μm). Peptides **6** and **8** effectively displaced FITC–p53_15–31_ at micromolar concentrations, although the lower asymptote of the competition curve was not achieved for higher concentrations. Peptides **1** and **2** generated full competition curves with good potency: IC_50_ values were calculated as 57±22 μm for **1** and 15±2 μm for **2**. It is noteworthy that the inhibitory potency of **1** and **2** for *h*DM2 is only one order of magnitude lower than that of the native sequence p53_15–31_ (IC_50_=1.2±0.1 μm) and that of nutlin,^44^ a well‐known inhibitor of p53/*h*DM2 (IC_50_=0.434±0.024 μm), and is superior to that previously reported for first‐generation β‐peptides.^27^ The difference in behavior between the peptide pair **1** and **2** and the peptide pair **3** and **4** indicates that γ^4^‐Trp plays a significant role in binding to *h*DM2, as is indeed the case for Trp21 in the native p53 protein. Importantly, in further FA assays conducted for the BODIPY–BAK/Bcl‐x_L_ and the FITC–NOXA B/Mcl‐1 protein–protein interactions, peptides **1** and **2** displayed no inhibitory activity, thus indicating that their binding to *h*DM2 is selective (see the Supporting Information).

Peptide **2** was assessed for its ability to bind to ^15^N‐labelled *h*DM2 at the native p53 protein binding cleft. Peptide **8** was also tested as a negative control for comparison purposes. ^1^H‐^15^N HSQC spectra were recorded in the absence and presence of the designated peptides (Figure 4 d for **2**; see the Supporting Information for **8**). Upon addition of **2**, cross‐peaks in the ^1^H‐^15^N HSQC spectrum shifted throughout the protein, thus indicating a direct interaction with *h*DM2, whereas no significant shifts were observed upon addition of **8**. The chemical shifts were mapped onto the structure of *h*DM2 by using a published NMR assignment.^45^ The shift changes were comparable to those induced by p53_15–29_ peptide in its interaction with *h*DM2.^17^, ^45^, ^46^ Significant diagnostic changes, characterized by strong peak shifts, were observed for the amide NH of Phe55 and His73, which are located at opposite edges of the hydrophobic cleft. This supports the hypothesis that **2** binds to *h*DM2 in the canonical p53 binding site.

In conclusion, α/β/γ‐peptides constructed from *t*ACBC and γ^4^‐amino acids are able to fold into 12,13‐helices to effectively mimic the α‐helix. A demonstrated advantage of the α/β/γ‐peptide motif compared to native α‐peptides is resistance to proteolytic degradation. Moreover, the results of the FA assays establish for the first time that α/β/γ‐peptides can act as functional and selective α‐helix‐mimetic inhibitors of the p53/*h*DM2 interaction. The current design rationale has focused on accurately reproducing the spatial presentation of the three key p53 hot‐spot residues (Phe‐Trp‐Leu); we anticipate that further optimization studies might increase the potency of these promising α/β/γ‐peptide scaffolds through the incorporation of additional side chains other than the key triad and by using affinity‐improvement design features identified in previous work.^47^, ^48^, ^49^ Moreover, subsequent studies will focus on the use of these foldamer manifolds for the programmable bottom‐up design of mimetics of the native α‐helix to target other PPIs.

## Supporting information

As a service to our authors and readers, this journal provides supporting information supplied by the authors. Such materials are peer reviewed and may be re‐organized for online delivery, but are not copy‐edited or typeset. Technical support issues arising from supporting information (other than missing files) should be addressed to the authors.

Supplementary
