# Supplementary material for: An α‐Helix‐Mimicking 12,13‐Helix: Designed α/β/γ‐Foldamers as Selective Inhibitors of Protein–Protein Interactions
Source: Angew Chem Int Ed Engl. 2016 Jul 28;55(37):11096–100. doi: 10.1002/anie.201604517 (PMC5014220; doi:10.1002/anie.201604517)
Supplement: Supplementary file 1 — Supplementary [file ANIE-55-11096-s001.pdf]

Angewandte Chemie
Eine Zeitschrift der Gesellschaft Deutscher Chemiker

Supporting Information

An α-Helix-Mimicking 12,13-Helix: Designed α/β/γ-Foldamers as Selective Inhibitors of Protein–Protein Interactions

Claire M. Grison, Jennifer A. Miles, Sylvie Robin, Andrew J. Wilson, and David J. Aitken**

anie_201604517_sm_miscellaneous_information.pdf

Supporting information

I. Synthesis of peptides **1-8** and **I-VIII** 2

1. General Information 2

2. General procedures 3

3. Synthetic procedures for the preparation of peptides **1-8** and **I-VIII** 4

II. NMR spectroscopic analysis of peptides **1-8** and **I-VIII** 28

1. ^{1}H and ^{13}C NMR spectra 28

2. DMSO-d_6 titrations 44

3. ROESY correlations 49

III. Molecular Modelling 54

1. Hybrid MCMM calculation 54

i. Table of conformations obtained in $CHCl_3$, $C_8H_{17}OH$ and H_2O 54

ii. Side and top views of the superimposed helical conformers (12-13-13-13 and /-13-13-13) of **1-8** in a water medium 56

2. Geometry optimization of the 12,13-helices by DFT 57

IV. Proteolysis studies of peptides **1-8** 58

1. HPLC traces of the positive control (native p53) in presence of α-Chymotrypsin 58

2. HPLC traces of Boc-peptides **1-4** in presence of α-Chymotrypsin 59

3. HPLC traces of Boc-peptides **5-8** in presence of α-Chymotrypsin 60

V. Fluorescence anisotropy competition assays with peptides **1-8** 61

1. Dose response curves of peptides **1-8** against BODIPY-BAK/Bcl-xL 62

2. Dose response curves of peptides **1-8** against FITC-NOXA B/Mcl-1 62

VI. ^{15}N-^{1}H HSQC NMR of peptides **2** and **8** in complex with *h*DM2 63

1. ^{15}N-^{1}H HSQC and changes in chemical shift of hDM2 in presence of peptide **8** 64

2. ^{15}N-^{1}H HSQC and changes in chemical shift of hDM2 in presence of peptide **2** 65

VII. References 66

I. Synthesis of peptides 1-8 and I-VIII

1. General Information

(1*S*,2*S*)-2-(*tert*-butyloxyamino)cyclobutane-1-carboxylic acid (Boc–ACBC–OH) was obtained following the published procedure.[1] Boc–(1*S*,2*S*)-ACBC–OBn was obtained using the procedure described for the (1*R*,2*R*) enantiomer.[2] Dichloromethane was dried over activated alumina, DMF was distilled from CaH_2. All other reagents and solvents were of commercial grade and were used without further purification. The Boc protected γ^4-amino acids were purchased from PolyPeptide. Flash chromatography was performed using Combiflash (Teledyne ISCO) with columns of 15−40 µm silica gel (SI60, Merck Chimie SAS). Analytical thin-layer chromatography was performed with 0.25 mm commercial silica gel plates (EMD, Silica Gel $60F_{254}$). TLC plates were visualized by UV fluorescence at 254 nm then revealed using a ninhydrin solution (14 mM in EtOH); retention factors (R_f) are given for such analyses. Routine nuclear magnetic resonance (NMR) data were acquired on Bruker spectrometers operating at 360, 400 or 600 MHz for ^{1}H and at 90 or 100 MHz for ^{13}C. Chemical shifts (δ) are reported in parts per million from tetramethylsilane. Splitting patterns for ^{1}H NMR signals are designated as: s (singlet), d (doublet), t (triplet), bs (broad singlet) and m (multiplet). Coupling constants (*J*) are reported in hertz. High-resolution mass spectrometry (HRMS) data were recorded using the electrospray ionization technique in positive mode (ESI+) with a MicroTOF-Q (Bruker) analyzer. Fourier-transform infrared absorption spectroscopy (IR) was performed for solutions in $CDCl_3$ (10 mM) retained in a 0.2 mm path length NaCl solution cell with a $CDCl_3$ background; spectra were recorded on a Spectrum One (Perkin-Elmer) spectrometer. Maximum absorbances (ν_{max}) are reported for significant bands in cm^{-1}. Melting points were obtained in open capillary tubes using a Büchi B-545 melting point apparatus. Optical rotations were measured on a Specord 205 instrument (Analytik-Jena) using a 10 cm quartz cell; values for $[\alpha]_D^T$ were obtained with the D-line of sodium at the indicated temperature *T*, using solutions of concentration (c) in units of g·100 mL^{-1}. LC-MS experiments were run on a Waters Micromass ZQ spectrometer, samples ionized by electrospray and analysed by a time-of-flight mass spectrometer, or a Bruker Daltronics HCTUltra™ series spectrometer, samples ionized by electrospray. All experiments were run through a C18 column on an acetonitrile/water gradient. HPLC purification was performed on an Agilent 1200 series instrument equipped with a Phenomenex C18 column (50 × 2 mm) using acetonitrile:water as the eluent for positive ion spectra. Values are reported as a ratio of mass to charge. Nominal mass spectra and accurate (4 d.p.) mass spectra were recorded on a Bruker Daltonics micrOTOF Premier Mass Spectrometer, under positive ESI conditions unless otherwise stated.

2. *General procedures*

General procedure A for the basic hydrolysis of methyl ester protecting group

To a solution of Boc–peptide–OMe (1 eq.) in a 1 : 1 mixture of H_2O : THF (10 mL / mmol) was added $LiOH{\cdot}H_2O$ (1.5 eq.). The white suspension was stirred for 3 h at room temperature. THF was then removed under reduced pressure. The aqueous solution was then slowly acidified at 0 °C with a solution of HCl (1 M) to reach pH 3. Three sucessive extractions with EtOAc were performed. The combined organic layers were dried over Na_2SO_4, filtered and concentrated under reduced pressure to give the corresponding carboxylic acid, Boc–peptide–OH.

General procedure B for the cleavage of *tert*-butoxycarbonyl protecting group

To a solution of Boc–peptide–OMe (1 eq.) in dry CH_2Cl_2 (25 mL / mmol) was added TFA (30 eq.) at room temperature under an argon atmosphere. The resulting yellowish mixture was stirred until the reaction was complete (TLC monitoring). CH_2Cl_2 was then evaporated under reduced pressure. Toluene was added to co-evaporate the excess of TFA. The corresponding TFA salt, $TFA{\cdot}H_2N$–peptide–OMe, was directly engaged to the coupling reaction without further purification.

General procedure C for coupling reaction

To a solution of Boc–peptide–OH (1 eq.) in a mixture of CH_2Cl_2 and DMF (6 mL / mmol) was added DIPEA (2 eq.) followed by HATU (1.05 eq.). The resulting mixture was stirred for 10 min at room temperature and the solution became brownish. A solution of the TFA salt partner (1 eq.) and DIPEA (in sufficient quantity to reach pH 9) in CH_2Cl_2 (6 mL / mmol) was prepared and added to the reaction mixture which was stirred overnight. Solvents were removed under reduced pressure. The crude product was dissolved in EtOAc and successively washed by a saturated solution of $NaHCO_3$, brine, a 1 M solution of HCl and brine. The organic layer was dried over Na_2SO_4, filtered and concentrated under reduced pressure. The crude residue was well dried under vacuum then purified by flash chromatography.

General procedure D for *N*-acetylation

To a solution of the TFA salt, $TFA{\cdot}H_2N$–peptide–OMe, (1 eq.) in CH_2Cl_2 (6 mL / mmol) was added DIPEA (in sufficient quantity to reach pH 9) and anhydride acetic (1.5 eq.). The resulting mixture was stirred overnight at room temperature. The reaction mixture was then successively washed by a 1 M solution of HCl and brine. The organic layer was dried over Na_2SO_4, filtered and concentrated under reduced pressure to afford the corresponding acetamide, Ac–peptide–OMe.

Peptides used in the biophysical assays were further purified by mass-directed preparative HPLC to reach a purity higher than 95%. All purifications were run with an acetonitrile/water gradient (0-95% acetonitrile over 20 minutes).

3. *Synthetic procedures for the preparation of peptides **1-8** and **I-VIII***

Boc–(*R*)-γ⁴-Ala–(1*S*,2*S*)-ACBC–OMe I

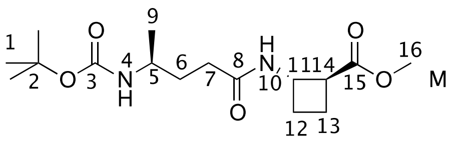

To a solution of Boc–(1*S*,2*S*)-ACBC–OH (150 mg, 0.70 mmol, 1 eq.) in CH_3OH (5 mL) was slowly added an excess of TMSCl (267 μL, 227 mg, 2.1 mmol, 3 eq.) under an argon atmosphere. The reaction mixture was stirred overnight at room temperature. CH_3OH and TMSCl were evaporated under reduced pressure to give a pale yellow solid, HCl·H_2N–(1*S*,2*S*)-ACBC–OMe which was directly used in the coupling reaction. Following the general procedure C, the coupling reaction was performed with Boc–(*R*)-γ^4-Ala–OH (152 mg, 0.70 mmol), DIPEA (245 μL, 180 mg, 1.40 mmol) and HATU (276 mg, 0.70 mmol) in a mixture of CH_2Cl_2 / DMF (3 mL / 1 mL); and HCl·H_2N–(1*S*,2*S*)-ACBC–OMe (105 mg, 0.70 mmol), DIPEA (485 μL, 361 mg, 2.80 mmol) in DMF (2 mL) for 3 d. The purification was carried out by flash chromatography (gradient from 10 / 90 to 80 / 20 : EtOAc / PE) to give **I** as a white sticky solid (166 mg, 72%). R_f 0.53 (CH_3OH / CH_2Cl_2 = 10 / 90); $[a]_D^{25}$= + 16 (c. 0.50 in CH_3OH); ^1H NMR (400MHz, $CDCl_3$) δ 1.10 (d, J = 6.4 Hz, 3H, 3H-9), 1.40 (s, 9H, 9H-1), 1.68 (bs, 2H, 2H-6), 1.86-2.02 (m, 3H, 2H-13, H-12), 2.14-2.22 (m, 3H, 2H-7, H-12'), 3.03.09 (m, 1H, H-14), 3.64 (s, 4H, H-5, 3H-16), 4.47-4.55 (m, 1H, H-11), 4.59 (d, J = 8.4 Hz, 1H, H-4), 7.09 (d, J = 4.9 Hz, 1H, H-10); ^{13}C NMR (100 MHz, $CDCl_3$) δ 18.8 (C-13), 21.0 (C-9), 26.4 (C-12), 28.3 (C-1), 32.8 (C-7), 33.1 (C-6), 45.8 (C-5), 46.5 (C-14), 47.0 (C-11), 51.8 (C-16), 79.4 (C-2), 156.1 (C-3), 172.8 (C-8), 174.2 (C-15); IR ν_{max} 1438, 1453, 1508, 1669, 1695, 1727, 2874, 2955, 2981, 3307 (br), 3439 cm^{-1}; HRMS (ESI): $[M+Na]^+$, 351.1908 found, $C_{16}H_{28}N_2NaO_5$ 351.1890 calcd.

Boc–Phe–(*R*)-γ⁴-Ala–(1*S*,2*S*)-ACBC–OMe II

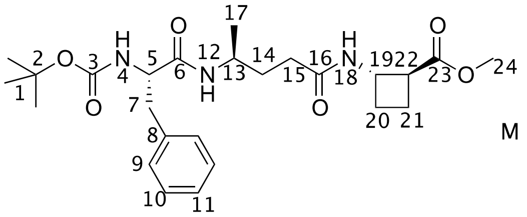

Following the general procedure B, **I** (94 mg, 0.29 mmol) was deprotected in 1.2 h to give the corresponding TFA salt, TFA·H_2N–γ^4-Ala–(1*S*,2*S*)-ACBC–OMe.

To a solution of Boc–Phe–OH (77 mg, 0.29 mmol, 1 eq.) in CH_2Cl_2 (1.5 mL) were added HOAt (39 mg, 0.29 mmol, 1 eq.) followed by EDCI·HCl (61 mg, 0.32 mmol, 1.1 eq.) at 0 °C under inert atmosphere. After 15 min of activation, a solution of TFA·H_2N–γ^4-Ala–(1*S*,2*S*)-ACBC–OMe (98 mg, 0.29 mmol) and DIPEA (182 μL, 138 mg, 1.16 mmol, 4 eq.) in a mixture of CH_2Cl_2 / DMF (2 / 0.5 mL) was added to the reaction mixture. The reaction was stirred overnight at room temperature. After such time, the reaction mixture was washed with a saturated solution of $NaHCO_3$, HCl (1 M) and H_2O, dried over Na_2SO_4 and filtered. Solvents were evaporated under reduced pressure. The crude residue was purified by flash chromatography (gradient from 10 / 90 to 60 / 40 : EtOAc / PE) to give **II** (80 mg, 55%). R_f 0.59 (CH_3OH / CH_2Cl_2 = 10 / 90); ^1H NMR (250 MHz, $CDCl_3$) δ 1.08 (d, *J* = 6.4 Hz, 3H, 3H-17), 1.42 (s, 9H, 9H-1), 1.50-1.58 (m, 2H, 2H-14), 1.68-2.03 (m, 5H, H-15, H-15', H-20, 2H-21), 2.18-2.32 (m, 1H, H-20'), 3.06 (d, *J* = 6.8 Hz, 2H, 2H-7), 2.11-2.18 (m, 1H, H-22), 3.67 (s, 3H, 3H-24), 3.86 (bs, 1H, H-13), 4.29 (td, *J* = 7.1 Hz, *J* = 6.8 Hz, 1H, H-5), 4.44-4.57 (m, 1H, H-19), 5.14 (d, *J* = 7.1 Hz, 1H, H-4), 6.12 (d, *J* = 6.5 Hz, 1H, H-12), 7.11 (bs, 1H, H-18), 7.21-7.34 (m, 5H, 2H-9, 2H-10, H-11); ^{13}C NMR (62.5 MHz, $CDCl_3$) δ 18.4 (C-21), 21.0 (C-17), 27.0 (C-20), 28.3 (C-1), 32.8 , 32.9 (C-14, C-15), 38.2 (C-7), 44.8 (C-13), 46.6 (C-22), 47.6 (C-19), 51.8 (C-24), 56.3 (C-5), 80.6 (C-2), 127.1, 128.7, 129.3 (C-9, C-10, C-11), 136.5 (C-8), 155.6 (C-3), 171.2, 172.0 (C-6, C-16), 173.5 (C-23); IR ν_{max} 1495, 1522, 1669, 1715, 1723, 2878, 2951, 2982, 3069, 3304, 3353 (br), 3424 cm^{-1}; HRMS (ESI): $[M+Na]^+$, 498.2594 found, $C_{25}H_{37}N_3NaO_6$ 498.2575 calcd.

Boc–(*R*)-γ⁴-Trp–(1*S*,2*S*)-ACBC–OMe III

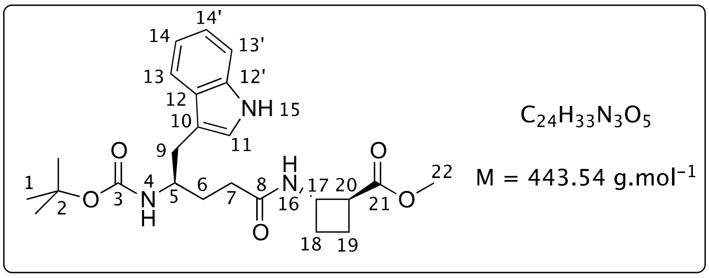

To a solution of Boc–(1*S*,2*S*)-ACBC–OH (150 mg, 0.70 mmol, 1 eq.) in CH_3OH (5 mL) was slowly added an excess of TMSCl (267 μL, 227 mg, 2.1 mmol, 3 eq.) under an argonatmosphere. The reaction mixture was stirred overnight at room temperature. CH_3OH and TMSCl were evaporated under reduced pressure to give a pale yellow solid, HCl·H_2N–(1S,2S)-ACBC–OMe which was directly used in the coupling reaction. Following the general procedure C, the coupling reaction was performed with Boc–(*R*)-γ^4-Trp–OH (156 mg, 0.47 mmol), DIPEA (165 μL, 121 mg, 0.94 mmol) and HATU (185 mg, 0.49 mmol) in a mixture of CH_2Cl_2 / DMF (1 mL / 1 mL); and HCl·H_2N–(1S,2S)-ACBC–OMe (105 mg, 0.70 mmol), DIPEA (165 μL, 121 mg, 0.94 mmol) in DMF (2 mL) overnight. The purification was carried out by flash chromatography (gradient from 10 / 90 to 80 / 20 : EtOAc / PE) to give **III** as a white sticky solid (170 mg, 81%); R_f 0.55 (CH_3OH / CH_2Cl_2 = 10 / 90); $[\alpha]_D^{26}$= + 14 (c. 0.50 in CH_3OH); ^{1}H NMR (360 MHz, $CDCl_3$) δ 1.43 (s, 9H, 9H-1), 1.62 (bs, 2H, 2H-6), 1.86-2.20 (m, 6H, 2H-7, 2H-18, 2H-19), 2.89 (d, J = 6.6 Hz, 2H, 2H-9), 3.00-3.04 (m, 1H, H-20), 3.62 (s, 3H, 3H-22), 3.92 (bs, 1H, H-5), 4.45-4.54 (m, 1H, H-17), 4.72 (d, J = 9.6 Hz, 1H, H-4), 6.88 (d, J = 7.1 Hz, 1H, H-16), 6.96 (s, 1H, H-11), 7.10 (t, J = 7.7 Hz, 1H, H-14), 7.17 (t, J = 7.2 Hz, 1H, H-14’), 7.35 (d, J = 7.8 Hz, 1H, H-13), 7.59 (d, J = 7.8 Hz, 1H, H-13’), 8.67 (bs, 1H, H-15); ^{13}C NMR (90 MHz, $CDCl_3$) δ 18.8 (C-7), 26.5 (C-18), 28.4 (C-1), 30.7, 31.1 (C-6, C-9), 33.1 (C-19), 46.6 (C-20), 47.2 (C-17), 50.4 (C-5), 51.9 (C-22), 79.5 (C-2), 111.2 (C-12), 111.3 (C-13), 118.8 (C-13’), 119.3 (C-14), 121.8 (C-14’), 123.0 (C-11), 127.8, 136.3 (C-10, C-12’), 156.5 (C-3), 172.9 (C-8), 174.0 (C-21); IR ν_{max} 1439, 1457, 1505, 1669, 1696, 1725, 2869, 2954, 2981, 3058, 3310 (br), 3436, 3480 cm^{-1}; HRMS (ESI): $[M+Na]^+$, 466.2309 found, $C_{24}H_{33}N_3NaO_5$ 466.2312 calcd.

Boc–(*R*)-γ⁴-Trp–(1*S*,2*S*)-ACBC–Leu–OMe IV

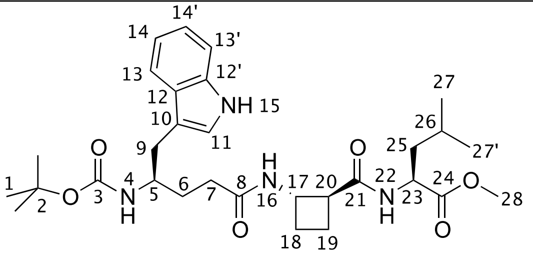

Following the general procedure A, methyl ester of **III** (193 mg, 0.44 mmol) was hydrolysed to give the corresponding carboxylic acid, Boc–(*R*)-γ⁴-Trp–(1*S*,2*S*)-ACBC–OH (176 mg, 94 % crude yield). Following the general procedure C, the coupling reaction was performed with Boc–(*R*)-γ⁴-Trp–(1*S*,2*S*)-ACBC–OH (176 mg, 0.41 mmol), DIPEA (144 μL, 107 mg, 0.83 mmol) and HATU (161 mg, 0.43 mmol) in a mixture of CH_2Cl_2 / DMF (2 mL / 1 mL), and H_2N–Leu–OMe (98 mg, 0.50 mmol), DIPEA (85 μL, 63 mg, 0.49 mmol) overnight. The purification was carried out by flash chromatography (gradient from 10 / 90 to 100 / 0 : EtOAc / PE) to give **IV** as a white foam (123 mg, 54%). R_f 0.53 (CH_3OH / CH_2Cl_2 = 10 / 90); $[a]_D^{25}$= + 5 (c. 0.50 in CH_3OH); ^1H NMR (250 MHz, $CDCl_3$) δ 0.90 (d, *J* = 5.7 Hz, 3H, 3H-27), 0.93 (d, *J* = 6.1 Hz, 3H, H-27'), 1.45 (s, 9H, 9H-1), 1.62-1.65 (m, 4H, 2H-6, H-25, H-26), 1.82-2.08 (m, 5H, H-7, H-18, 2H-19, H-25'), 2.13-2.27 (m, 2H, H-7', H-18'), 2.89-2.96 (m, 3H, 2H-9, H-20), 3.71 (s, 3H, 3H-28), 3.95 (bs, 1H, H-5), 4.35-4.41 (m, 1H, H-17), 4.47-4.55 (m, 1H, H-23), 4.78 (d, *J* = 8.7 Hz, 1H, H-4), 6.99 (s, 1H, H-11), 7.07-7.24 (m, 3H, H-14, H-14', H-16), 7.36 (d, *J* = 8.6 Hz, 1H, H-13), 7.57 (d, *J* = 7.9 Hz, 1H, H-13'), 8.72 (d, *J* = 6.5 Hz, H-22), 8.88 (s, 1H, H-15); ^{13}C NMR (62.5 MHz, $CDCl_3$) δ 18.4 (C-19), 21.8, 22.9 (C-27, C-27'), 24.4 (C-18), 24.9 (C-26), 28.4 (C-1), 31.1, 31.3 (C-9, C-25), 32.9 (C-7), 40.5 (C-6), 48.0 (C-17), 49.2 (C-20), 50.2 (C-5), 51.0 (C-23), 52.2 (C-28), 79.7 (C-2), 111.0 (C-12), 111.4 (C-13), 118.7 (C-13'), 119.3 (C-14), 121.9 (C-14'), 123.0 (C-11), 127.8, 136.4 (C-10, C-12'), 156.6 (C-3), 173.5, 173.8, 173.9 (C-8, C-21, C-24); IR ν_{max} 1440, 1456, 1506, 1552, 1657, 1692, 1741, 2873, 2932, 2959, 3208, 3267 (br), 3435, 3479 cm^{-1}; HRMS (ESI): $[M+Na]^+$, 579.3154 found, $C_{30}H_{44}N_4NaO_6$ 579.3153 calcd.

Boc–(*R*)-γ⁴-Trp–(1*S*,2*S*)-ACBC–(*R*)-γ⁴-Leu–OMe V

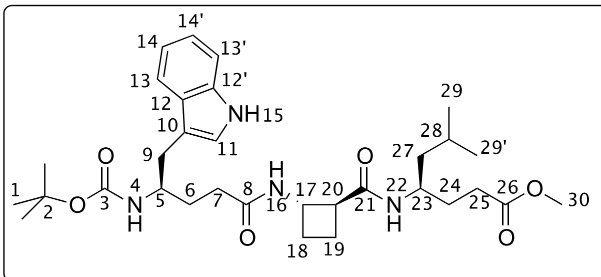

To a solution of Boc–(*R*)-γ^4-Leu–OH (197 mg, 0.70 mmol, 1 eq.) in CH_3OH (5 mL) was slowly added an excess of TMSCl (267 μL, 227 mg, 2.1 mmol, 3 eq.) under an argonatmosphere. The reaction mixture was stirred overnight at room temperature. CH_3OH and TMSCl were evaporated under reduced pressure to give a pale yellow solid, HCl·H_2N–(*R*)-γ^4-Leu–OMe which was directly used in the coupling reaction. Following the general procedure A, methyl ester of **III** (144 mg, 0.33 mmol) was hydrolysed to give the corresponding carboxylic acid, Boc–(*R*)-γ^4-Trp–(1*S*,2*S*)-ACBC–OH (142 mg, 100% crude yield). Following the general procedure C, the coupling reaction was performed with Boc–(*R*)-γ^4-Trp–(1*S*,2*S*)-ACBC–OH (142 mg, 0.33 mmol), DIPEA (115 μL, 85 mg, 0.66 mmol) and HATU (130 mg, 0.35 mmol) in a mixture of CH_2Cl_2 / DMF (2 mL / 1 mL), and HCl·H_2N–(*R*)-γ^4-Leu–OMe (115 mg, 0.50 mmol), DIPEA (170 μL, 127 mg, 0.99 mmol) in DMF (2 mL) overnight. The purification was carried out by flash chromatography (gradient from 10 / 90 to 100 / 0 : EtOAc / PE) to give **V** as a white foam (150 mg, 78%). R_f 0.50 (CH_3OH / CH_2Cl_2 = 10 / 90); $[\alpha]_D^{26}$= + 5 (c. 0.50 in CH_3OH); ^1H NMR (600 MHz, $CDCl_3$) δ 0.85 (d, *J* = 6.4 Hz, 3H, 3H-29), 0.87 (d, *J* = 6.7 Hz, 3H, 3H-29'), 1.19-1.23 (m, 1H, H-27), 1.33-1.39 (m, 1H, H-27'), 1.43 (s, 9H, 9H-1), 1.51-1.55 (m, 1H, H-28), 1.58-1.62 (m, 2H, 2H-6), 1.68-1.72 (m, 1H, H-24), 1.84-1.96 (m, 3H, H-18, H-19, H-24'), 2.01-2.06 (m, 1H, H-19'), 2.12-2.17 (m, 2H, H-7, H-18'), 2.20-2.24 (m, 1H, H-7'), 2.37-2.40 (m, 2H, 2H-25), 2.81-2.86 (m, 1H, H-20), 2.91-2.92 (m, 2H, 2H-9), 3.65 (s, 3H, 3H-30), 3.92-3.97 (m, 2H, H-5, H-23), 4.28-4.33 (m, 1H, H-17), 4.69 (d, *J* = 9.2 Hz, 1H, H-4), 7.02 (s, 1H, H-11), 7.08 (d, *J* = 7.4 Hz, 1H, H-16), 7.11, 7.18 (2t, *J* = 6.9 Hz, *J* = 8.0 Hz, 2H, H-14, H-14'), 7.37, 7.57 (2d, *J* = 8.5 Hz, *J* = 7.57 Hz, 2H, H-13, H-13'), 7.72 (d, *J* = 8.7 Hz, 1H, H-22), 8.62 (s, 1H, H-15); ^{13}C NMR (62.5 MHz, $CDCl_3$) δ 18.6 (C-19), 22.2, 23.1 (C-29, C-29'), 24.5 (C-6), 25.0 (C-28), 28.4 (C-1), 30.8, 30.9, 31.0 (C-9, C-18, C-24, C-25), 32.8 (C-7), 43.9 (C-27), 46.6 (C-23), 47.6 (C-17), 49.7 (C-20), 50.1 (C-5), 51.7 (C-30), 79.6 (C-2), 111.0 (C-10), 111.4, 118.7 (C-13, C-13'), 119.3, 121.8 (C-14, C-14'), 123.1 (C-11), 127.8, 136.4 (C-12, C-12'), 156.5 (C-3), 173.1, 173.9, 174.7 (C-8, C-21, C-26); IR ν_{max} 1439, 1456, 1505, 1556, 1649, 1693, 1729, 2868, 2937, 2957, 3065, 3268 (br), 3434, 3480 cm^{-1}; HRMS (ESI): $[M+Na]^+$, 607.3486 found, $C_{32}H_{48}N_4NaO_6$ 607.3466 calcd.

Boc–Phe–(*R*)-γ^4-Ala–(1*S*,2*S*)-ACBC–(*R*)-γ^4-Trp–(1*S*,2*S*)-ACBC–Leu–OMe 1

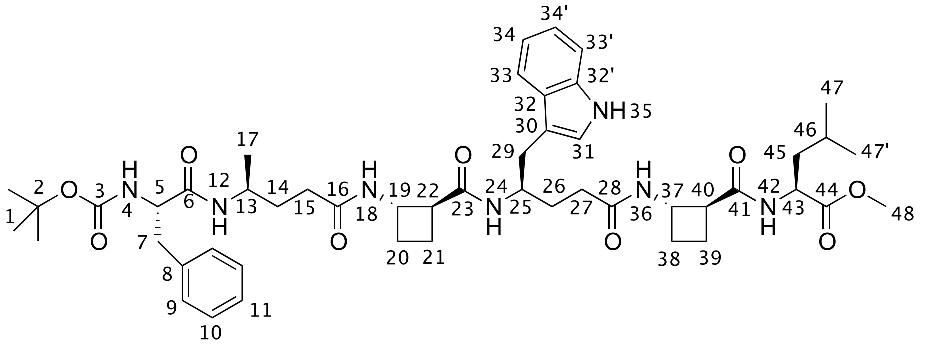

$C_{49}H_{69}N_7O_9$

M = 900.11 g.mol^{-1}

Following the general procedure A, methyl ester of **II** (109 mg, 0.23 mmol) was hydrolysed to give the corresponding carboxylic acid, Boc–Phe–(*R*)-γ^4-Ala–(1*S*,2*S*)-ACBC–OH (100 mg, 91% crude yield). Following the general procedure B, **IV** (115 mg, 0.21 mmol) was deprotected in 2.5 h to give the corresponding TFA salt, TFA·H_2N–(*R*)-γ^4-Trp–(1*S*,2*S*)-ACBC–Leu–OMe. Following the general procedure C, the coupling reaction was performed with Boc–Phe–(*R*)-γ^4-Ala–(1*S*,2*S*)-ACBC–OH (100 mg, 0.21 mmol), DIPEA (73 µL, 54 mg, 0.42 mmol) and HATU (83 mg, 0.22 mmol) in a mixture of CH_2Cl_2 / DMF (2 mL / 1 mL), and TFA·H_2N–(*R*)-γ^4-Trp–(1*S*,2*S*)-ACBC–Leu–OMe (118 mg, 0.21 mmol), DIPEA (145 µL, 108 mg, 0.84 mmol) in DMF (2 mL) overnight. The purification was carried out by flash chromatography (gradient from 10 / 90 to 100 / 0 : EtOAc / PE then from 0 / 100 to 20 / 80 : CH_3OH / CH_2Cl_2) to give **1** as an orange sticky solid (122 mg, 68%). The compound was further purified using mass-directed preparative HPLC (gradient from 5/95 to 95/5 of 0.1% formic acid water/methanol) to afford **1** with purity higher than 96%. R_f 0.47 (CH_3OH / CH_2Cl_2 = 10 / 90); $[\alpha]_D^{26}$= + 24 (c. 0.50 in CH_3OH); ^{1}H NMR (600 MHz, $CDCl_3$) δ 0.87 (d, *J* = 6.4 Hz, 3H, 3H-47), 0.88 (d, *J* = 6.5 Hz, 3H, 3H-47'), 1.03 (d, *J* = 6.2 Hz, 3H, 3H-17), 1.37-1.45 (m, 2H, 2H-14), 1.46 (s, 9H, 9H-1), 1.49-1.70 (m, 7H, 2H-26, H-21, H-39, 2H-45, H-46), 1.86-1.92 (m, 2H, H-15, H-20), 1.99-2.17 (m, 6H, H-15', H-20', H-21', 2H-38, H-39'), 2.21-2.26 (m, 2H, 2H-27), 2.74-2.79 (m, 1H, H-22), 2.93 (dd, *J* = 5.6 Hz, *J* = 5.4 Hz, 2H, 2H-29), 3.06-3.13 (m, 3H, 2H-7, H-40), 3.71 (s, 3H, 3H-48), 3.85 (bs, 1H, H-13), 4.24-4.27 (m, 1H, H-5), 4.54-4.39 (m, 1H, H-25), 4.42-4.53 (m, 3H, H-19, H-37, H-43), 5.15 (d, *J* = 4.1 Hz, 1H, H-4), 6.02 (d, *J* = 10.4 Hz, 1H, H-12), 7.05-7.38 (m, 10H, 2H-9, 2H-10, H-11, H-18, H-33, H-33', H-34, H-34'), 7.45 (d, *J* = 8.1 Hz, 1H, H-24), 7.62 (d, *J* = 7.8 Hz, 1H, H-31), 8.17 (d, *J* = 6.5, 1H, H-36), 8.24 (s, 1H, H-35), 9.03 (d, *J* = 8.1 Hz, 1H, H-42); ^{13}C NMR (100 MHz, $CDCl_3$) δ 16.0 (C-21), 17.8 (C-39), 21.5 (C-17), 21.7, 23.0 (C-47, C-47'), 24.5 (C-38), 24.9 (C-46), 26.0 (C-20), 28.4 (C-1), 31.4 (C-14, C-29), 31.9 (C-26), 32.6 (C-15, C-27), 37.3 (C-7), 40.3 (C-45), 43.3 (C-13), 48.0 (C-19, C-37), 48.2 (C-25), 49.4 (C-40), 49.9 (C-22), 51.1 (C-43), 52.0 (C-48), 56.9 (C-5), 81.6 (C-2), 111.1, 112.4, 118.9, 119.0, 121.6, , 127.5, 128.1, 129.0, 135.7 (C-9, C-10, C-11, C-31, C-33, C-33', C-34, C-34'), 122.7, 129.2 (C-30, C-32), 136.2 (C-8, C-32'), 156.5 (C-3), 171.3, 172.1, 173.6, 173.8 (C-6, C-16, C-23,

C-28, C-41, C-44); IR ν_{max} 1456, 1484, 1552, 1655, 1703, 1742, 2872, 2935, 2960, 3063, 3260 (br), 3329 (br), 3416, 3481 cm^{-1}; HRMS (ESI): $[M+Na]^+$, 922.5042 found, $C_{49}H_{69}N_7NaO_9$ 922.5049 calcd.

LCMS:

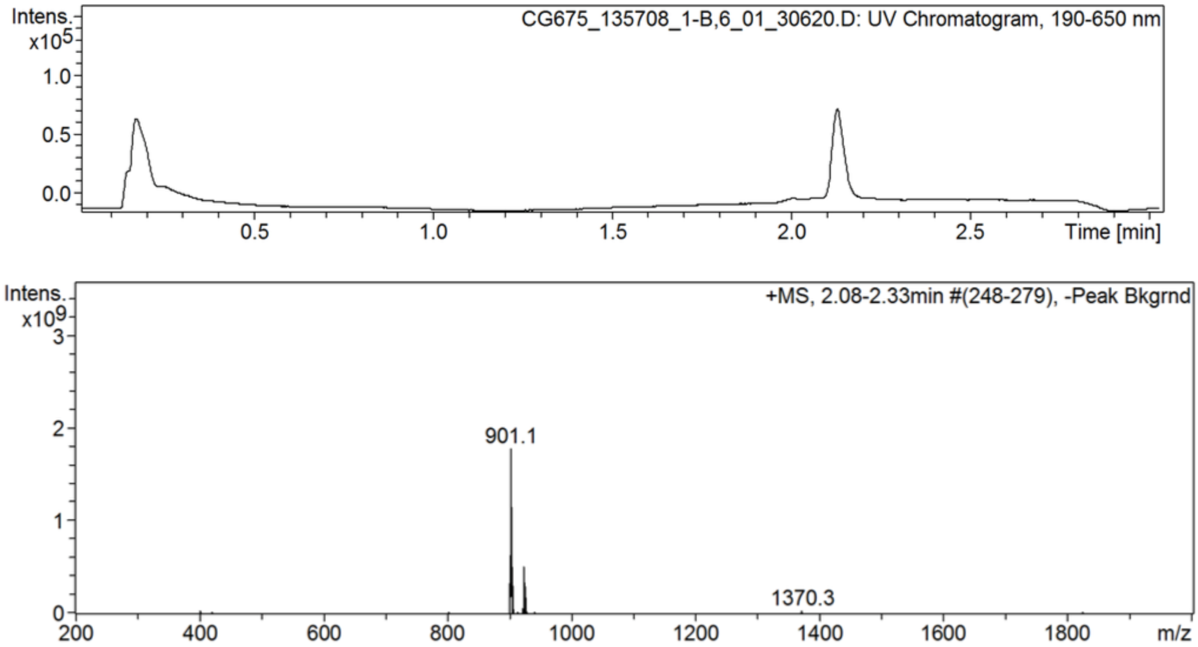

HPLC:

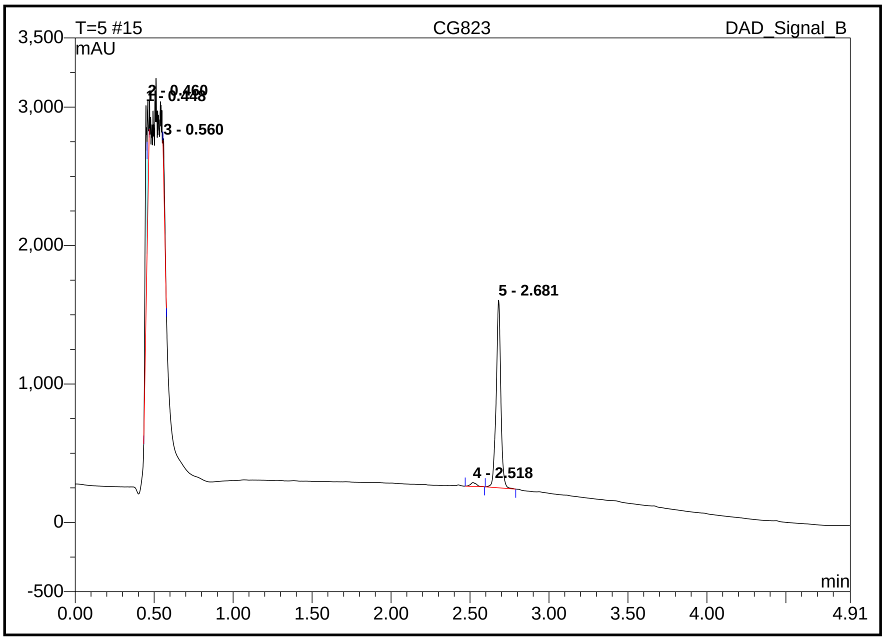

Boc–Phe–(*R*)-γ⁴-Ala–(1*S*,2*S*)-ACBC–(*R*)-γ⁴-Trp–(1*S*,2*S*)-ACBC–(*R*)-γ⁴-Leu–OMe 2

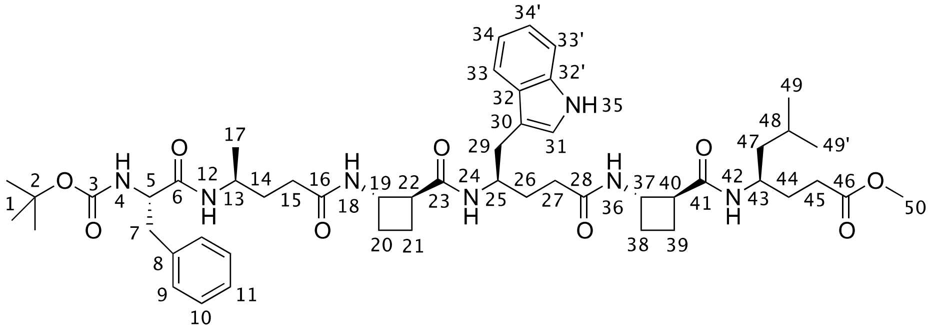

$C_{51}H_{73}N_7O_9$

M = 928.17 g.mol⁻¹

Following the general procedure A, methyl ester of **III** (119 mg, 0.25 mmol) was hydrolysed to give the corresponding carboxylic acid, Boc–Phe–(*R*)-γ⁴-Ala–(1*S*,2*S*)-ACBC–OH (112 mg, 97% crude yield). Following the general procedure B, **V** (150 mg, 0.26 mmol) was deprotected in 2.5 h to give the corresponding TFA salt, TFA·H_2N–(*R*)-γ⁴-Trp–(1*S*,2*S*)-ACBC–(*R*)-γ⁴-Leu–OMe. Following the general procedure C, the coupling reaction was performed with Boc–Phe–(*R*)-γ⁴-Ala–(1*S*,2*S*)-ACBC–OH (112 mg, 0.24 mmol), DIPEA (83 μL, 62 mg, 0.48 mmol) and HATU (95 mg, 0.25 mmol) in a mixture of CH_2Cl_2 / DMF (2 mL / 1 mL), and TFA·H_2N–(*R*)-γ⁴-Trp–(1*S*,2*S*)-ACBC–(*R*)-γ⁴-Leu–OMe (153 mg, 0.26 mmol), DIPEA (250 μL, 186 mg, 1.44 mmol) in DMF (2 mL) overnight. The purification was carried out by flash chromatography (gradient from 10 / 90 to 100 / 0 : EtOAc / PE then from 0 / 100 to 20 / 80 : CH_3OH / CH_2Cl_2) to give **2** as a yellow sticky solid (132 mg, 59%). The compound was further purified using mass-directed preparative HPLC (gradient from 5/95 to 95/5 of 0.1% formic acid water/methanol) to afford **2** with purity higher than 98%. R_f 0.50 (CH_3OH / CH_2Cl_2 = 10 / 90); $[\alpha]_D^{27}$= + 30 (c. 0.50 in CH_3OH); ¹H NMR (600 MHz, $CDCl_3$) δ 0.83 (d, *J* = 6.8 Hz, 3H, 3H-49), 0.84 (d, *J* = 6.7 Hz, 3H, 3H-49'), 1.01 (d, *J* = 5.9 Hz, 3H, 3H-17), 1.15-1.20 (m, 1H, H-47), 1.36-1.46 (m, 14H, 9H-1, 2H-14, 2H-26, H-47'), 1.59-1.64 (m, 1H, H-48), 1.66-1.71 (m, 1H, H-21), 1.71-1.76 (m, 2H, 2H-44), 1.84-1.98 (m, 3H, H-20, H-38, H-39), 2.02-2.23 (m, 7H, 2H-15, H-20', H-21', H-27, H-38', H-39'), 2.29-2.32 (m, 1H, H-27'), 2.43-2.56 (m, 2H, 2H-45), 2.76-2.80 (m, 1H, H-22), 2.92-2.93 (m, 2H, 2H-29), 3.01-3.06 (m, 3H, 2H-7, H-40), 3.65 (s, 3H, 3H-50), 3.84 (bs, 1H, H-13), 3.99 (bs, 1H, H-43), 4.24-4.28 (m, 1H H-5), 4.32-4.39 (m, 2H, H-25, H-37), 4.43-4.49 (m, 1H, H-19), 5.30 (bs, 1H, H-4), 6.15 (d, *J* = 8.6 Hz, 1H, H-12), 7.01-7.37 (m, 10H, 2H-9, 2H-10, H-11, H-18, H-33, H-33', H-34, H-34'), 7.48 (d, *J* = 8.9 Hz, 1H, H-24), 7.62 (d, *J* = 7.5 Hz, 1H, H-31), 8.17 (d, *J* = 6.1 Hz, 1H, H-36), 8.28 (d, *J* = 7.9 Hz, 1H, H-42), 8.44 (bs, 1H, H-35); ¹³C NMR (100 MHz, $CDCl_3$) δ 16.0 (C-21), 18.0 (C-15), 21.4 (C-17), 22.2, 23.1 (C-49, C-49'), 24.9 (C-48), 24.5, 26.0 (C-38, C-39), 28.4 (C-1), 29.9 (C-45), 30.8 (C-29), 31.3, 31.4 (C-14, C-26, C-44), 32.2 (C-27), 32.6 (C-20), 37.3 (C-7), 43.2 (C-13), 43.9 (C-47), 47.1 (C-43), 47.9, 48.0, 48.1 (C-19, C-25, C-37), 49.6, 49.7 (C-22, C-40), 51.5 (C-50), 57.1 (C-5), 81.5 (C-2), 111.1, 118.9, 120.0, 121.6, 122.7, 127.5, 129.0, 129.2 (C-9, C-10, C-11, C-31, C-33, C-33', C-34, C-34'), 112.3 (C-30), 128.1, 135.8, 136.2 (C-8, C-32, C-32'), 156.6 (C-3), 171.4, 172.1, 173.4, 173.6, 174.5 (C-6, C-16, C-23, C-28, C-41, C-46); IR ν_{max} 1455, 1484, 1552, 1655, 1704, 1726,

2872, 2930, 2957, 3065, 3265 (br), 3328 (br), 3416 , 3481 cm^{-1}; HRMS (ESI): $[M+Na]^+$, 950.5366 found, $C_{51}H_{73}N_7NaO_9$ 950.5362 calcd.

LCMS:

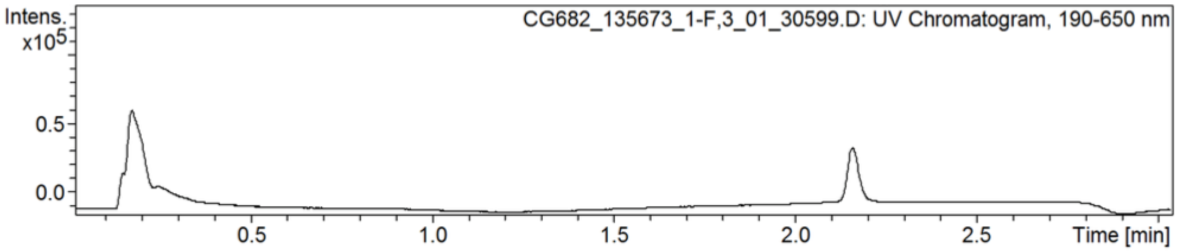

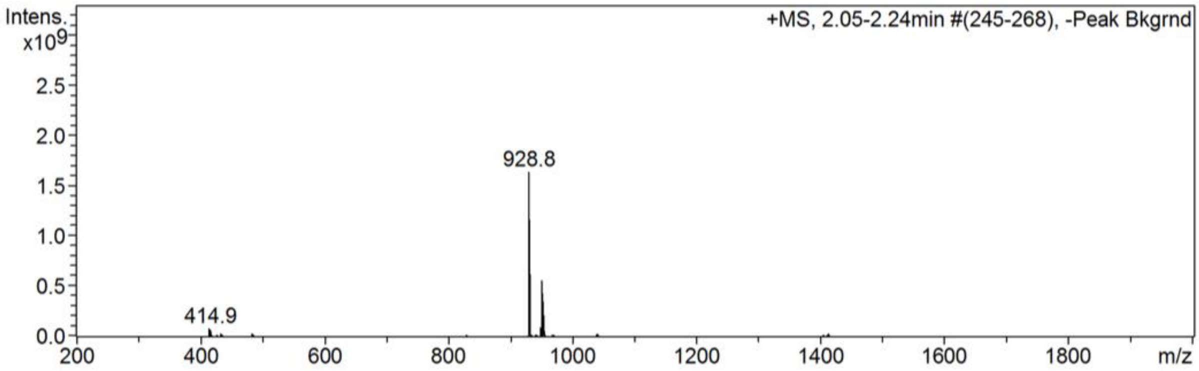

HPLC:

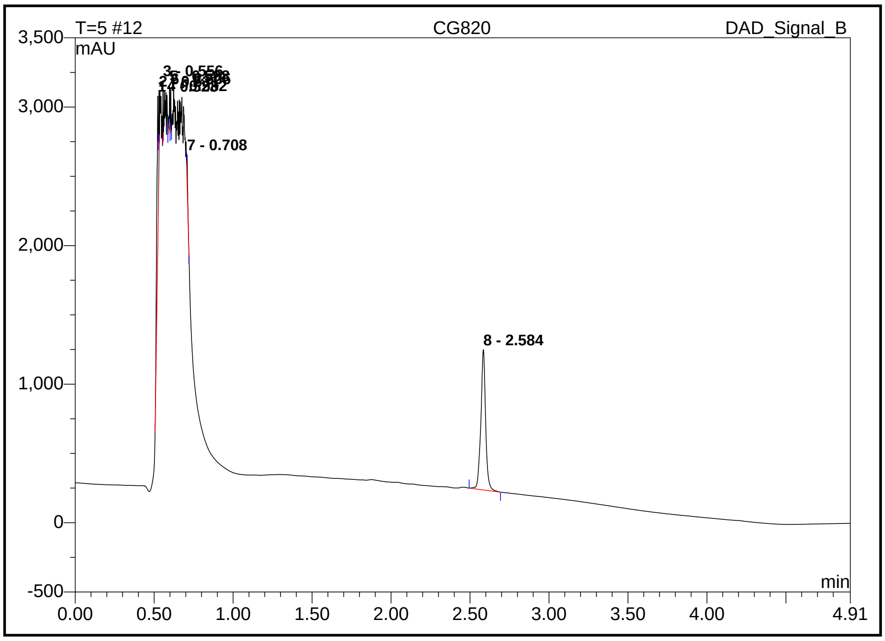

Boc–(*R*)-γ⁴-Phe–(1*S*,2*S*)-ACBC–OBn VI

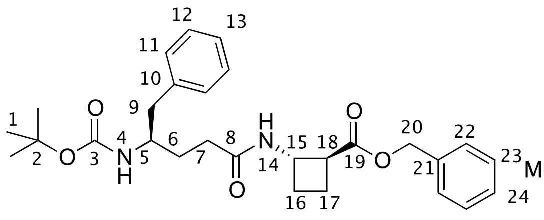

Following the general procedure A, Boc–(1*S*,2*S*)-ACBC–OBn (252 mg, 1.17 mmol) was deprotected in 1.5 h to give the corresponding TFA salt, TFA·H_2N–(1*S*,2*S*)-ACBC–OBn. Following the general procedure B, a solution of this material (268 mg, 1.17 mmol) and DIPEA (1.195 mL, 906 mg, 7.02 mmol) in CH_2Cl_2 (2 mL) was combined with a solution of Boc–(*R*)-γ^4-Phe–OH (342 mg, 1.17 mmol), DIPEA (400 μL, 302 mg, 2.34 mol) and HATU (461 mg, 1.23 mmol) in CH_2Cl_2/DMF (5 mL/2 mL) and left for 3 d. After work-up, chromatographic purification of the crude product (EtOAC/PE: gradient from 10/90 to 80/20) gave Boc–(*R*)-γ^4-Phe–(1*S*,2*S*)-ACBC–OBn **VI** as a sticky white solid (242 mg, 43%). R_f 0.15 (EtOAc/PE: 30/70); $[a]_D^{26}$= –115 (*c.* 0.10, $CHCl_3$); ^{1}H NMR (360 MHz, $CDCl_3$) δ 1.39 (s, 9H, 9H-1), 1.56-1.65 (m, 1H, H-6), 1.70-1.82 (m, 1H, H-6'), 1.95-1.99 (m, 3H, H-16, 2H-17), 2.15-2.20 (m, 3H, 2H-7, H-16'), 2.65 (m, 2H, 2H-9), 3.13-3.15 (m, 1H, H-18), 3.81 (m, 1H, H-5), 4.55-4.57 (m, 1H, H-15), 4.79 (d, 1H, *J* = 7.5 Hz, H-4), 5.11 (s, 2H, 2H-20), 7.15 (m, 1H, H-14), 7.12-7.32 (10H, m, 2H-11, 2H-12, H-13, 2H-22, 2H-23, H-24); ^{13}C NMR (90 MHz, $CDCl_3$) δ 18.5 (C-6), 26.9 (C-7), 28.3 (C-1), 30.8 (C-17), 33.2 (C-16), 41.6 (C-9), 46.7 (C-18), 47.5 (C-15), 51.3 (C-5), 66.3 (C-20), 79.3 (C-2), 126.4-129.3 (C-11, C-12, C-13, C-22, C-23, C-24), 136.0 (C-10), 137.9 (C-21), 156.3 (C-3), 172.4 (C-8), 173.0 (C-19); IR ν_{max} 1454, 1505, 1603, 1670, 1697, 1725, 2870, 2981, 3031, 3066, 3087, 3302(br), 3436 cm^{-1}; HRMS (ESI): $[M+Na]^+$, 503.2536 found, $C_{28}H_{36}N_2NaO_5$ 503.2516 calcd.

Boc–(*R*)-γ⁴-Phe–(1*S*,2*S*)-ACBC–Leu–OMe VII

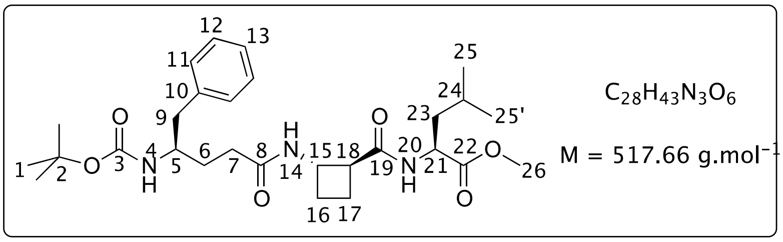

To a solution of **VI** (105 mg, 0.26 mmol) in CH_2Cl_2 (8 mL) was added 10 % Pd-C (10% w/w, 50 mg, cat.). The black suspension was stirred under a H_2 atmosphere for 2 h. The mixture was then filtered through a celite pad and washed through with CH_2Cl_2. The filtrate was concentrated under reduced pressure to afford Boc–Phe–(*R*)-γ^4-Phe–(1*S*,2*S*)-ACBC–OH (95 mg, 95% crude yield). Following the general procedure C, the coupling reaction was performed with Boc–(*R*)-γ^4-Phe–(1*S*,2*S*)-ACBC–OH (95 mg, 0.24 mmol), DIPEA (83 µL, 62 mg, 0.48 mmol) and HATU (96 mg, 0.26 mmol) in a mixture of CH_2Cl_2 / DMF (1 mL / 1 mL), and H_2N–Leu–OMe (47 mg, 0.26 mmol), DIPEA (83 µL, 62 mg, 0.48 mmol) in CH_2Cl_2 (2 mL) overnight. The purification was carried out by flash chromatography (gradient from 10 / 90 to 80 / 20 : EtOAC / PE) to give **VII** as a white sticky solid (102 mg, 82%). R_f 0.60 (CH_3OH / CH_2Cl_2 = 10 / 90); $[\alpha]_D^{24}$= + 19 (c. 0.50 in $CHCl_3$); ^1H NMR (360 MHz, $CDCl_3$) δ 0.89 (d, *J* = 6.0 Hz, 3H, 3H-25), 0.91 (d, *J* = 6.9 Hz, 3H, 3H-25'), 1.40 (s, 9H, 9H-1), 1.55-1.64 (m, 4H, H-6, 2H-23, H-24), 1.84-2.07 (m, 5H, H-6', H-7, H-16, 2H-17), 2.17-2.24 (m, 2H, H-7', H-16'), 2.76 (d, *J* = 6.4 Hz, 2H, 2H-9), 2.92-2.99 (m, 1H, H-18), 3.72 (s, 3H, 3H-26), 3.85 (bs, 1H, H-5), 4.34-4.42 (m, 1H, H-15), 4.46-4.52 (m, 1H, H-21), 4.64 (d, *J* = 9.5 Hz, 1H, H-4), 7.10 (d, *J* = 6.2 Hz, 1H, H-14), 7.13-7.29 (m, 5H, 2H-11, 2H-12, H-13), 8.60 (d, *J* = 7.5 Hz, 1H, H-20); ^{13}C NMR (90 MHz, $CDCl_3$) δ 18.3 (C-17), 21.7, 22.9 (C-25, C-25'), 24.4 (C-16), 24.9 (C-24), 28.4 (C-1), 31.1 (C-6), 32.7 (C-7), 40.5 (C-23), 41.8 (C-9), 48.0 (C-15), 49.3 (C-18), 50.7 (C-5), 50.9 (C-21), 52.2 (C-26), 79.7 (C-2), 126.6, 128.5, 129.3 (C-11, C-12, C-13), 137.5 (C-10), 156.4 (C-3), 173.3 (C-19), 173.7 (C-8), 173.8 (C-22); IR ν_{max} 1453, 1469, 1508, 1552, 1603, 1657, 1694, 1741, 2872, 2935, 2959, 3033, 3067, 3211, 3266 (br), 3436 cm^{-1}; HRMS (ESI): $[M+Na]^+$, 540.3040 found, $C_{28}H_{43}N_3NaO_6$ 540.3044 calcd.

Boc–(*R*)-γ4-Phe–(1*S*,2*S*)-ACBC–(*R*)-γ4-Leu–OMe VIII

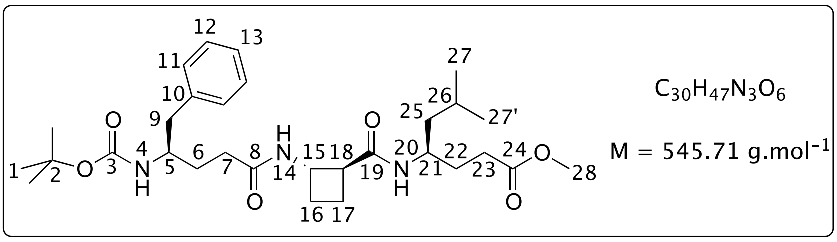

To a solution of **VI** (105 mg, 0.26 mmol) in CH_2Cl_2 (8 mL) was added 10 % Pd-C (10% w/w, 50 mg, cat.). The black suspension was stirred under a H_2 atmosphere for 2 h. The mixture was then filtered through a celite pad and washed through with CH_2Cl_2. The filtrate was concentrated under reduced pressure to afford Boc–Phe–(*R*)-γ4-Phe–(1*S*,2*S*)-ACBC–OH (95 mg, 95% crude yield). Following the general procedure C, the coupling reaction was performed with Boc–(*R*)-γ4-Phe–(1*S*,2*S*)-ACBC–OH (95 mg, 0.24 mmol), DIPEA (87 μL, 65 mg, 0.50 mmol) and HATU (98 mg, 0.26 mmol) in a mixture of CH_2Cl_2 / DMF (2 mL / 1 mL), and H_2N–(*R*)-γ4-Leu–OMe (59 mg, 0.30 mmol), DIPEA (175 μL, 129 mg, 1.00 mmol) in CH_2Cl_2 (2 mL) overnight. The purification was carried out by flash chromatography (gradient from 10 / 90 to 80 / 20 : EtOAC / PE) to give **VIII** as a white sticky solid (125 mg, 92%). R_f 0.55 (CH_3OH / CH_2Cl_2 = 10 / 90); $[\alpha]_D^{24}$= + 1.8 (c. 0.50 in CH_3OH); ^{1}H NMR (250 MHz, $CDCl_3$) δ 0.85 (d, *J* = 6.4 Hz, 3H, 3H-27), 0.86 (d, *J* = 6.1 Hz, 3H, 3H-27'), 1.22-1.27 (m, 1H, H-25), 1.39 (s, 10H, 9H-1, H-25'), 1.48-1.75 (m, 4H, 2H-6, H-22, H-26), 1.82-1.98 (m, 3H, H-16, H-17, H-22'), 2.06-2.31 (m, 4H, 2H-7, H-16', H-17'), 2.41 (t, *J* = 7.3 Hz, 2H, 2H-23), 2.76 (d, *J* = 5.6 Hz, 2H, 2H-9), 2.89-2.98 (m, 1H, H-18), 3.67 (s, 3H, 3H-28), 3.82 (bs, 1H, H-5), 3.91-3.98 (m, 1H, H-21), 4.24-4.36 (m, 1H, H-15), 4.65 (d, *J* = 9.1 Hz, 1H, H-4), 7.14-7.32 (m, 6H, 2H-11, 2H-12, H-13, H-14), 8.04 (d, *J* = 8.8 Hz, 1H, H-20); ^{13}C NMR (62.5 MHz, $CDCl_3$) δ 18.6 (C-17), 22.1, 23.1 (C-27, C-27'), 24.4 (C-16), 25.0 (C-26), 28.3 (C-1), 30.8, 30.9 (C-6, C-22, C-23), 32.7 (C-7), 41.8 (C-9), 43.9 (C-25), 46.9 (C-21), 47.9 (C-15), 49.7 (C-18), 50.7 (C-5), 51.7 (C-28), 79.7 (C-2), 126.6, 128.5, 129.3 (C-11, C-12, C-13), 137.5 (C-10), 156.3 (C-3), 173.3, 173.9, 174.5 (C-8, C-19, C-24); IR ν_{max} 1440, 1451, 1507, 1555, 1564, 1649, 1699, 1730, 2872, 2957, 3030, 3070, 3194, 3267, 3340 (br), 3435 cm^{-1}; HRMS (ESI): $[M+Na]^+$, 568.3383 found, $C_{30}H_{47}N_3NaO_6$ 568.3357 calcd.

Boc–Phe–(*R*)-γ⁴-Ala–(1*S*,2*S*)-ACBC–(*R*)-γ⁴-Phe–(1*S*,2*S*)-ACBC–Leu–OMe 3

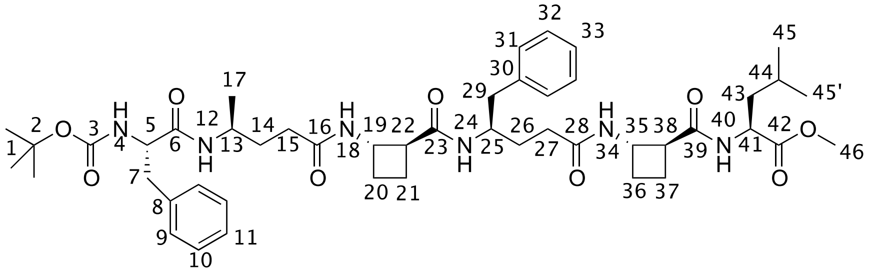

$C_{47}H_{68}N_6O_9$

M = 861.08 g.mol^{-1}

Following the general procedure A, methyl ester of **II** (67 mg, 0.14 mmol) was hydrolysed to give the corresponding carboxylic acid, Boc–Phe–(*R*)-γ^4-Ala–(1*S*,2*S*)-ACBC–OH (66 mg, 98% crude yield). Following the general procedure B, **VII** (70 mg, 0.14 mmol) was deprotected in 2.5 h to give the corresponding TFA salt, TFA·H_2N–(*R*)-γ^4-Phe–(1*S*,2*S*)-ACBC–Leu–OMe. Following the general procedure C, the coupling reaction was performed with Boc–Phe–(*R*)-γ^4-Ala–(1*S*,2*S*)-ACBC–OH (66 mg, 0.14 mmol), DIPEA (48 μL, 36 mg, 0.28 mmol) and HATU (55 mg, 0.15 mmol) in a mixture of CH_2Cl_2 / DMF (2 mL / 1 mL), and TFA·H_2N–(*R*)-γ^4-Phe–(1*S*,2*S*)-ACBC–Leu–OMe (72 mg, 0.14 mmol), DIPEA (146 μL, 108 mg, 0.84 mmol) in a mixture of CH_2Cl_2 / DMF (2 mL / 1 mL) overnight. The purification was carried out by flash chromatography (gradient from 0 / 100 to 10 / 90 : CH_3OH / CH_2Cl_2) to give **3** as a white sticky solid (94 mg, 78%). The compound was further purified using mass-directed preparative HPLC (gradient from 5/95 to 95/5 of 0.1% formic acid water/methanol) to afford **3** with purity higher than 95%. R_f 0.58 (CH_3OH / CH_2Cl_2 = 10 / 90); $[\alpha]_D^{26}$= + 17 (c. 0.50 in $CHCl_3$); ^1H NMR (600 MHz, $CDCl_3$) δ 0.91 (d, *J* = 6.4 Hz, 6H, 6H-45), 1.05 (d, *J* = 6.5 Hz, 3H, 3H-17), 1.36-1.43 (m, 4H, 2H-14, 2H-26), 1.46 (s, 9H, 9H-1), 1.62-1.73 (m, 4H, H-21, 2H-43, H-44), 1.88-2.08 (m, 6H, H-15, H-20, H-27, H-36, 2H-37), 2.12-2.26 (m, 5H, H-15', H-20', H-21', H-27', H-36'), 2.77-2.79 (m, 3H, H-22, 2H-29), 3.04-3.08 (m, 1H, H-38), 3.13- 3.14 (m, 2H, 2H-7), 3.73 (s, 3H, 3H-46), 3.85 (bs, 1H, H-13), 4.23-4.32 (m, 2H, H-5, H-25), 4.41-4.44 (m, 1H, H-35), 4.47-4.50 (m, 1H, H-19), 4.50-4.54 (m, 1H, H-41), 5.10 (d, *J* = 3.8 Hz, 1H, H-4), 5.96 (d, *J* = 8.9 Hz, 1H, H-12), 7.15-7.40 (m, 11H, 2H-9, 2H-10, H-11, H-18, 2H-31, 2H-32, H-33), 7.47 (d, *J* = 9.5 Hz, 1H, H-24), 8.17 (d, *J* = 6.5 Hz, 1H, H-34), 8.98 (d, *J* = 7.7 Hz, 1H, H-40); ^{13}C NMR (100 MHz, $CDCl_3$) δ 15.9 (C-21), 17.8 (C-37), 21.5 (C-17), 21.7, 23.0 (C-45, C-45'), 24.4 (C-36), 24.9 (C-44), 26.1 (C-20), 28.3 (C-1), 31.4 (C-14, C-26), 31.8, 32.6 (C-15, C-27), 37.4 (C-7), 40.4 (C-43), 42.0 (C-29), 43.3 (C-13), 48.0 (C-19, C-35), 48.7 (C-25), 49.4 (C-38), 49.8 (C-22), 51.1 (C-41), 52.0 (C-5), 81.7 (C-2), 126.1, 127.6, 128.2, 129.1, 129.2, 129.4 (C-9, C-10, C-11, C-31, C-32, C-33), 135.6, 138.4 (C-8, C-30), 156.5 (C-3), 171.2, 171.3, 171.9, 173.5, 173.8 (C-6, C-16, C-23, C-28, C-39, C-42); IR ν_{max} 1454, 1483, 1550, 1654, 1703, 1743, 2871, 2960, 3030, 3067, 3263 (br), 3322 (br), 3416 cm^{-1}; HRMS (ESI): $[M+Na]^+$, 883.4936 found, $C_{47}H_{68}N_6NaO_9$ 883.4940 calcd.

LCMS:

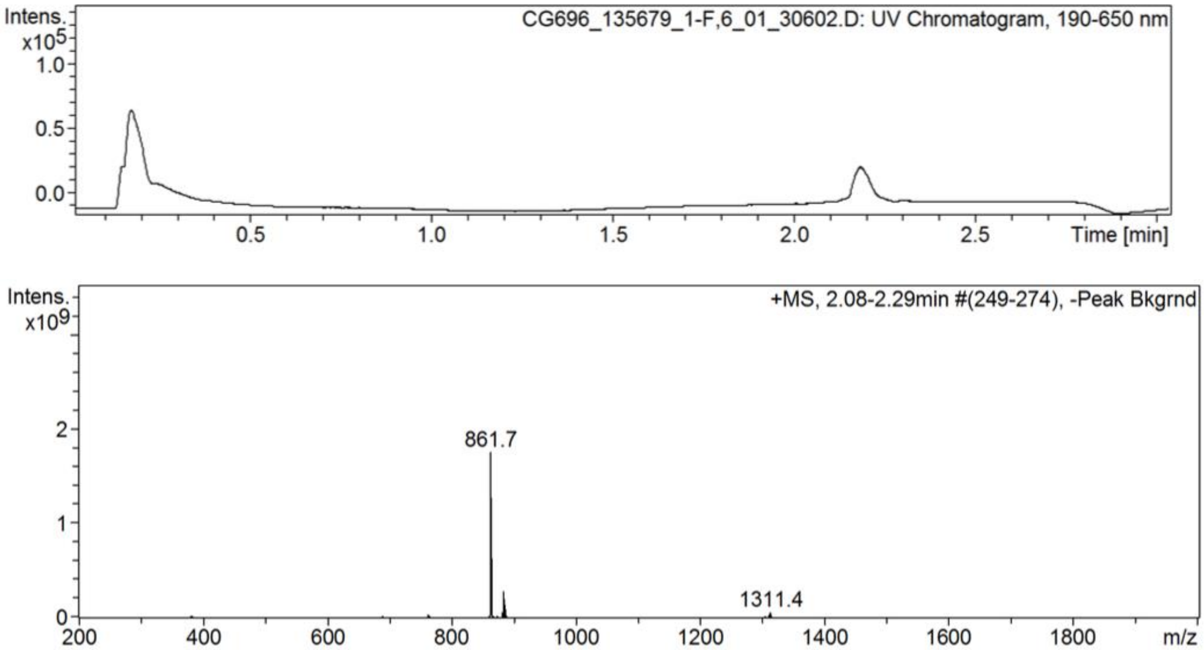
CG696_135679_1-F,6_01_30602.D: UV Chromatogram, 190-650 nm
Intens.
x10^5
1.0
0.5
0.0
0.5
1.0
1.5
2.0
2.5
Time [min]
+MS, 2.08-2.29min #(249-274), -Peak Bkgrnd
Intens.
x10^9
2
1
0
861.7
1311.4
200
400
600
800
1000
1200
1400
1600
1800
m/z

HPLC:

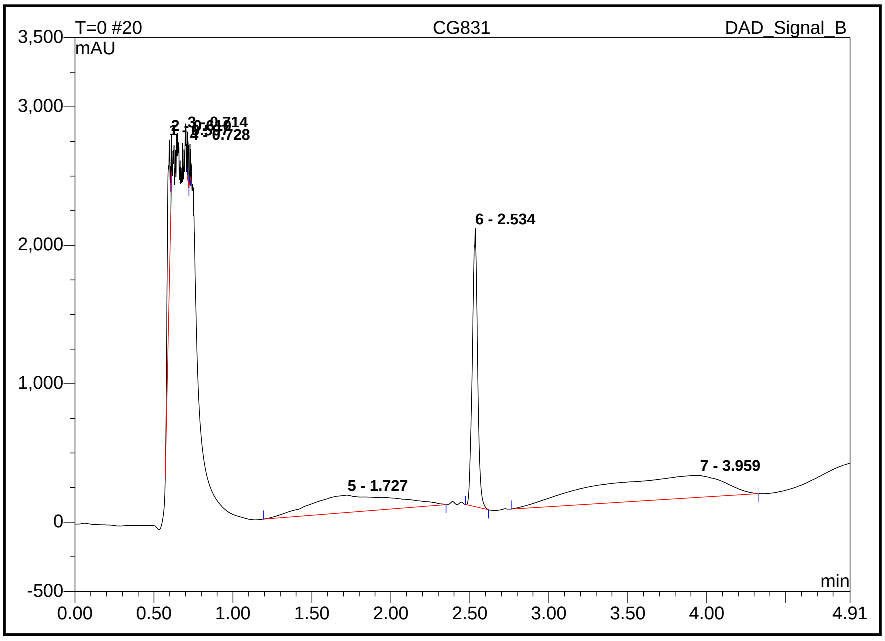
T=0 #20
CG831
DAD_Signal_B
mAU
3,500
3,000
2,000
1,000
0
-500
6 - 2.534
5 - 1.727
7 - 3.959
min
0.00
0.50
1.00
1.50
2.00
2.50
3.00
3.50
4.00
4.91

Boc–Phe–(*R*)-γ⁴-Ala–(1*S*,2*S*)-ACBC–(*R*)-γ⁴-Phe–(1*S*,2*S*)-ACBC–(*R*)-γ⁴-Leu–OMe 4

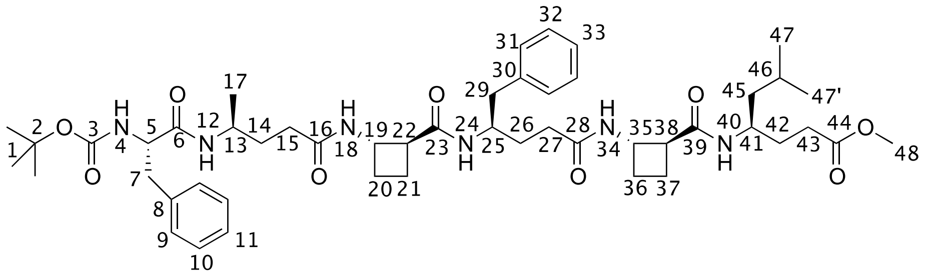

$C_{49}H_{72}N_6O_9$

M = 889.13 g.mol^{-1}

Following the general procedure A, methyl ester of **II** (57 mg, 0.12 mmol) was hydrolysed to give the corresponding carboxylic acid, Boc–Phe–(*R*)-γ^4-Ala–(1*S*,2*S*)-ACBC–OH (55 mg, 96% crude yield). Following the general procedure B, **VIII** (105 mg, 0.19 mmol) was deprotected in 2.2 h to give the corresponding TFA salt, TFA·H_2N–(*R*)-γ^4-Phe–(1*S*,2*S*)-ACBC–(*R*)-γ^4-Leu–OMe. Following the general procedure C, the coupling reaction was performed with Boc–Phe–(*R*)-γ^4-Ala–(1*S*,2*S*)-ACBC–OH (55 mg, 0.12 mmol), DIPEA (41 μL, 30 mg, 0.24 mmol) and HATU (49 mg, 0.13 mmol) in a mixture of CH_2Cl_2 / DMF (2 mL / 1 mL); and TFA·H_2N–(*R*)-γ^4-Phe–(1*S*,2*S*)-ACBC–(*R*)-γ^4-Leu–OMe (108 mg, 0.19 mmol), DIPEA (195 μL, 144 mg, 1.15 mmol) in a mixture of CH_2Cl_2 / DMF (2 mL / 1 mL) overnight. The purification was carried out by flash chromatography (gradient from 0 / 100 to 10 / 90 : CH_3OH / CH_2Cl_2) to give **4** as a white sticky solid (90 mg, 84%). The compound was further purified using mass-directed preparative HPLC (gradient from 5/95 to 95/5 of 0.1% formic acid water/methanol) to afford the **4** with purity higher than 95%. R_f 0.63 (CH_3OH / CH_2Cl_2 = 10 / 90); $[\alpha]_D^{26}$= + 12 (c. 0.50 in CH_3OH); ^{1}H NMR (600 MHz, $CDCl_3$) δ 0.85 (d, *J* = 6.8 Hz, 3H, 3H-47), 0.87 (d, *J* = 6.0 Hz, 3H, 3H-47'), 1.04 (d, *J* = 6.0 Hz, 3H, 3H-17), 1.18-1.22 (m, 1H, H-45), 1.36-1.43 (m, 5H, 2H-14, 2H-26, H-45'), 1.46 (s, 9H, 9H-1), 1.58-1.62 (m, 1H, H-46), 1.69-1.74 (m, 3H, H-21, 2H-42), 1.87-2.03 (m, 7H, H-20, 2H-16, H-27, H-36, 2H-37), 2.09-2.18 (m, 4H, H-20', H-21', H-27', H-36'), 2.44-2.52 (m, 2H, 2H-43), 2.76-2.81 (m, 3H, H-22, 2H-29), 2.96-3.00 (m, 1H, H-38), 3.12 (d, *J* = 6.3 Hz, 2H, 2H-7), 3.67 (s, 3H, 3H-48), 3.84 (bs, 1H, H-13), 3.99 (bs, 1H, H-41), 4.25 (bs, 1H, H-25), 4.28-4.31 (m, 1H, H-5), 4.33-4.36 (m, 1H, H-35), 4.44-4.47 (m, 1H, H-19), 5.18 (bs, 1H, H-4), 6.05 (d, *J* = 9.2 Hz, 1H, H-12), 7.14-7.39 (m, 11H, 2H-9, 2H-10, H-11, H-18, 2H-31, 2H-32, H-33), 7.48 (d, *J* = 8.8 Hz, 1H, H-24), 8.17 (d, *J* = 7.3 Hz, 1H, H-34), 8.23 (d, *J* = 8.5 Hz, 1H, H-40); ^{13}C NMR (100 MHz, $CDCl_3$) δ 15.7 (C-21), 17.6 (C-37), 21.3 (C-17), 22.2, 23.0 (C-47, C-47'), 24.4 (C-36), 24.8 (C-46), 26.0 (C-20), 28.2 (C-1), 30.6, 30.8 (C-42, C-43), 31.1, 31.4, 32.2, 32.5 (C-14, C-15, C-26, C-27), 37.2 (C-7), 41.7 (C-29), 43.1 (C-13), 43.9 (C-45), 46.5 (C-41), 47.8, 47.9 (C-19, C-35), 48.4 (C-25), 49.5 (C-22), 49.9 (C-38), 51.3 (C-48), 57.0 (C-5), 81.5 (C-2), 126.0, 127.4, 128.0, 128.9, 129.1, 129.3 (C-9, C-10, C-11, C-31, C-32, C-33), 135.4, 138.2 (C-8, C-30), 156.4 (C-3), 171.1, 171.3, 171.7 (C-6, C-16, C-23), 172.9 (C-39), 173.1 (C-28), 174.4 (C-44); IR ν_{max} 1454, 1484, 1494, 1522, 1552, 1654, 1703, 1727, 2870, 2930,

2957, 3027, 3066, 3260 (br), 3321 (br), 3416 cm^{-1}; HRMS (ESI): [M+Na]$^+$, 911.5271 found, $C_{49}H_{72}N_6NaO_9$ 911.5253 calcd.

LCMS:

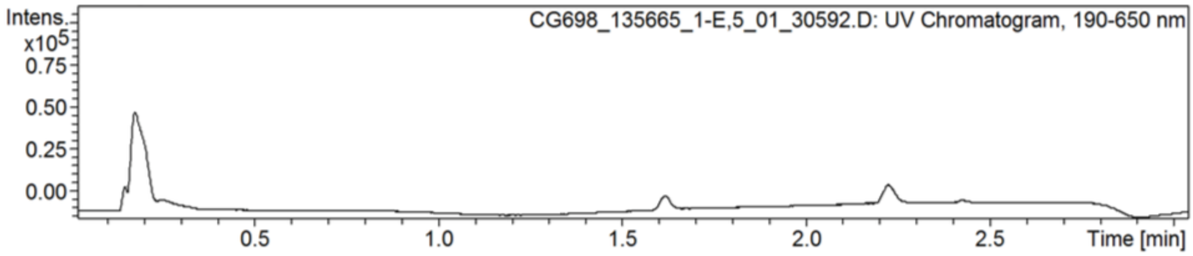

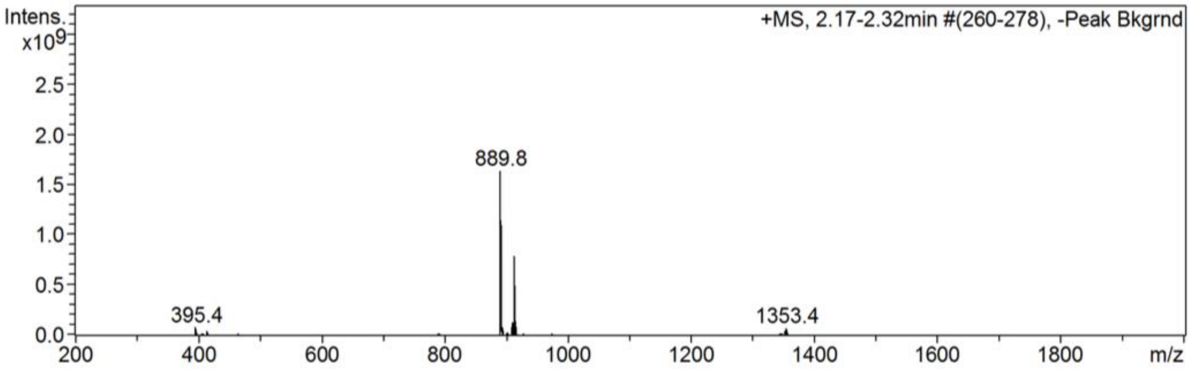

HPLC:

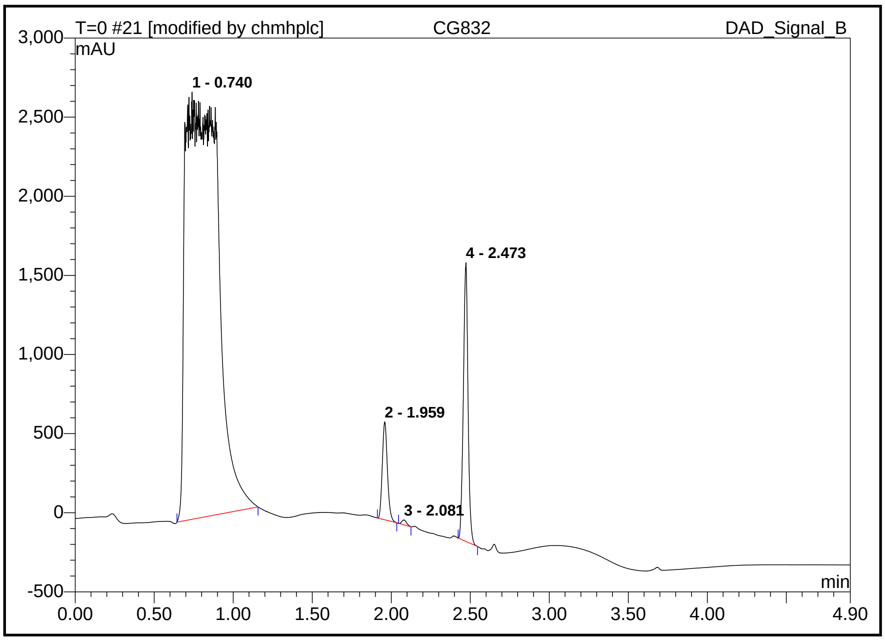

Ac–Phe–(*R*)-γ⁴-Ala–(1*S*,2*S*)-ACBC–(*R*)-γ⁴-Trp–(1*S*,2*S*)-ACBC–Leu–OMe – 5

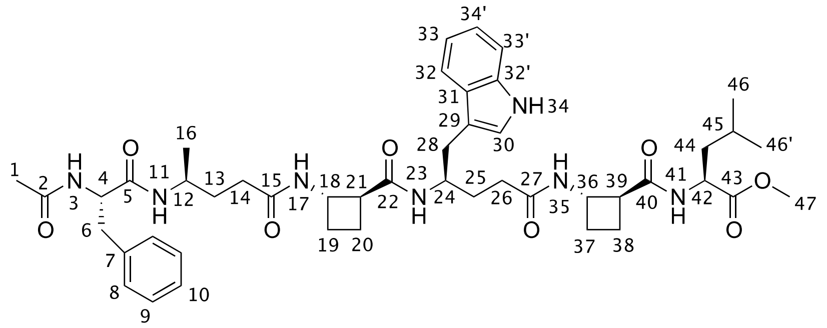

$C_{46}H_{63}N_7O_8$

M = 842.03 g.mol^{-1}

Following the general procedure B, **1** (64 mg, 0.07 mmol) was deprotected in 5 h to give the corresponding TFA salt, TFA·H_2N–Phe–(*R*)-γ^{4}Ala–(1*S*,2*S*)-ACBC–(*R*)-γ^{4}Trp–(1*S*,2*S*)-ACBC–Leu–OMe. Following the general procedure D, the acetylation was performed with the generated TFA salt (56 mg, 0.07 mmol), DIPEA (73 μL, 54 mg, 0.42 mmol) and acetic anhydride (9 μL, 11 mg, 0.11 mmol) in CH_2Cl_2 (2 mL) overnight to give peptide **5** (25 mg, 42%). The compound was purified using mass-directed preparative HPLC (gradient from 5/95 to 95/5 of 0.1% formic acid water/methanol) to afford **5** with purity higher than 95%. R_f 0.63 (CH_3OH / CH_2Cl_2 = 10 / 90); ^{1}H NMR (600 MHz, CD_3OD) δ 0.83 (d, *J* = 6.4 Hz, 3H), 0.85 (d, *J* = 6.6 Hz, 3H), 1.08 (d, J = 6.4 Hz, 3H), 1.22-1.25 (m, 1H), 1.55-1.62 (m, 5H), 1.72-1.82 (m, 5H), 1.85-2.01 (m, 11H), 2.09-2.19 (m, 4H), 2.39-2.43 (m, 2H), 2.79-2.85 (m, 2H), 2.94 (d, *J* = 7.1 Hz, 1H), 2.97 (d, *J* = 8.0 Hz, 1H), 3.02-3.06 (m, 1H), 3.10 (d, *J* = 6.7 Hz, 1H), 3.12 (d, *J* = 6.7 Hz, 1H), 3.67 (s, 3H), 3.83 (bs, 1H), 3.91 (bs, 1H), 4.11 (bs, 1H), 4.36-4.42 (m, 2H), 4.51-4.56 (m, 1H), 6.93-6.99 (m, 1H), 7.04-7.07 (m, 2H), 7.21-7.23 (m, 2H), 7.27-7.30 (m, 4H), 7.53 (d, *J* = 8.1 Hz, 1H); ^{13}C NMR (100 MHz, CD_3OD) δ 16.6, 17.4, 20.0, 20.9, 21.2, 22.3, 24.6, 30.1, 30.3, 30.9, 31.5, 31.8, 31.9, 37.5, 43.4, 44.1, 46.5, 47.9, 48.6, 49.1, 49.5, 50.6, 55.6, 110.5, 111.0, 118.1, 118.3, 120.7, 122.8, 126.7, 127.9, 128.2, 128.9, 136.6, 137.0 , 171.6, 172.1, 173.1, 173.5, 173.7, 174.3; HRMS (ESI): [M+Na]$^+$, 864.4644 found, $C_{46}H_{63}N_7NaO_8$ calcd 864.4636.

LCMS:

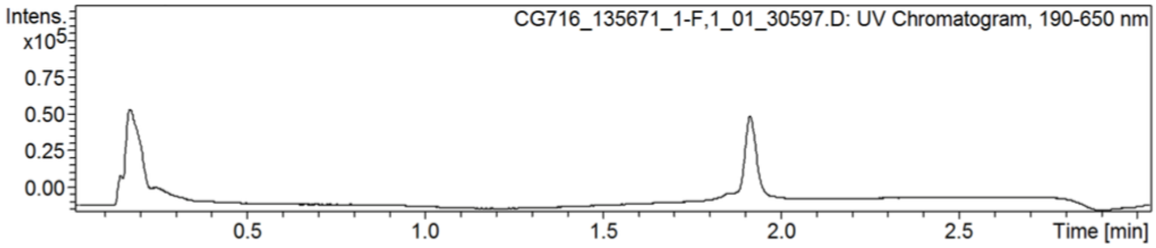

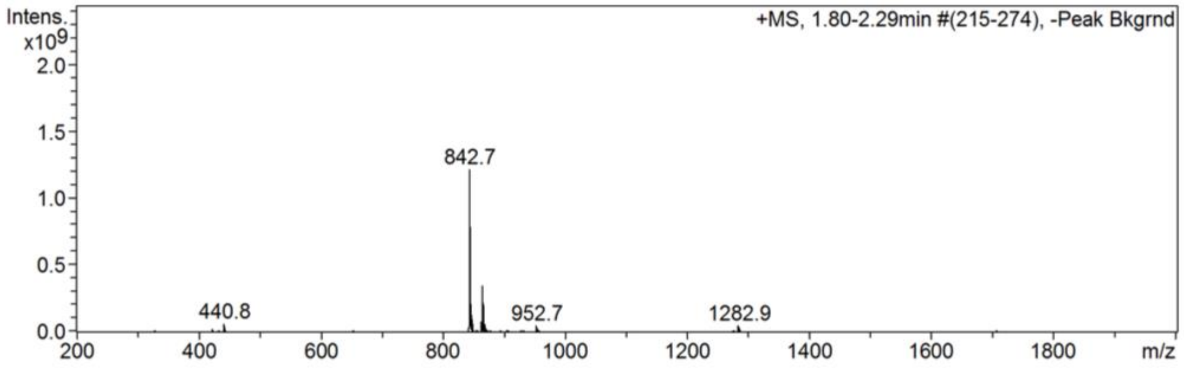
Intens.
x10^9
+MS, 1.80-2.29min #(215-274), -Peak Bkgrnd
842.7
440.8
952.7
1282.9
m/z

HPLC:

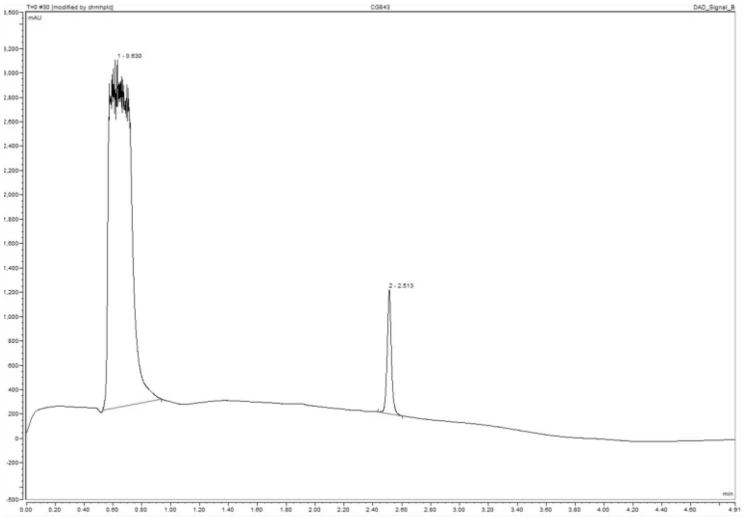
T=0 #30 [modified by chmhplc]
CG843
DAD_Signal_B
mAU
1 - 0.630
2 - 2.513
min

Ac–Phe–(*R*)-γ⁴-Ala–(1*S*,2*S*)-ACBC–(*R*)-γ⁴-Trp–(1*S*,2*S*)-ACBC–(*R*)-γ⁴-Leu–OMe 6

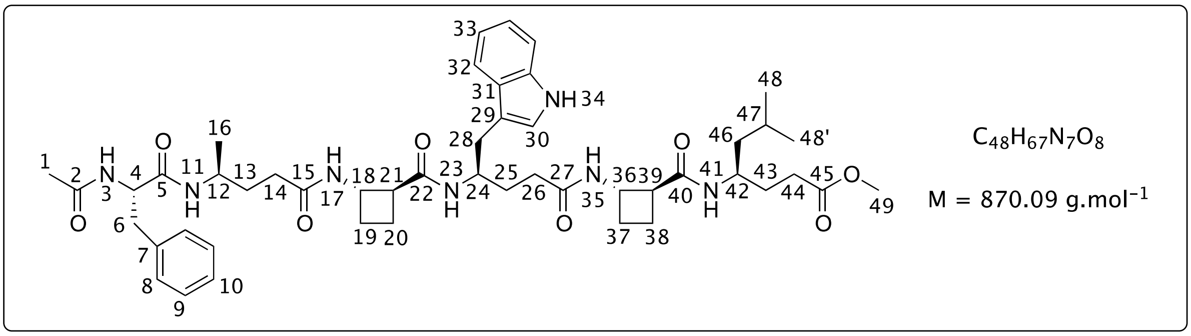

Following the general procedure C, **2** (50 mg, 0.05 mmol) was deprotected in 5 h to give the corresponding TFA salt, TFA·H_2N–Phe–(*R*)-γ⁴Ala–(1*S*,2*S*)-ACBC–(*R*)-γ⁴Trp–(1*S*,2*S*)-ACBC–Leu–OMe. Following the general procedure E, the acetylation was performed with the generated TFA salt (44 mg, 0.05 mmol), DIPEA (52 μL, 39 mg, 0.3 mmol) and acetic anhydride (7 μL, 8 mg, 0.075 mmol) in CH_2Cl_2 (2 mL) overnight to give peptide **6** (14 mg, 32%). The compound was purified using mass-directed preparative HPLC (gradient from 5/95 to 95/5 of 0.1% formic acid water/methanol) to afford **6** with purity higher than 95%. R_f 0.63 (CH_3OH / CH_2Cl_2 = 10 / 90); ^1H NMR (600 MHz, CD_3OD) δ 0.83 (d, *J* = 6.4 Hz, 3H), 0.85 (d, *J* = 6.6 Hz, 3H), 1.08 (d, *J* = 6.4 Hz, 3H), 1.22-1.25 (m, 1H), 1.55-1.62 (m, 5H), 1.72-1.82 (m, 5H), 1.85-2.01 (m, 11H), 2.09-2.19 (m, 4H), 2.39-2.43 (m, 2H), 2.79-2.85 (m, 2H), 2.94 (d, *J* = 7.1 Hz, 1H), 2.97 (d, *J* = 8.0 Hz, 1H), 3.02-3.06 (m, 1H), 3.10 (d, *J* = 6.7 Hz, 1H), 3.12 (d, *J* = 6.7 Hz, 1H), 3.67 (s, 3H), 3.83 (bs, 1H), 3.91 (bs, 1H), 4.11 (bs, 1H), 4.36-4.42 (m, 2H), 4.51-4.56 (m, 1H), 6.93-6.99 (m, 1H), 7.04-7.07 (m, 2H), 7.21-7.23 (m, 2H), 7.27-7.30 (m, 4H), 7.53 (d, *J* = 8.1 Hz, 1H); ^{13}C NMR (100 MHz, CD_3OD) δ 16.6, 17.1, 19.8, 20.9, 21.1, 22.3, 24.3, 24.6, 24.7, 30.1, 30.3, 30.8, 31.5, 31.8, 31.9, 37.5, 43.4, 44.1, 46.5, 47.7, 48.6, 49.1, 49.5, 50.6, 55.4, 55.6, 110.8, 111.0, 118.1, 118.3, 120.7, 122.8, 126.5, 127.9, 128.1, 128.9, 136.6, 137.0 , 171.6, 172.1, 173.1, 173.5, 173.6, 174.5; HRMS (ESI): $[M+Na]^+$, 892.4942 found, $C_{48}H_{67}N_7NaO_8$ calcd 892.4949.

LCMS:

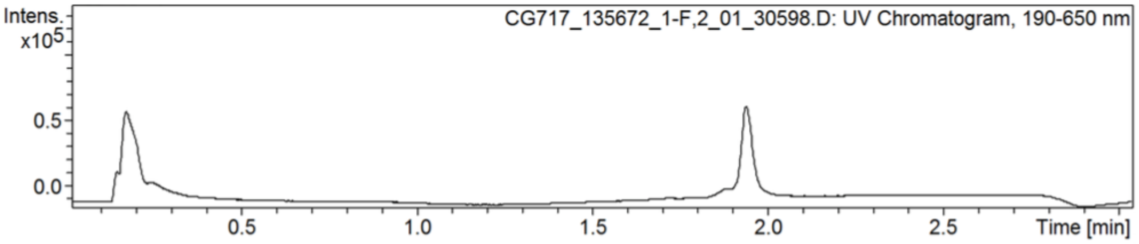

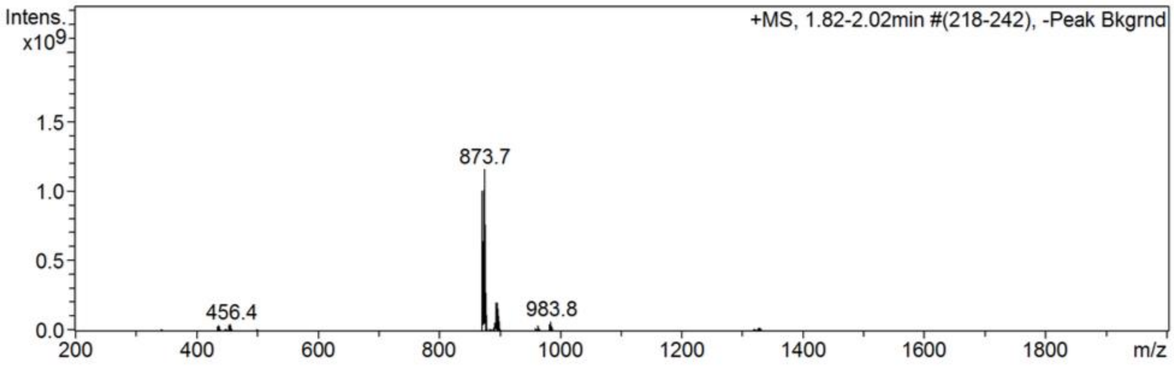
Intens.
x10^9
+MS, 1.82-2.02min #(218-242), -Peak Bkgrnd
1.5
1.0
0.5
0.0
873.7
456.4
983.8
200
400
600
800
1000
1200
1400
1600
1800
m/z

HPLC:

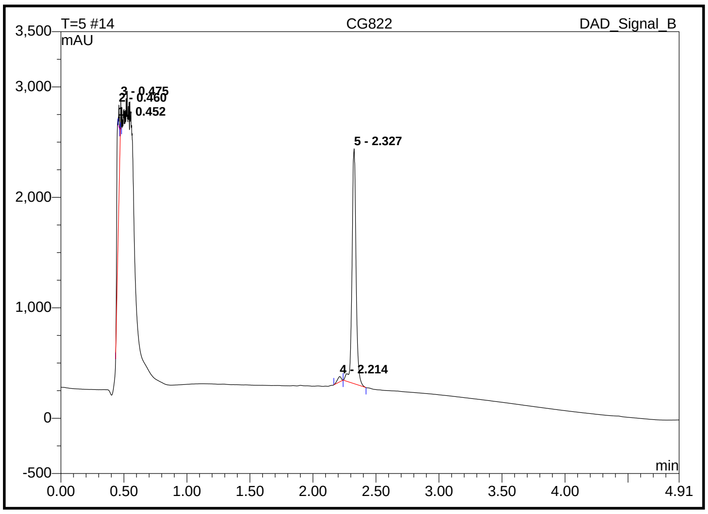
T=5 #14
CG822
DAD_Signal_B
mAU
3,500
3,000
2,000
1,000
0
-500
3 - 0.475
2 - 0.460
0.452
5 - 2.327
4 - 2.214
min
0.00
0.50
1.00
1.50
2.00
2.50
3.00
3.50
4.00
4.91

Ac–Phe–(*R*)-γ4-Ala–(1*S*,2*S*)-ACBC–(*R*)-γ4-Phe–(1*S*,2*S*)-ACBC–Leu–OMe 7

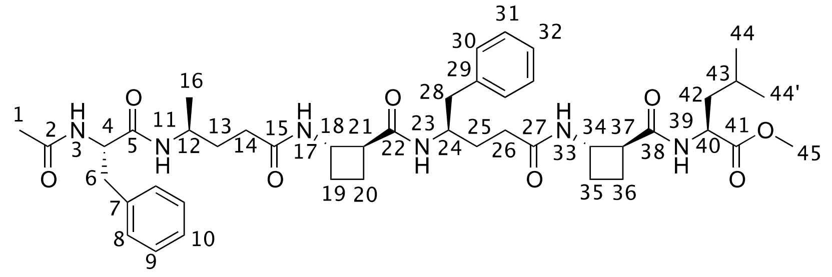

$C_{44}H_{62}N_6O_8$

M = 803.00 g.mol^{-1}

Following the general procedure B, **3** (40 mg, 0.05 mmol) was deprotected in 5 h to give the corresponding TFA salt, TFA·H_2N–Phe–(*R*)-γ^{4}Ala–(1*S*,2*S*)-ACBC–(*R*)-γ^{4}Trp–(1*S*,2*S*)-ACBC–Leu–OMe. Following the general procedure D, the acetylation was performed with the generated TFA salt (44 mg, 0.05 mmol), DIPEA (52 μL, 39 mg, 0.3 mmol) and acetic anhydride (7 μL, 8 mg, 0.075 mmol) in CH_2Cl_2 (2 mL) overnight to give peptide **7** (19 mg, 48%). The compound was purified using mass-directed preparative HPLC (gradient from 5/95 to 95/5 of 0.1% formic acid water/methanol) to afford **7** with purity higher than 95%. R_f 0.65 (CH_3OH / CH_2Cl_2 = 10 / 90); ^{1}H NMR (400 MHz, $CDCl_3/CD_3OD$:1/1) δ 0.88 (d, *J* = 6.4 Hz, 3H), 0.89 (d, *J* = 6.7 Hz, 3H), 1.09 (d, *J* = 6.6 Hz, 3H), 1.27 (s, 1H), 1.52-1.70 (m, 5H), 1.75-1.83 (m, 2H), 1.88-2.04 (m, 10H), 2.13-2.21 (m, 3H), 2.62-2.67 (m, 1H), 2.71-2.74 (m, 1H), 2.79-2.86 (m, 1H), 2.97-3.02 (m, 1H), 3.06-3.16 (m, 2H), 3.74 (s, 3H), 3.78-3.83 (m, 1H), 4.07-4.15 (m, 1H), 4.31-4.43 (m, 3H), 4.48-4.52 (m, 1H), 7.11-7.32 (m, 10H); ^{13}C NMR (100 MHz, $CDCl_3/CD_3OD$:1/1) δ 16.9, 17.1, 20.5, 20.8, 21.9, 22.5, 24.6, 24.7, 24.8, 29.4, 30.7, 31.5, 32.0, 32.1, 37.5, 39.8, 41.7 43.8, 44.1, 47.6, 48.7, 49.3, 51.1, 51.7, 55.9, 126.0, 126.7, 127.9, 128.1, 128.3, 128.3, 128.9, 129.2, 136.8, 138.2, 171.6, 172.4, 172.9, 173.6, 173.8, 174.3; HRMS (ESI): $[M+Na]^+$, 825.4526 found, $C_{44}H_{62}N_6NaO_8$ calcd 825.4527.

LCMS:

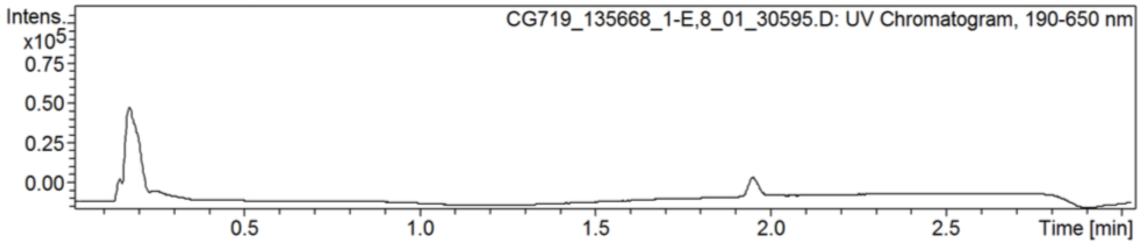

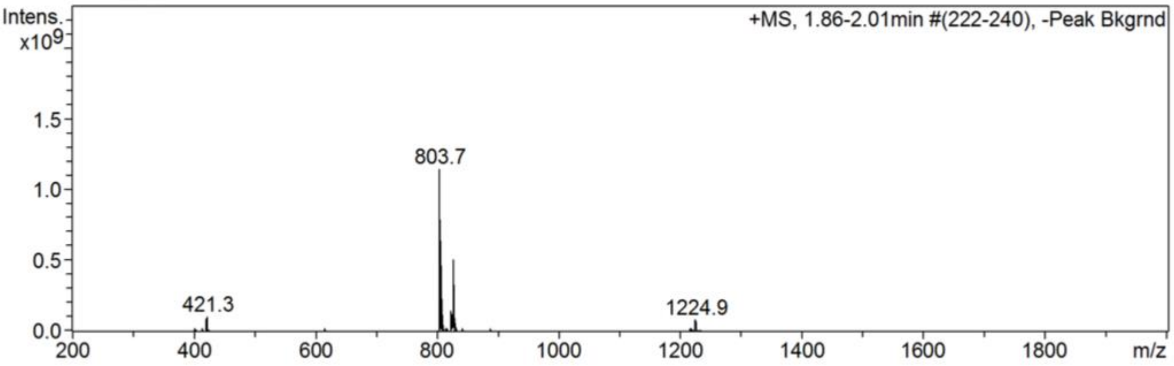
Intens.
x10^9
+MS, 1.86-2.01min #(222-240), -Peak Bkgrnd
803.7
421.3
1224.9
m/z

HPLC:

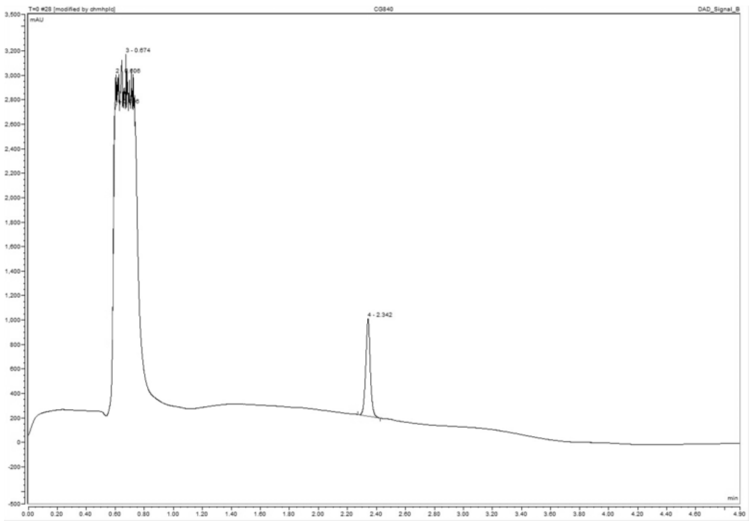
DAD_Signal_B
mAU
3 - 0.674
4 - 2.342
min

Ac–Phe–(*R*)-γ⁴-Ala–(1*S*,2*S*)-ACBC–(*R*)-γ⁴-Phe–(1*S*,2*S*)-ACBC–(*R*)-γ⁴-Leu–OMe 8

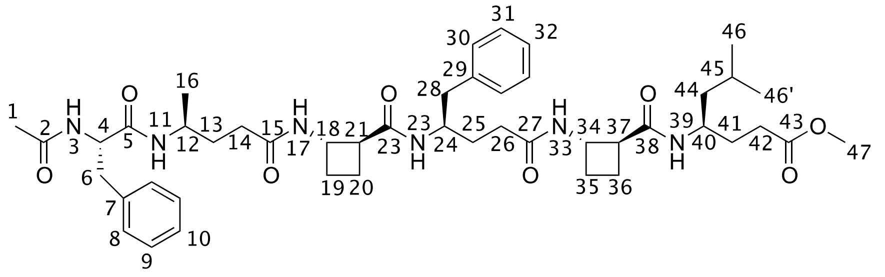

$C_{46}H_{66}N_6O_8$

M = 831.05 g.mol^{-1}

Following the general procedure B, *tert*-butoxycarbonyl group of **4** (45 mg, 0.05 mmol) was deprotected in 5 h to give the corresponding TFA salt, TFA·H_2N–Phe–(*R*)-γ^{4}Ala–(1*S*,2*S*)-ACBC–(*R*)-γ^{4}Trp–(1*S*,2*S*)-ACBC–Leu–OMe. Following the general procedure D, the acetylation was performed with the generated TFA salt (44 mg, 0.05 mmol), DIPEA (52 μL, 39 mg, 0.3 mmol) and acetic anhydride (7 μL, 8 mg, 0.075 mmol) in CH_2Cl_2 (2 mL) overnight to give peptide **8** (15 mg, 37%). The compound was purified using mass-directed preparative HPLC (gradient from 5/95 to 95/5 of 0.1% formic acid water/methanol) to afford **8** with purity higher than 95%. R_f 0.61 (CH_3OH / CH_2Cl_2 = 10 / 90); ^{1}H NMR (400 MHz, $CDCl_3/CD_3OD$:1/1) δ 0.76-0.78 (m, 6H), 0.98 (d, *J* = 6.8 Hz, 3H), 1.09-1.14 (m, 1H), 1.24-1.29 (m, 2H), 1.35-1.49 (m, 4H), 1.59-1.64 (m, 2H), 1.70-1.90 (m, 9H), 2.03-2.15 (m, 4H), 2.31-2.38 (m, 2H), 2.60-2.72 (m, 3H), 2.88-2.95 (m, 2H), 3.04 (dd, *J* = 13.8 Hz, *J* = 6.2 Hz, 1H), 3.60 (s, 3H), 3.69 (bs, 1H), 3.83-3.90 (m, 2H), 4.03 (bs, 1H), 4.19-4.24 (m, 1H), 4.26-4.30 (m, 1H), 4.42-4.44 (m, 1H), 7.07-7.31 (m, 10H); ^{13}C NMR (100 MHz, $CDCl_3/CD_3OD$:1/1) δ 16.7, 17.8, 20.9, 21.9, 22.4, 22.8, 24.2, 24.8, 24.8, 25.1, 29.5, 30.5, 30.7, 31.0, 31.5, 32.1, 37.3, 41.5 43.6, 43.7, 46.6, 47.6, 47.7, 48.9, 49.4, 49.5, 51.4, 55.5, 126.1, 127.0, 128.0, 128.6, 128.9, 129.1, 136.3, 138.1, 171.1, 172.5, 172.7, 173.5, 173.7, 174.6; HRMS (ESI): [M+Na]$^+$, 853.4858 found, $C_{46}H_{66}N_6NaO_8$ calcd 853.4840.

LCMS:

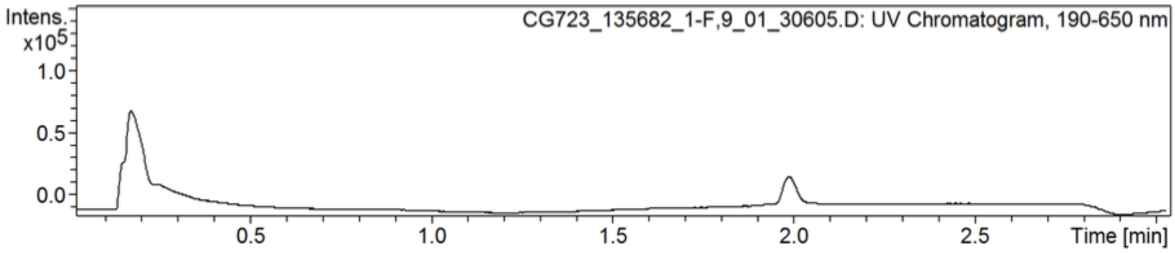

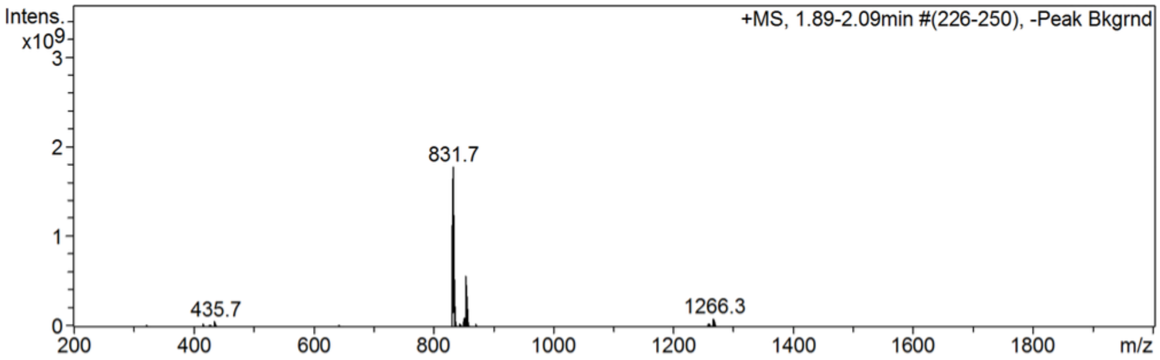
Intens.
x10^9
+MS, 1.89-2.09min #(226-250), -Peak Bkgrnd
831.7
435.7
1266.3
m/z

HPLC:

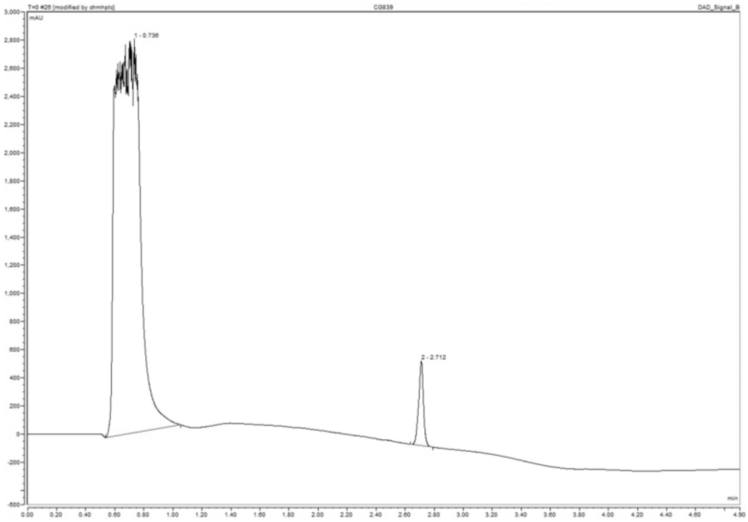
mAU
1 - 0.736
2 - 2.712
min

II. NMR spectroscopic analysis of peptides 1-8 and I-VIII

1. ^{1}H and ^{13}C NMR spectra

Boc–(*R*)-γ⁴-Ala–(1*S*,2*S*)-ACBC–OMe I

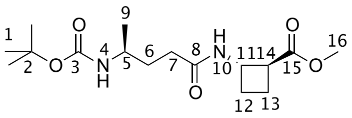

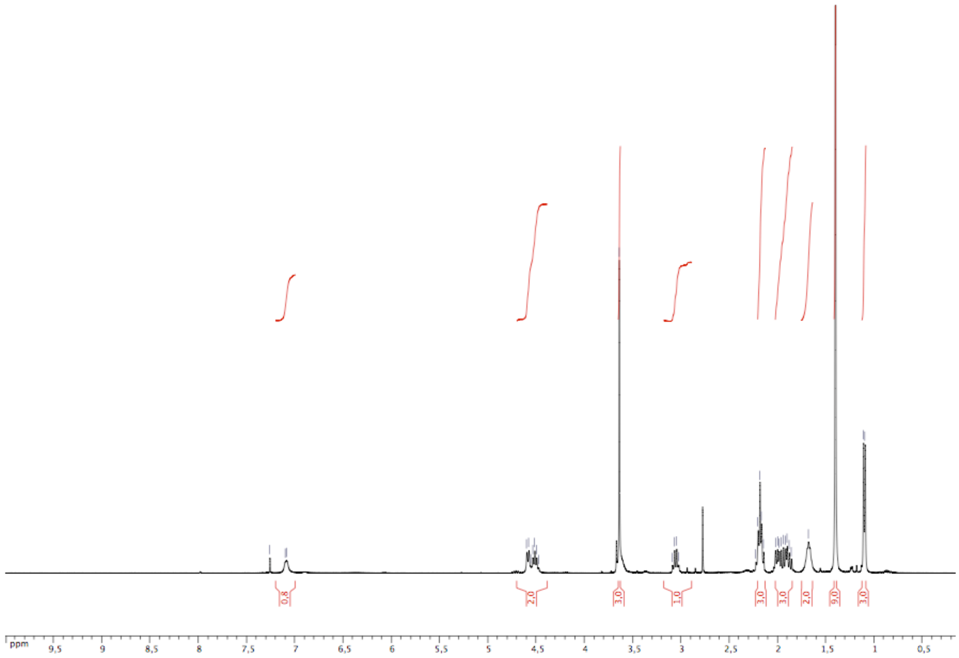

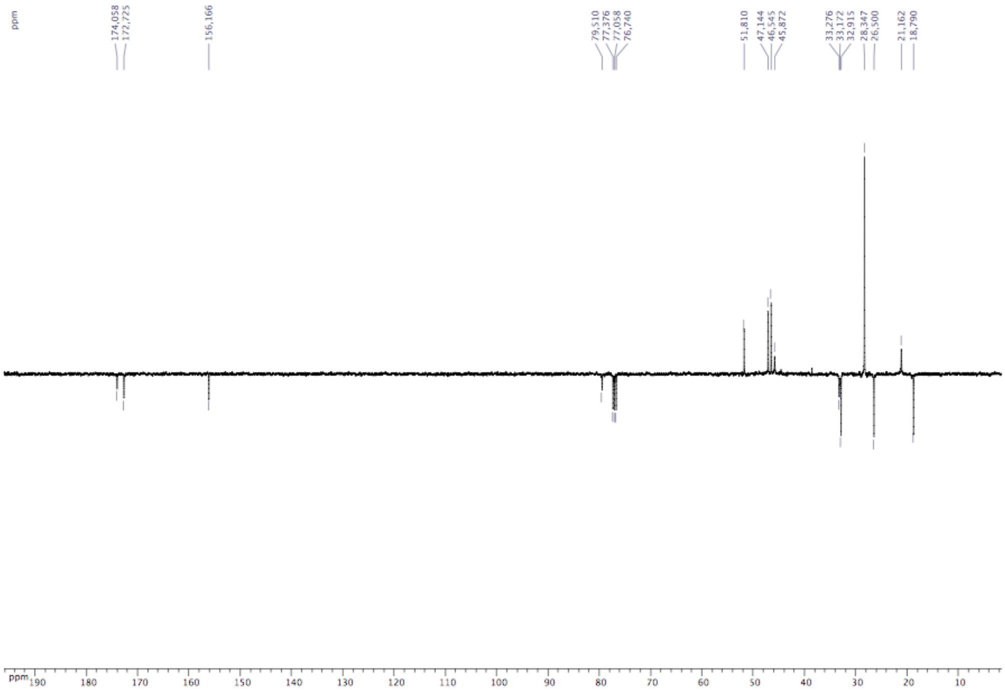

Boc–Phe–(*R*)-γ⁴-Ala–(1*S*,2*S*)-ACBC–OMe II

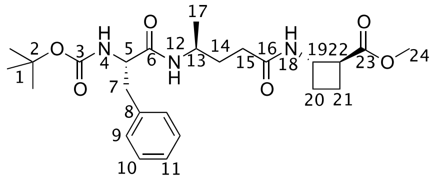

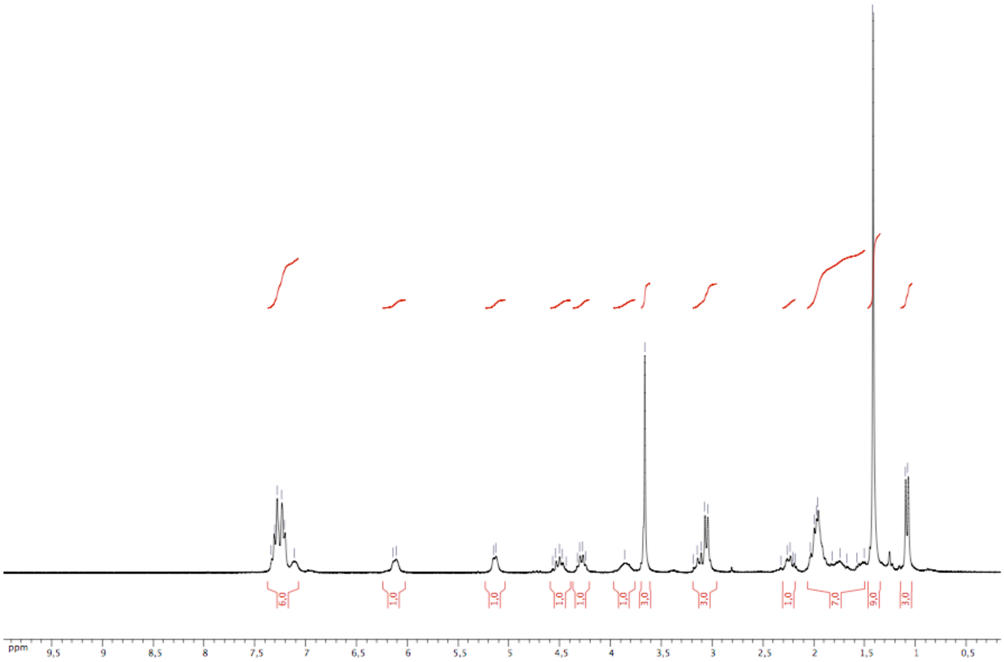

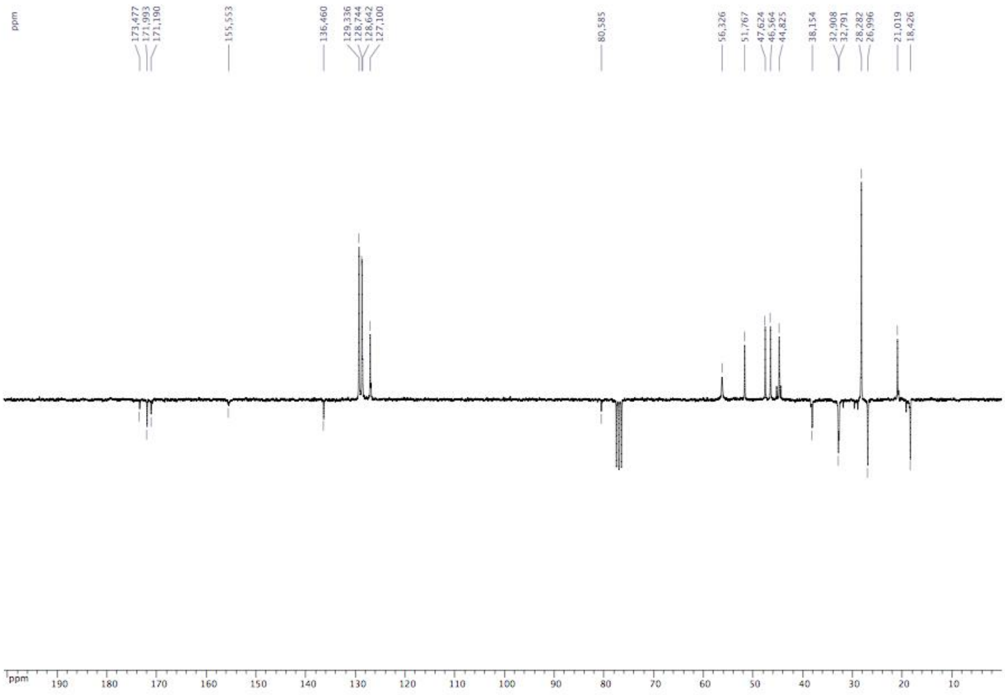

Boc–(*R*)-γ⁴Trp–(1*S*,2*S*)-ACBC–OMe III

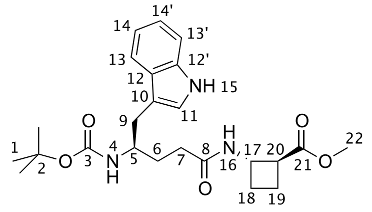

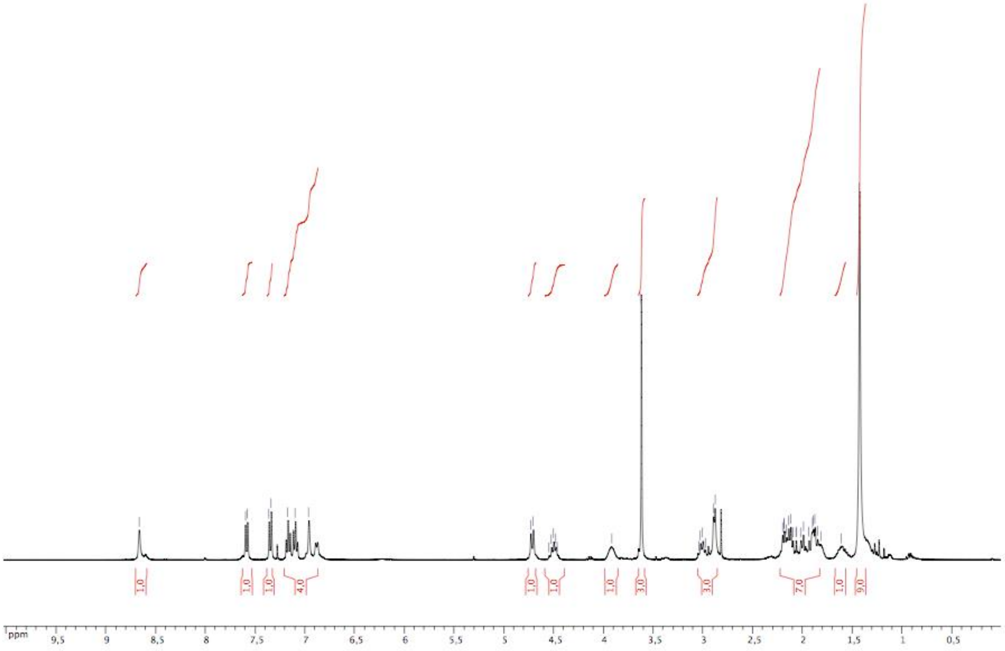

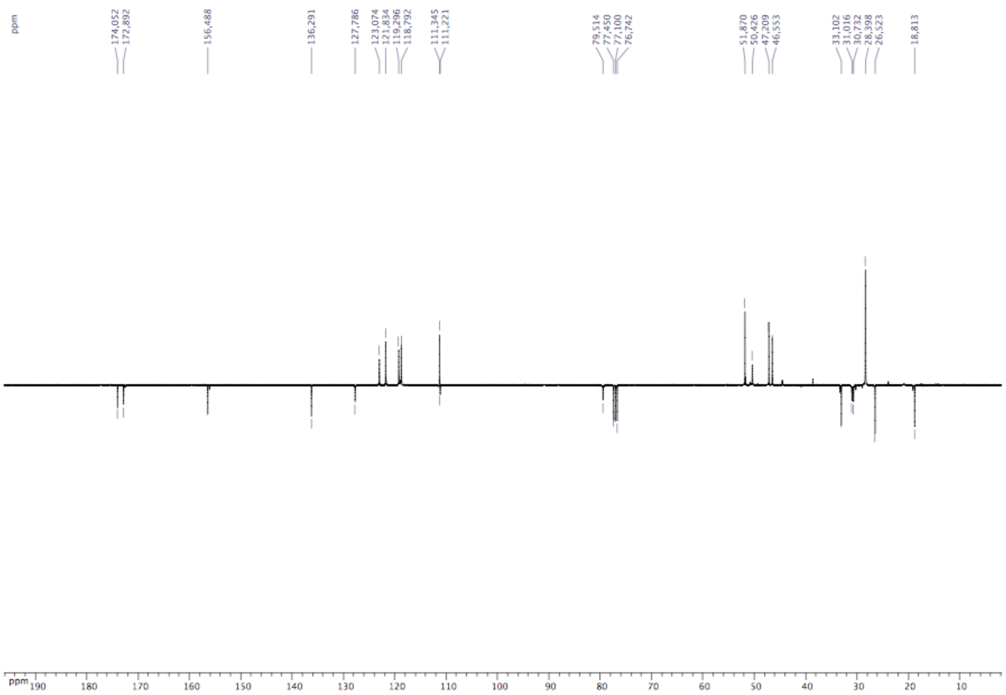

Boc–(*R*)-γ⁴-Trp–(1*S*,2*S*)-ACBC–Leu–OMe IV

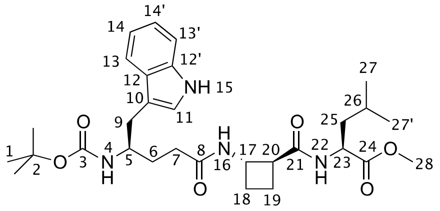

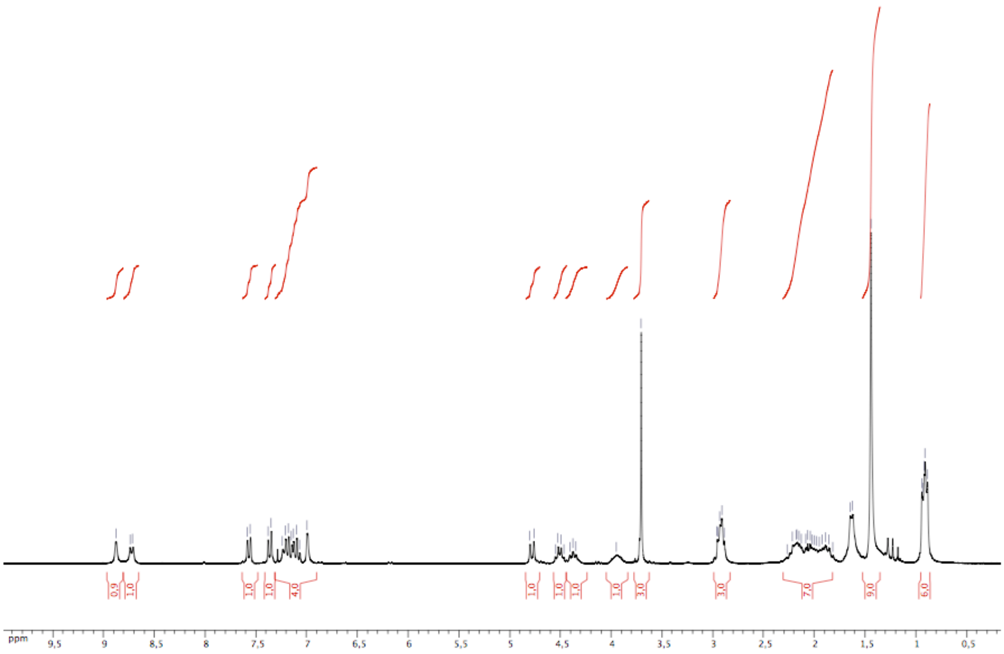

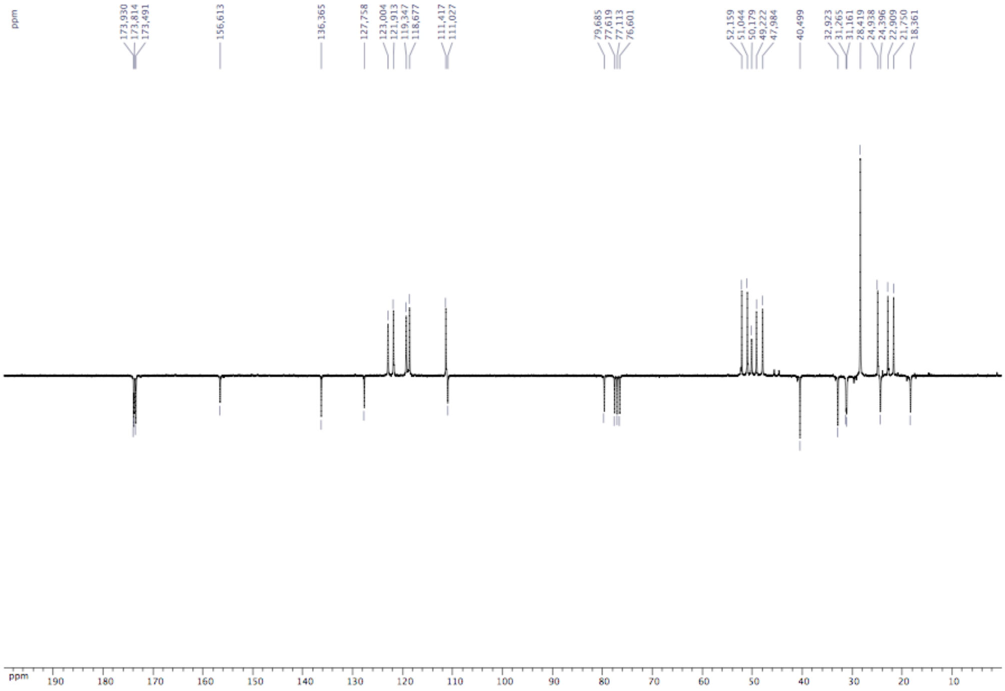

Boc–(*R*)-γ4-Trp–(1*S*,2*S*)-ACBC–(*R*)-γ4-Leu–OMe V

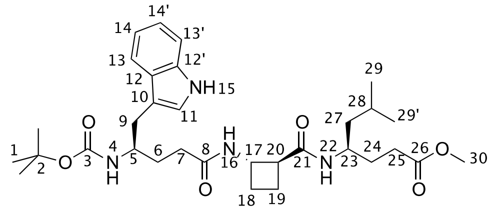

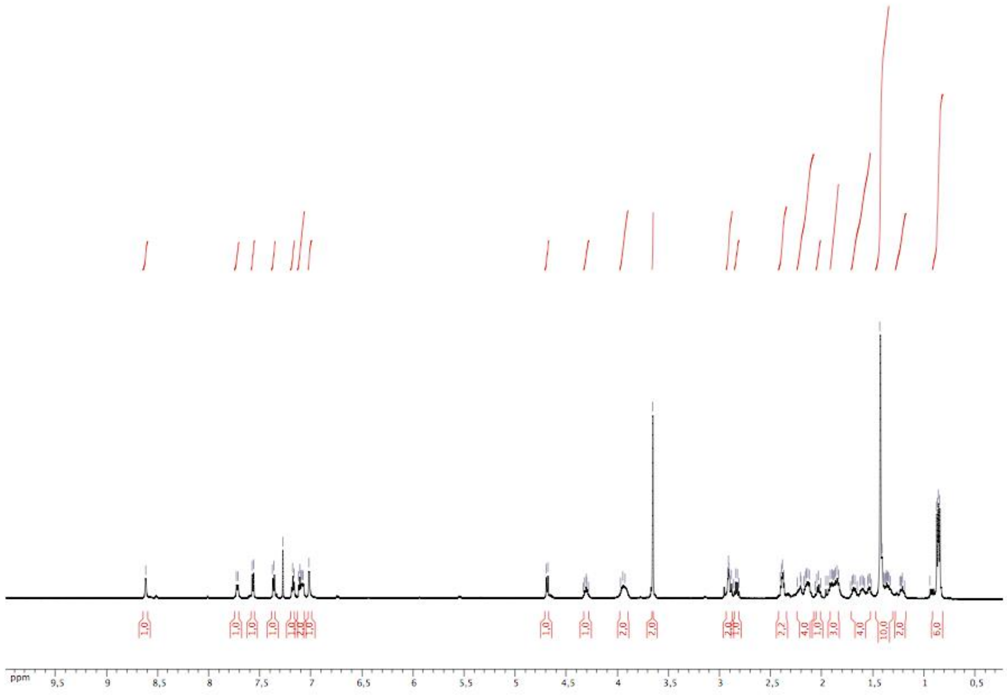

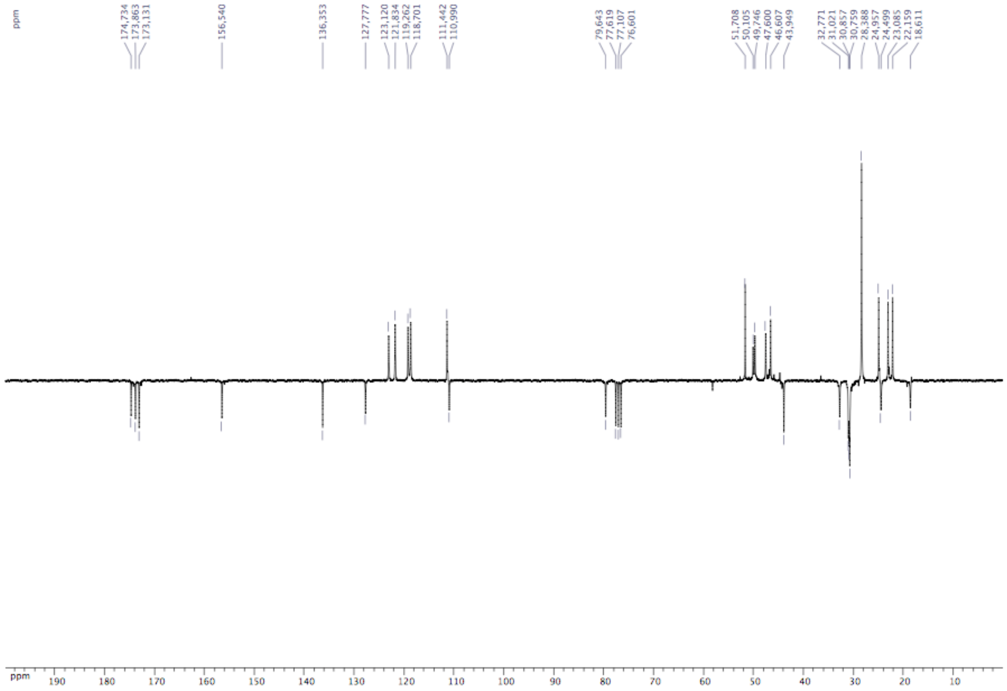

Boc–Phe–(*R*)-γ⁴-Ala–(1*S*,2*S*)-ACBC–(*R*)-γ⁴-Trp–(1*S*,2*S*)-ACBC–Leu–OMe 1

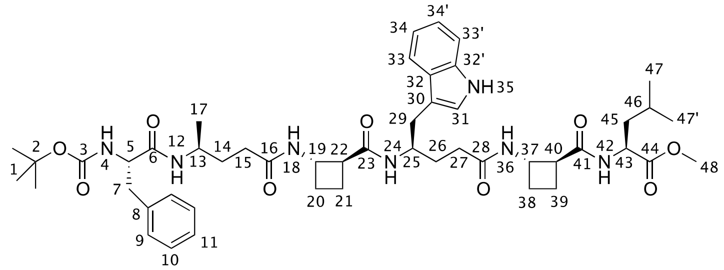

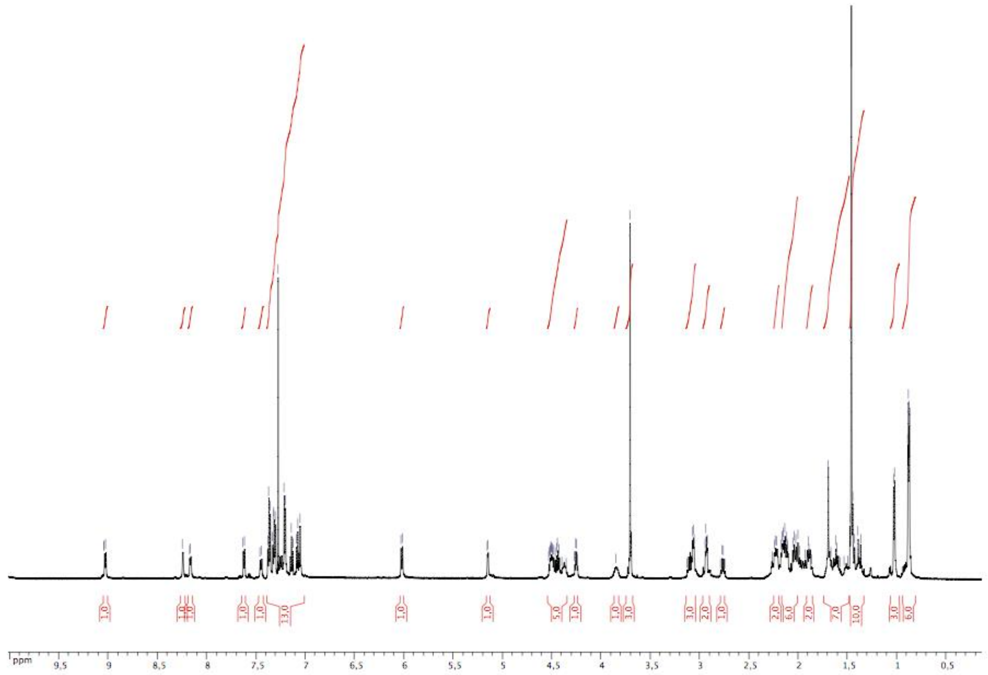

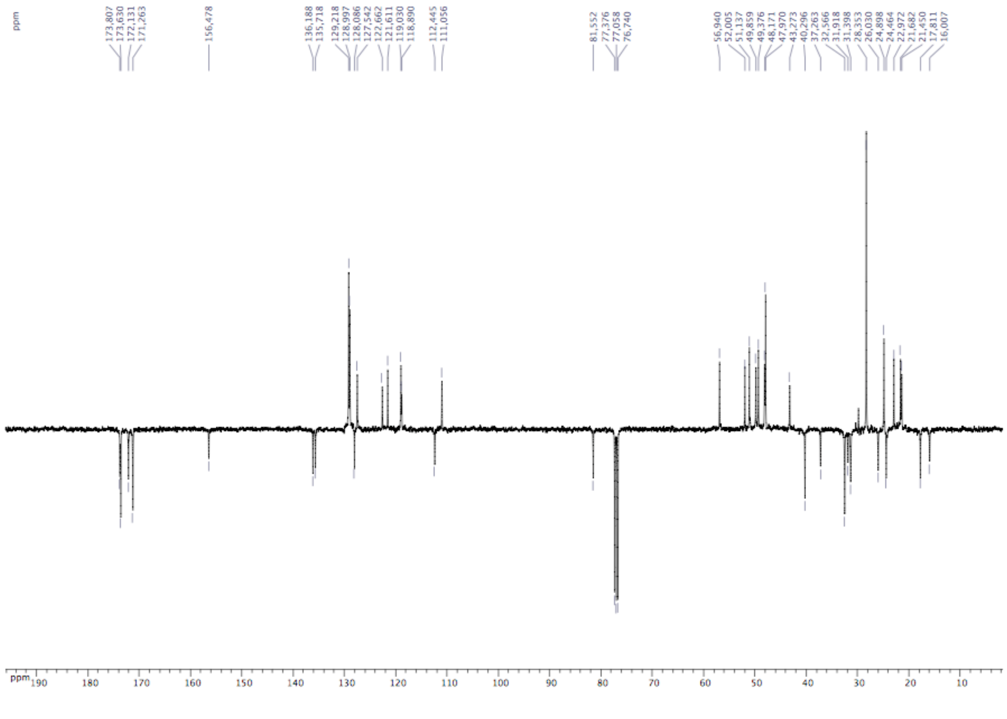

Boc–Phe–(*R*)-γ⁴-Ala–(1*S*,2*S*)-ACBC–(*R*)-γ⁴-Trp–(1*S*,2*S*)-ACBC–(*R*)-γ⁴-Leu–OMe 2

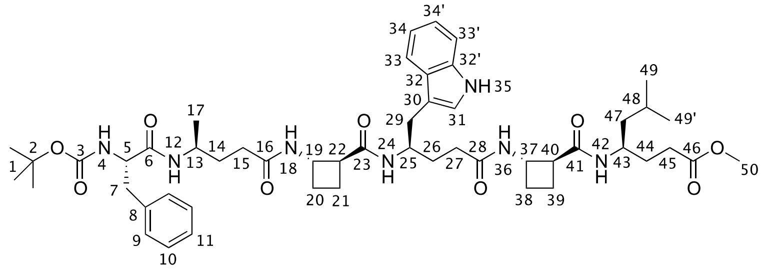

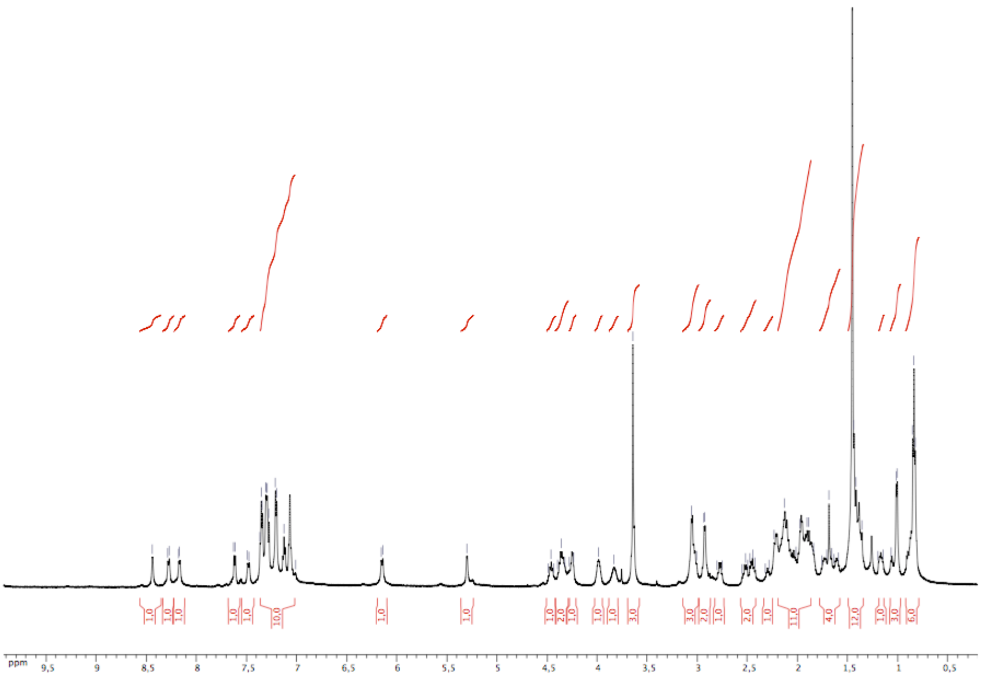

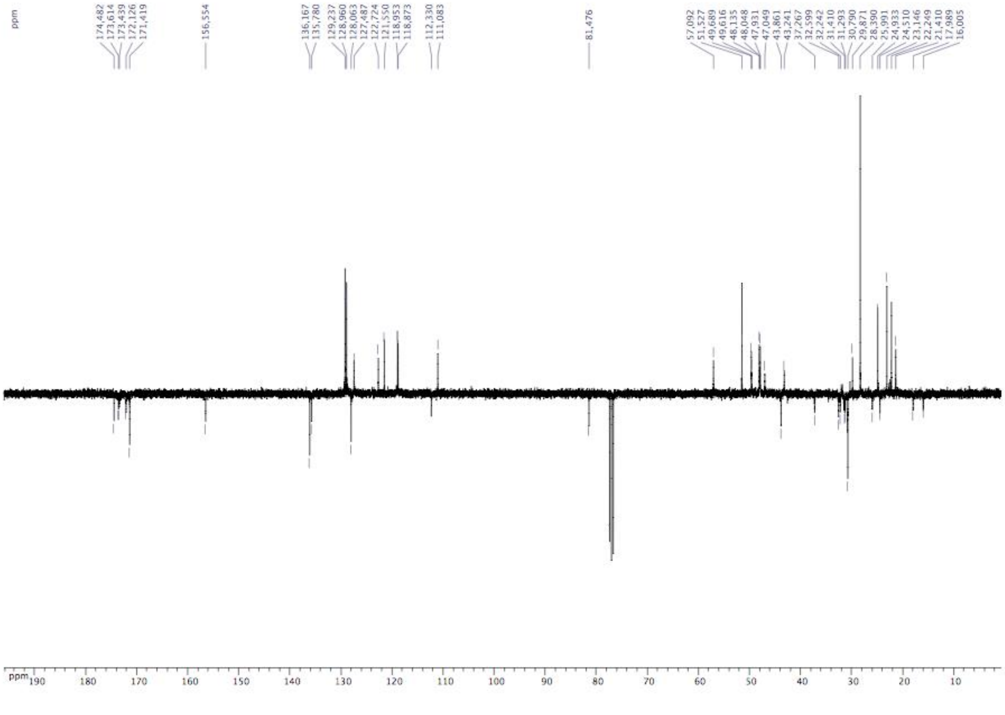

Boc–(*R*)-γ⁴-Phe–(1*S*,2*S*)-ACBC–Leu–OMe VI

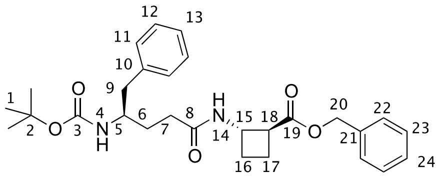

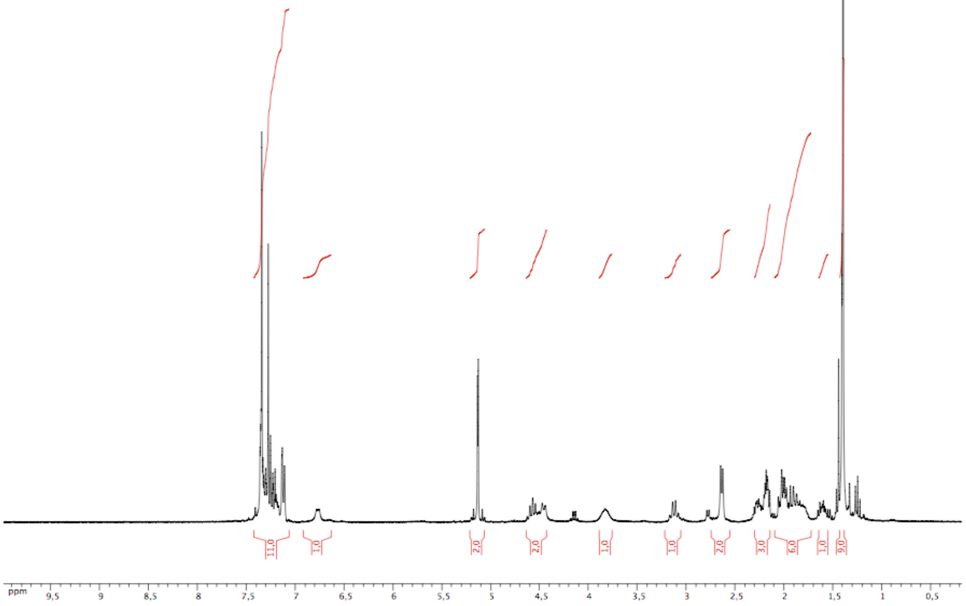

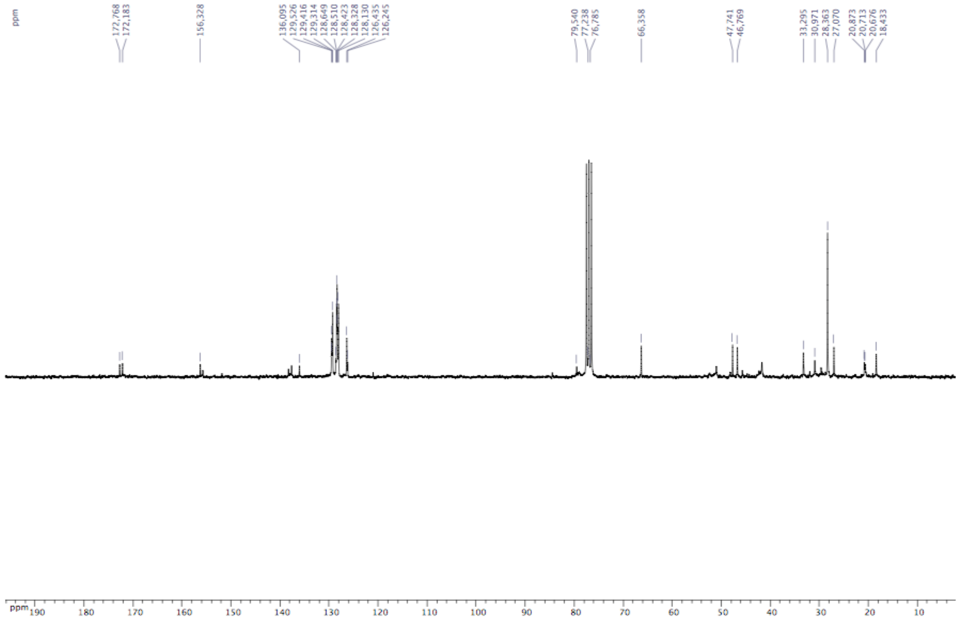

Boc–(*R*)-γ4-Phe–(1*S*,2*S*)-ACBC–Leu–OMe VII

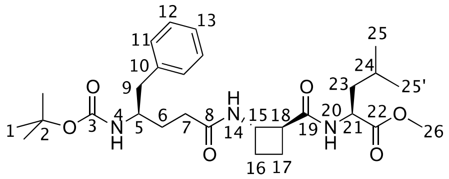

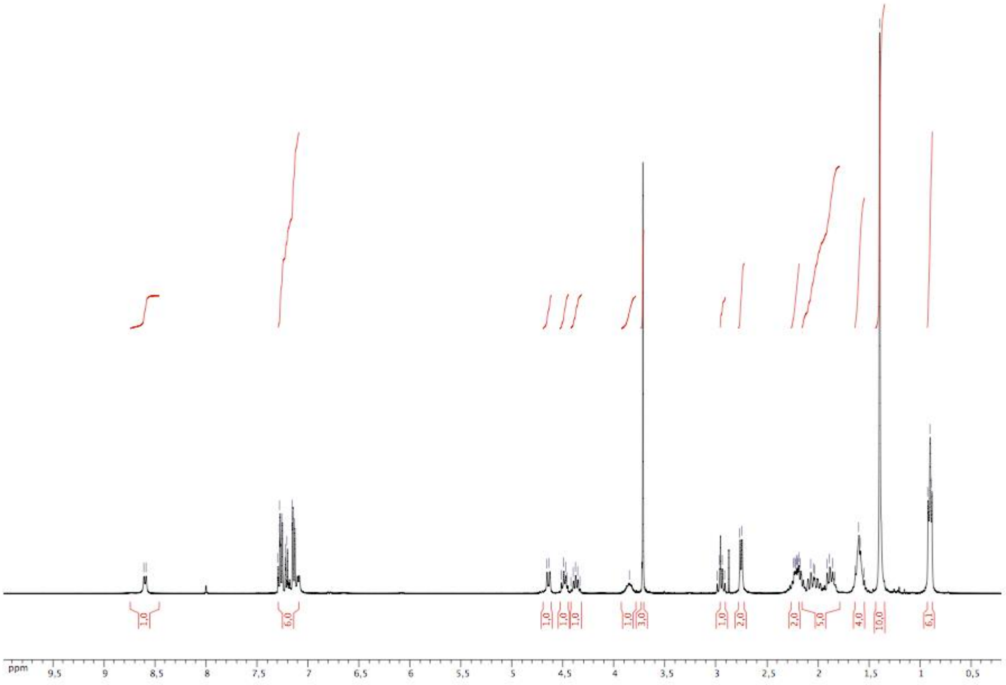

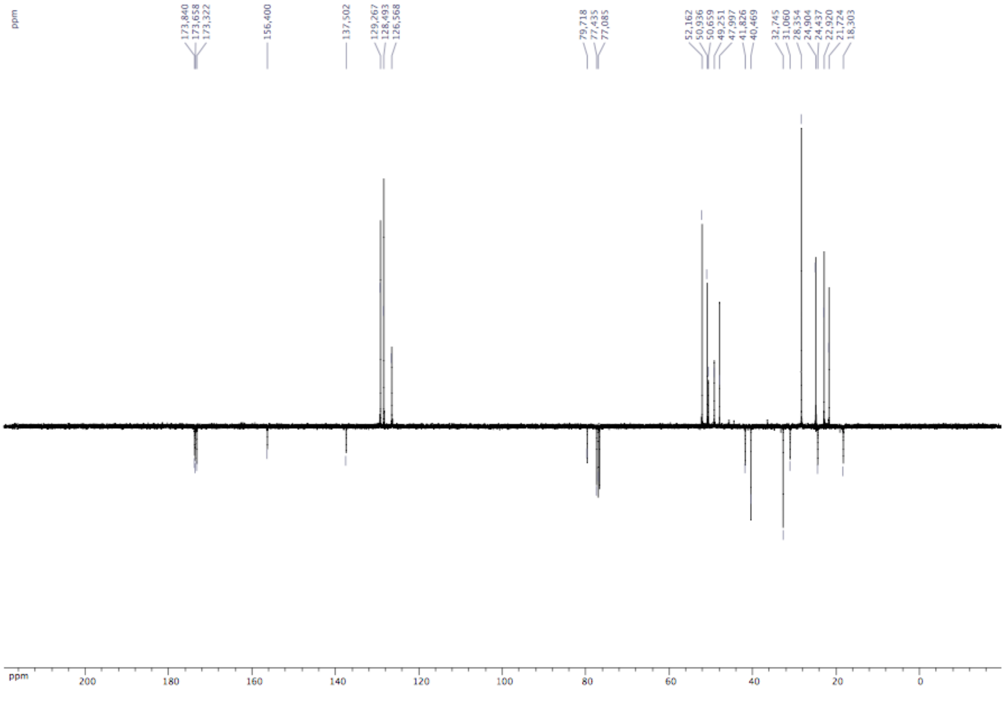

Boc–(*R*)-γ⁴-Phe–(1*S*,2*S*)-ACBC–(*R*)-γ⁴-Leu–OMe VIII

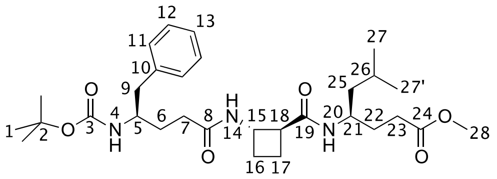

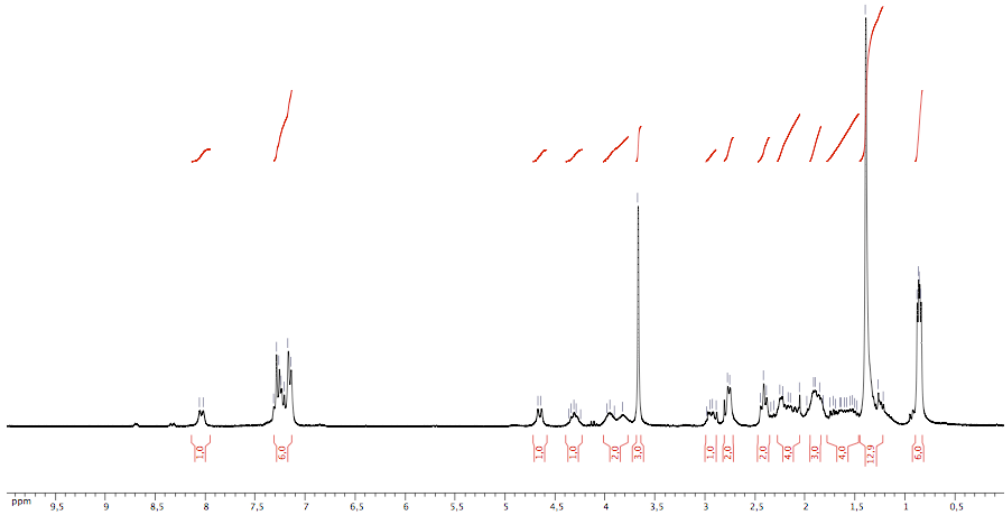

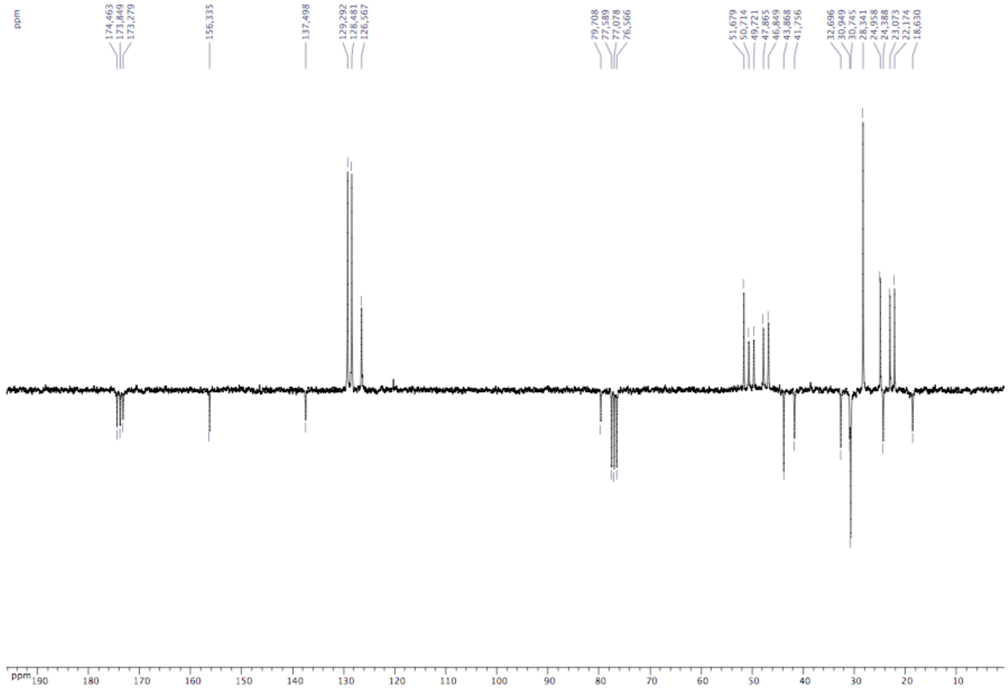

Boc–Phe–(*R*)-γ⁴-Ala–(1*S*,2*S*)-ACBC–(*R*)-γ⁴-Phe–(1*S*,2*S*)-ACBC–Leu–OMe 3

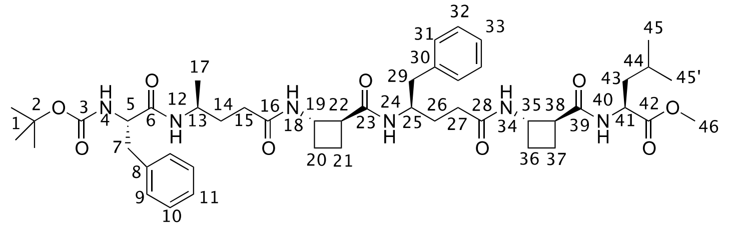

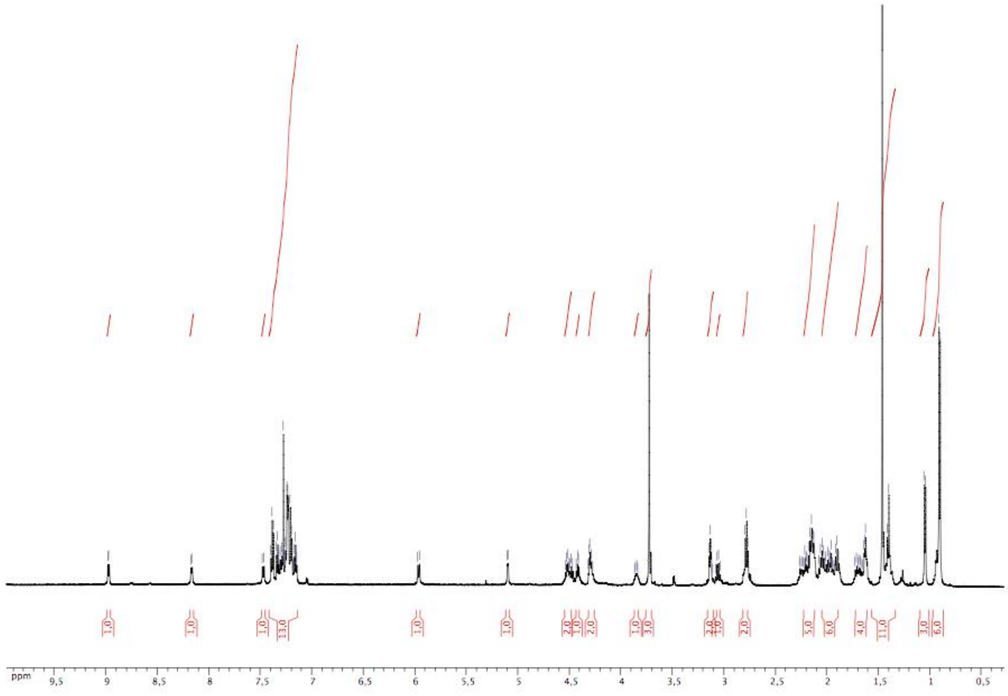

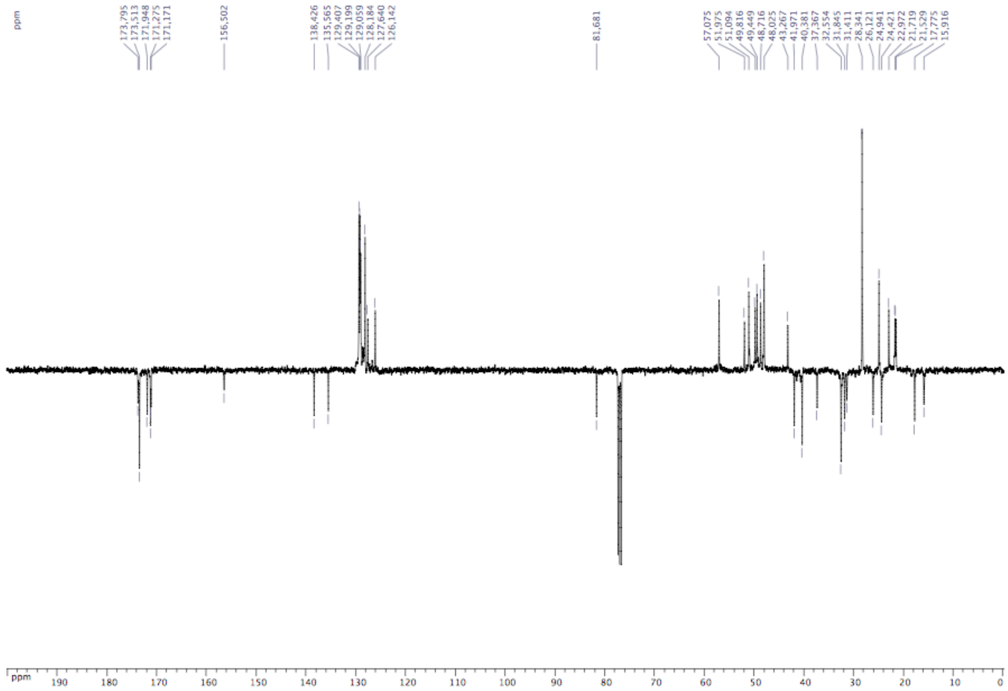

Boc–Phe–(*R*)-γ⁴-Ala–(1*S*,2*S*)-ACBC–(*R*)-γ⁴-Phe–(1*S*,2*S*)-ACBC–(*R*)-γ⁴-Leu–OMe 4

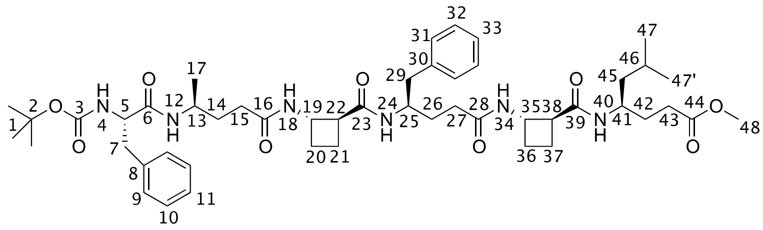

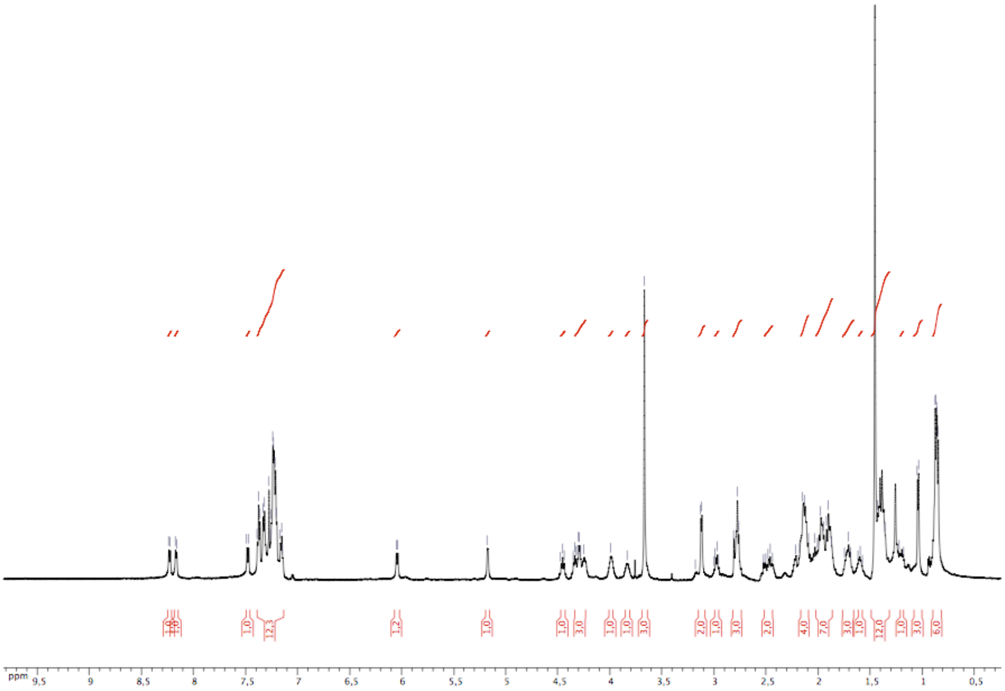

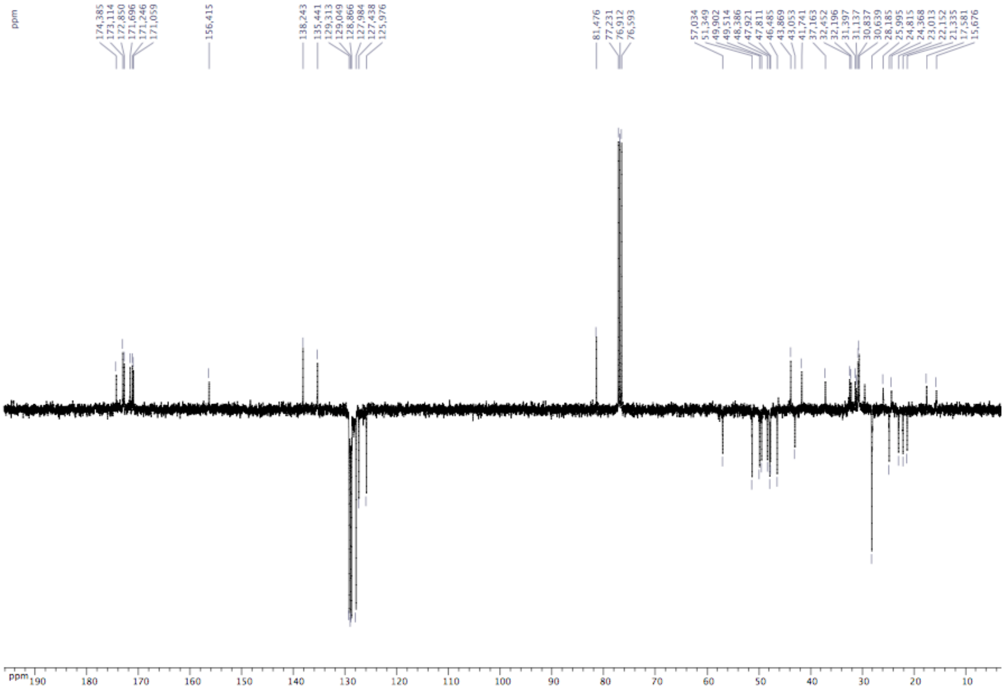

Ac–Phe–(*R*)-γ⁴-Ala–(1*S*,2*S*)-ACBC–(*R*)-γ⁴-Trp–(1*S*,2*S*)-ACBC–Leu–OMe 5

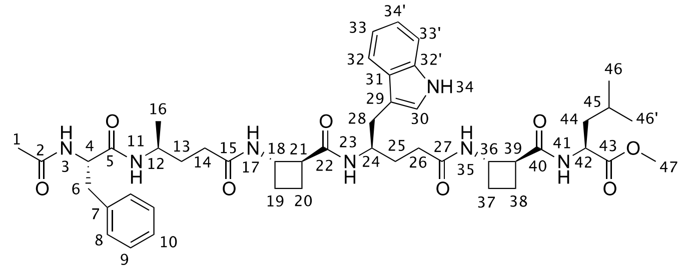

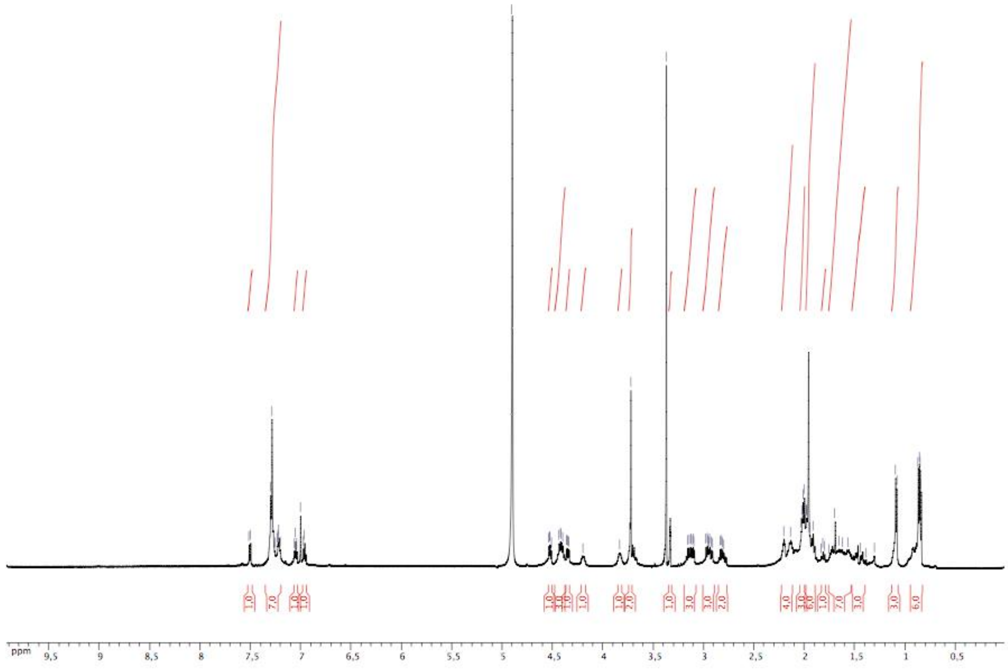

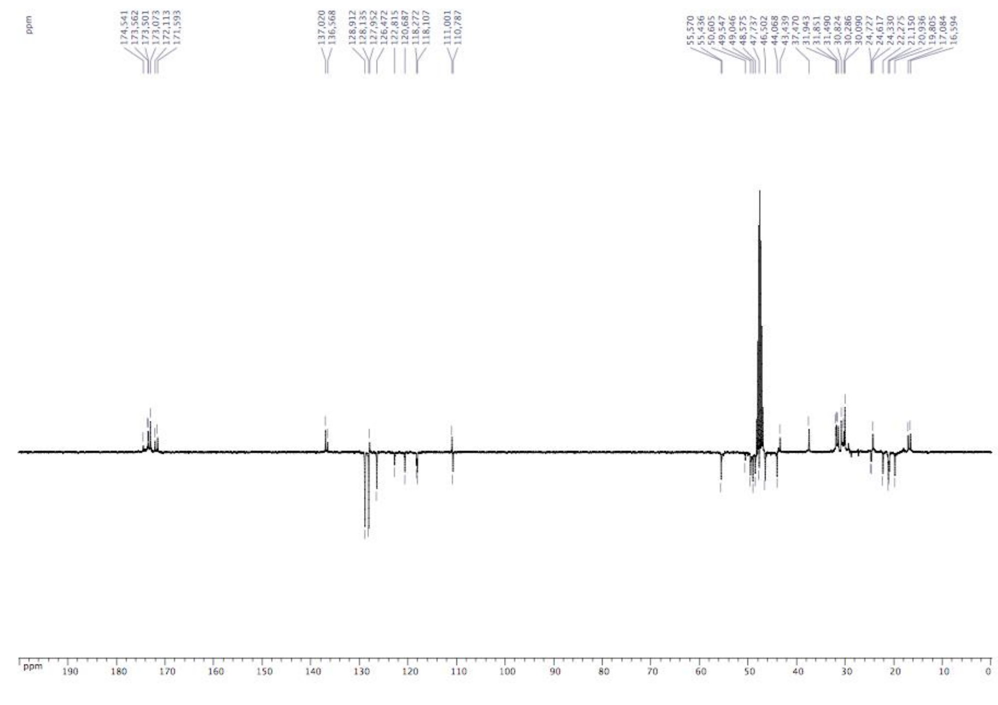

Ac–Phe–(*R*)-γ⁴-Ala–(1*S*,2*S*)-ACBC–(*R*)-γ⁴-Trp–(1*S*,2*S*)-ACBC–(*R*)-γ⁴-Leu–OMe 6

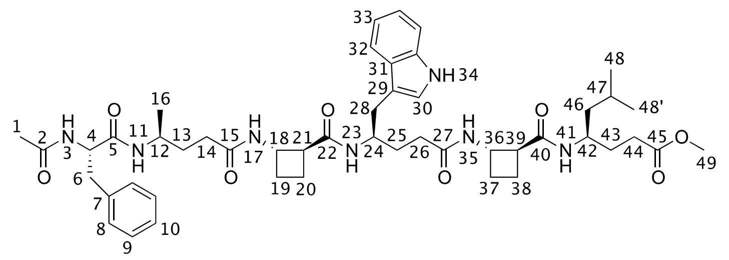

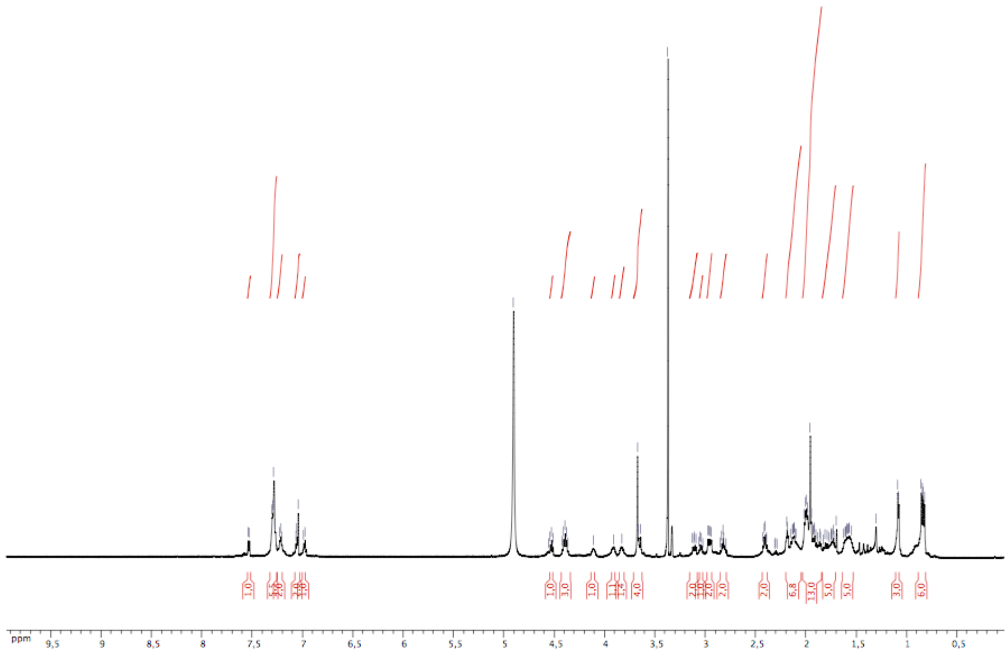

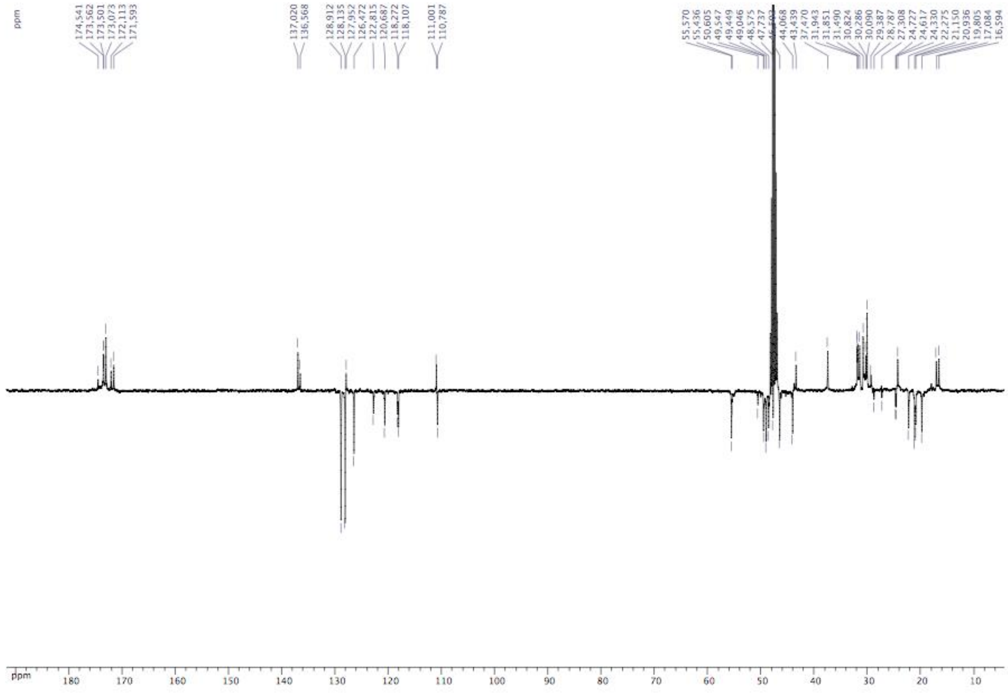

Ac–Phe–(*R*)-γ⁴-Ala–(1*S*,2*S*)-ACBC–(*R*)-γ⁴-Phe–(1*S*,2*S*)-ACBC–Leu–OMe 7

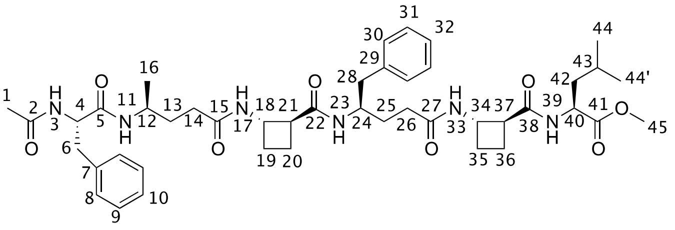

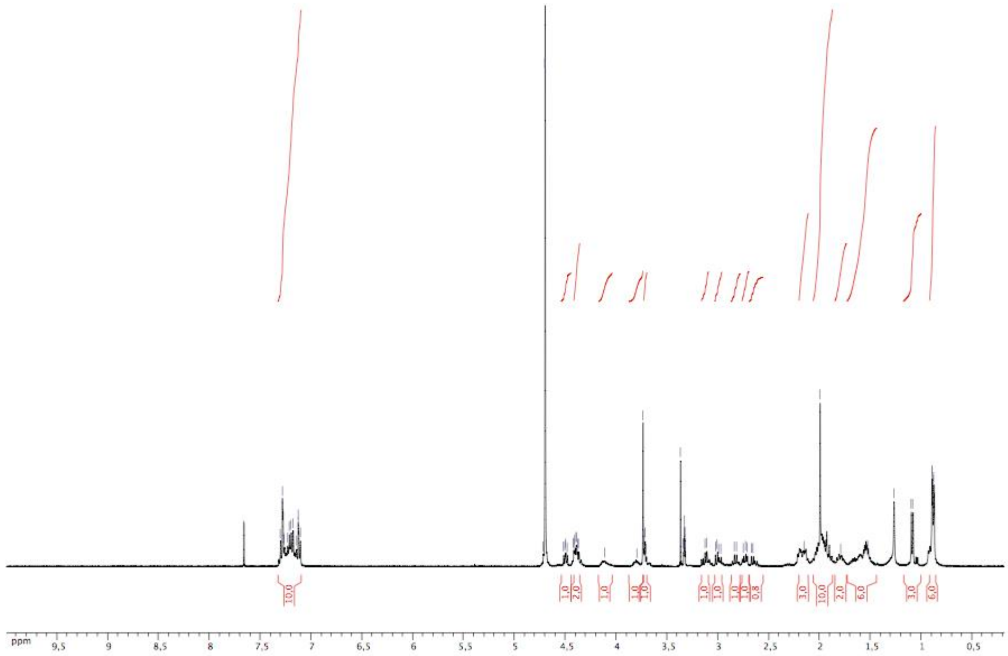

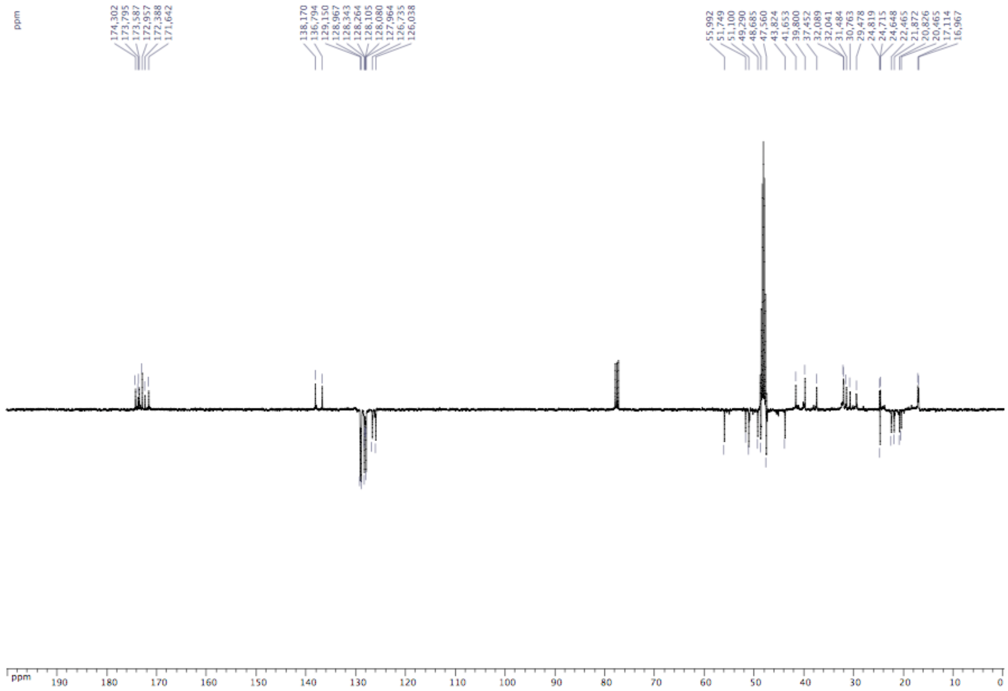

Ac–Phe–(*R*)-γ⁴-Ala–(1*S*,2*S*)-ACBC–(*R*)-γ⁴-Phe–(1*S*,2*S*)-ACBC–(*R*)-γ⁴-Leu–OMe 8

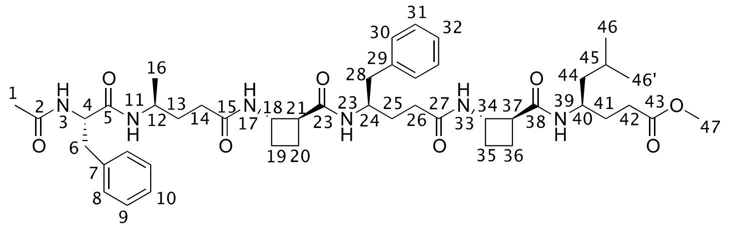

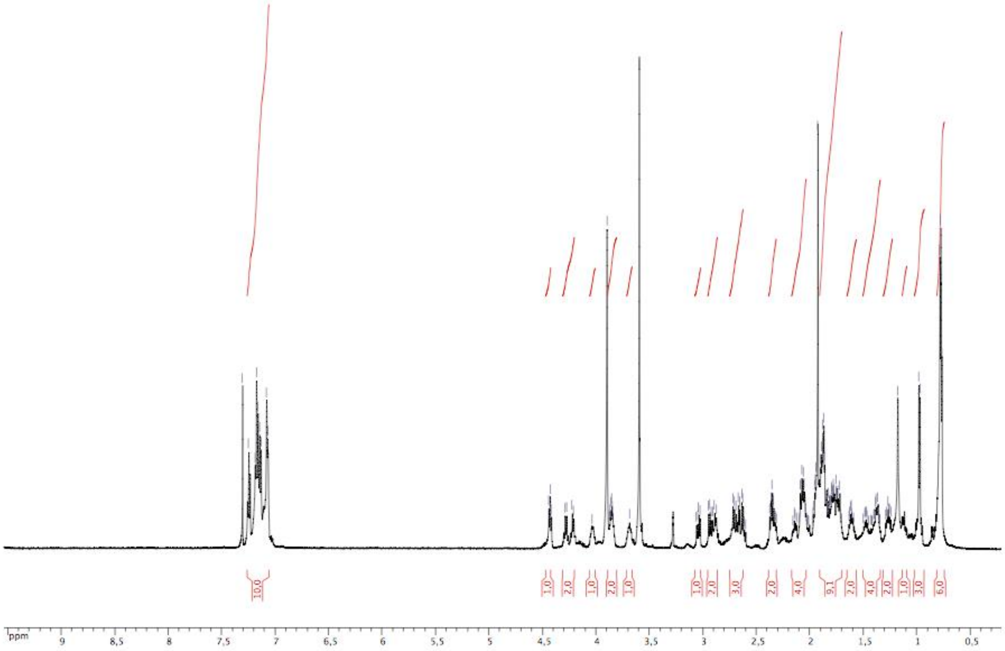

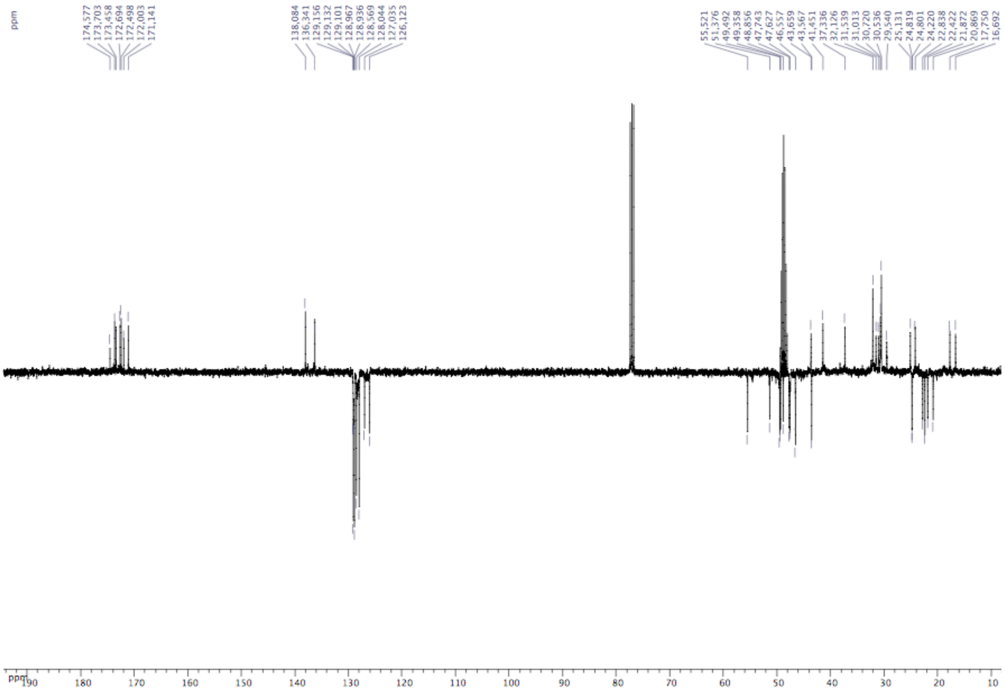

2. *DMSO-d_6 titrations*

^1H Spectra were recorded at 300 K on a Bruker 600 MHz spectrometer. Samples were dissolved in $CDCl_3$ (600 μL) to give solutions of concentration 10 mM. Aliquots of DMSO-d_6 (20 μL, 40 μL, 60 μL, 80 μL and 100 μL) were added successively to the NMR tube followed, after each addition, by rapid agitation then re-recording of the ^1H spectra.

Boc–Phe–(*R*)-γ⁴-Ala–(1*S*,2*S*)-ACBC–(*R*)-γ⁴-Trp–(1*S*,2*S*)-ACBC–Leu–OMe 1

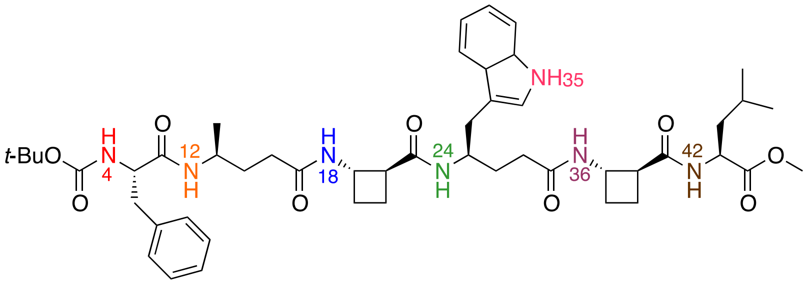

DMSO-d_6 (%, v/v)							
NH	**0%**	**3%**	**10%**	**20%**	**33%**	**50%**	**Δδ**
NH(4)	5.12	5.48	5.84	6.11	6.32	6.51	1.39
NH(12)	6.02	6.47	6.93	-	-	7.60	1.58
NH(18)	7.24	-	-	7.43	7.57	7.70	0.52
NH(24)	7.48	7.52	7.50	7.64	7.72	7.88	0.40
NH(35)	8.24	8.87	9.53	9.98	10.24	10.43	2.19
NH(36)	8.20	8.23	8.27	8.31	8.31	8.32	0.12
NH(42)	9.03	9.05	9.05	9.05	9.05	9.00	-0.03

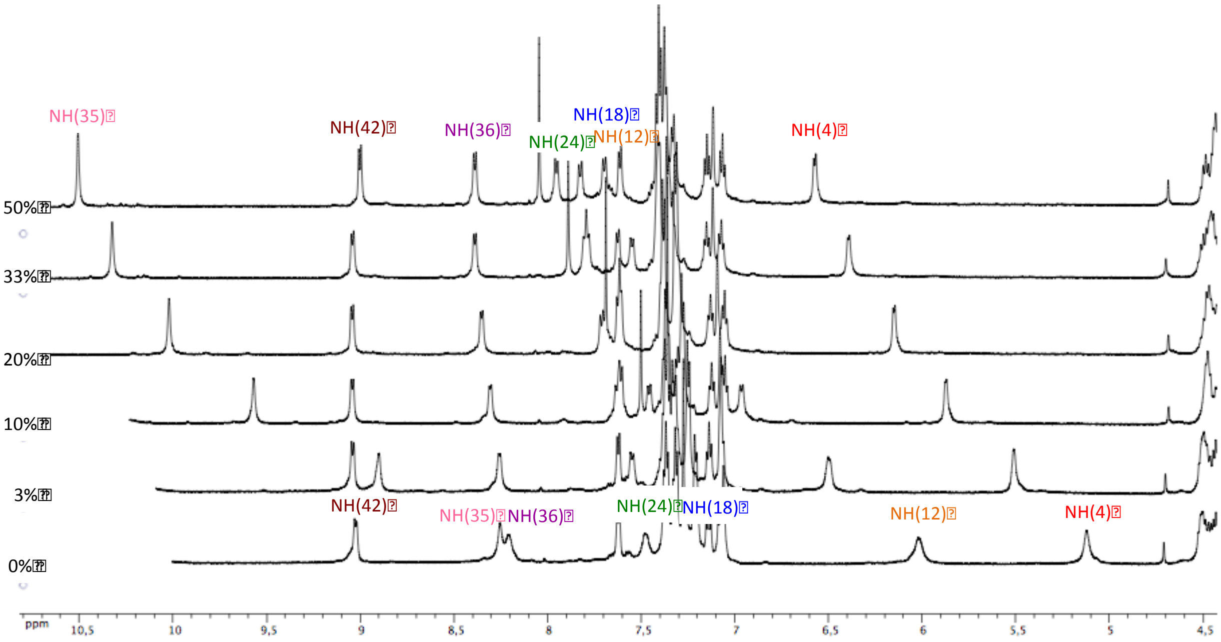

Boc–Phe–(*R*)-γ⁴-Ala–(1*S*,2*S*)-ACBC–(*R*)-γ⁴-Trp–(1*S*,2*S*)-ACBC–(*R*)-γ⁴-Leu–OMe 2

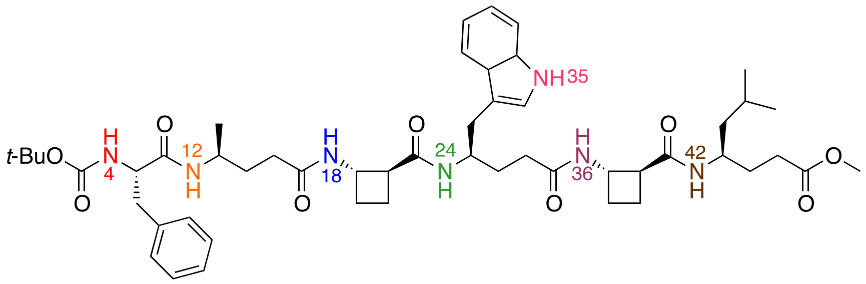

DMSO-d_6 (%, v/v)							
NH	**0%**	**3%**	**10%**	**20%**	**33%**	**50%**	**Δδ**
NH(4)	5.30	5.58	5.88	6.14	6.36	6.54	1.24
NH(12)	6.14	6.54	7.08	-	7.51	7.67	1.53
NH(18)	7.36	-	7.44	7.60	7.74	7.80	0.44
NH(24)	7.48	7.54	7.59	7.69	7.74	7.91	0.43
NH(35)	8.44	8.95	9.52	9.96	10.23	10.44	2.00
NH(36)	8.16	8.21	8.25	8.30	8.31	8.33	0.17
NH(42)	8.27	8.27	8.26	8.23	8.22	8.19	–0.08

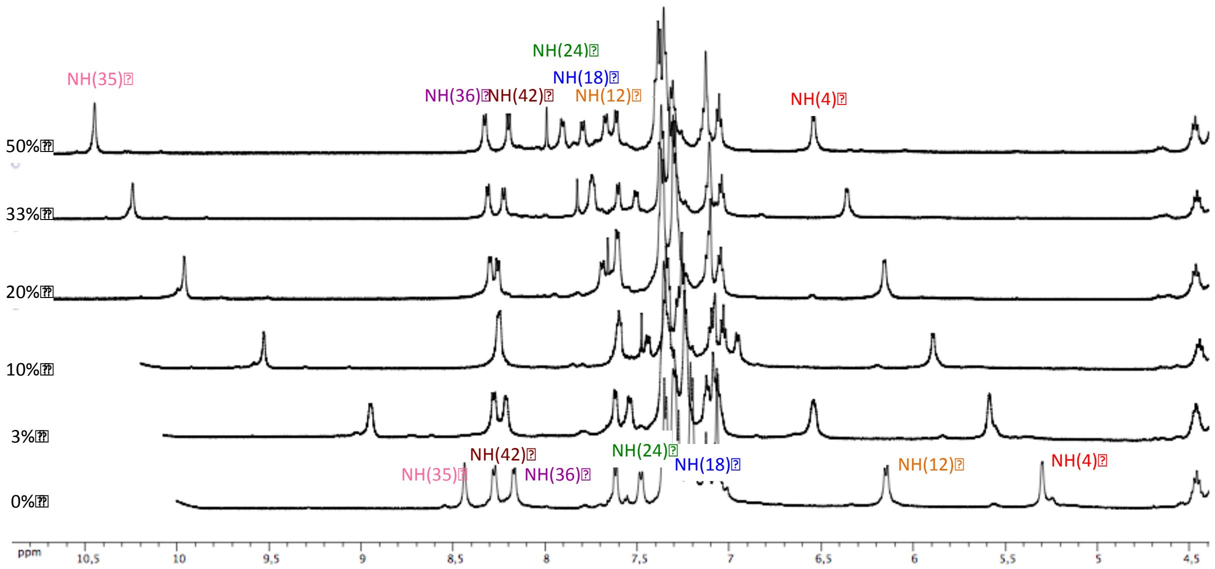

Boc–Phe–(*R*)-γ⁴-Ala–(1*S*,2*S*)-ACBC–(*R*)-γ⁴-Phe–(1*S*,2*S*)-ACBC–Leu–OMe 3

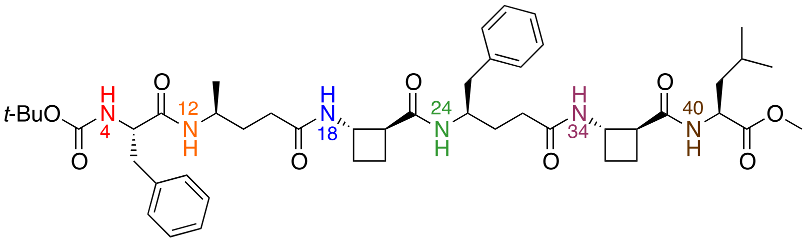

DMSO-d_6 (%, v/v)							
NH	**0%**	**3%**	**10%**	**20%**	**33%**	**50%**	**Δδ**
NH(4)	5.11	5.46	5.89	6.15	6.38	6.94	1.83
NH(12)	5.99	6.41	6.96	-	7.52	7.67	1.68
NH(18)	7.29	-	7.46	7.60	7.74	7.88	0.59
NH(24)	7.45	7.56	7.64	7.72	7.76	7.82	0.37
NH(34)	8.20	8.20	8.27	8.32	8.34	8.38	0.18
NH(40)	8.97	8.98	8.98	8.99	8.98	8.97	0.00

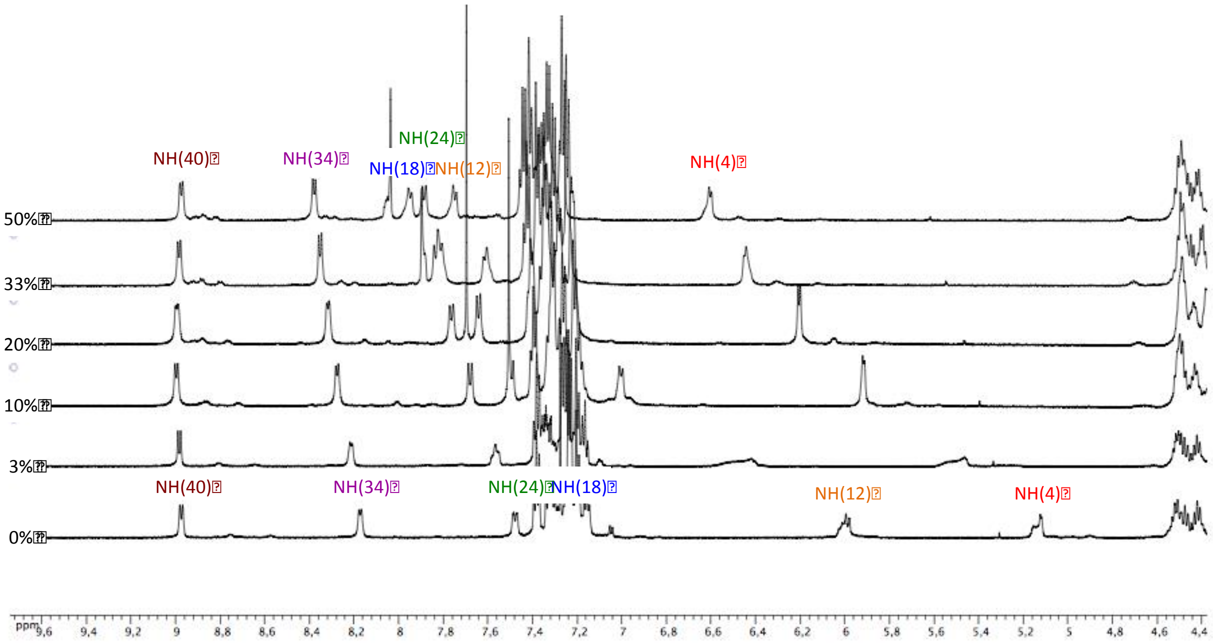

Boc–Phe–(*R*)-γ⁴-Ala–(1*S*,2*S*)-ACBC–(*R*)-γ⁴-Phe–(1*S*,2*S*)-ACBC–(*R*)-γ⁴-Leu–OMe 4

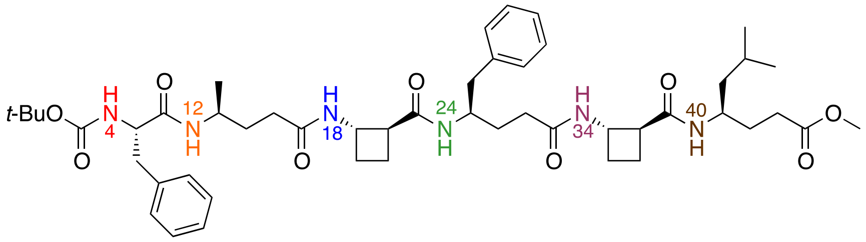

DMSO-d_6 (%, v/v)							
NH	**0%**	**3%**	**10%**	**20%**	**33%**	**50%**	**Δδ**
NH(4)	5.17	5.54	5.88	6.12	6.34	6.48	1.31
NH(12)	6.04	6.54	6.96	-	7.50	7.63	1.59
NH(18)	7.29	-	7.45	7.57	7.70	7.81	0.52
NH(24)	7.48	7.55	7.63	7.67	7.72	7.74	0.26
NH(34)	8.17	8.19	8.19	8.16	8.12	8.09	-0.08
NH(40)	8.23	8.22	8.21	8.22	8.22	8.21	-0.02

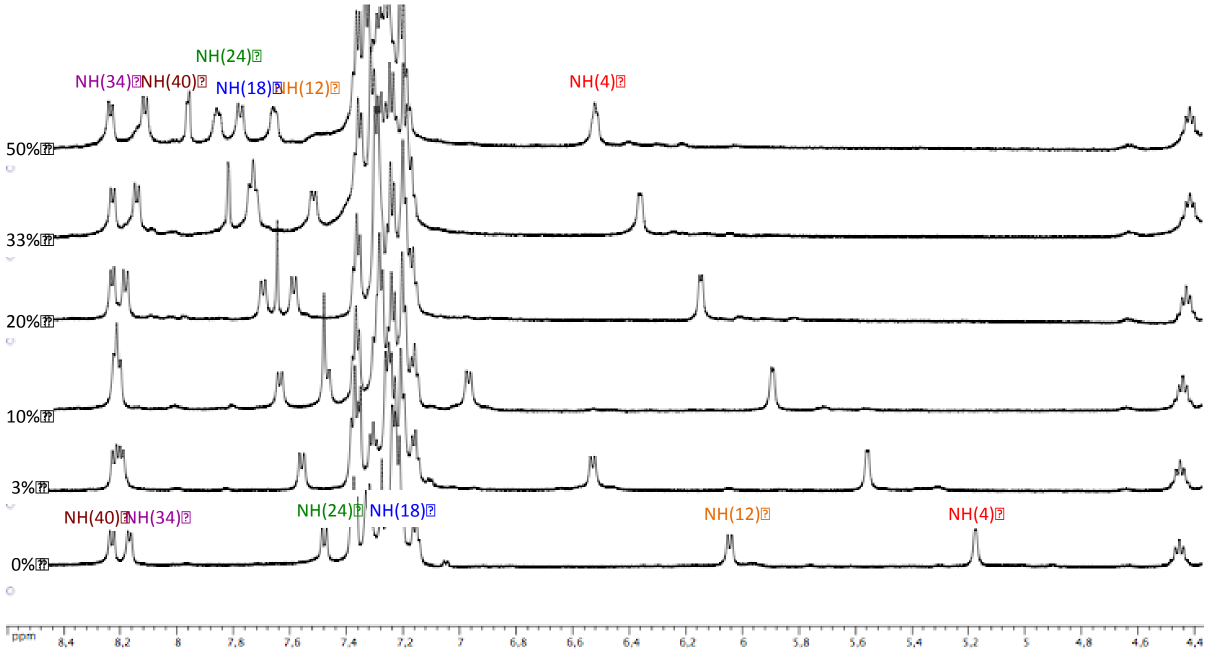

3. ROESY correlations

ROESY spectra were recorded at 300 K on a Bruker 600 MHz spectrometer. Samples were prepared in $CDCl_3$ at a concentration of 10 mM. The pulse sequence was roesyph. ROESY experiments employed a pulse spinlock of 200 ms. All experiments were performed by collecting 6492 points in f1 and 256 points 512 points in f2.

ROESY correlations representative of the 12-membered ring (C12):

Long-distance ROESY correlations representative of the 13-membered ring (C13):

Only long distance restraints are considered in this work. For a more extensive discussion of the ROSEY analyses of related β/γ peptides which includes analyses of both short and long range nOe correlations (the former of which may be consistent with alternative conformations).[3]

Boc–Phe–(*R*)-γ⁴-Ala–(1*S*,2*S*)-ACBC–(*R*)-γ⁴-Trp–(1*S*,2*S*)-ACBC–Leu–OMe 1

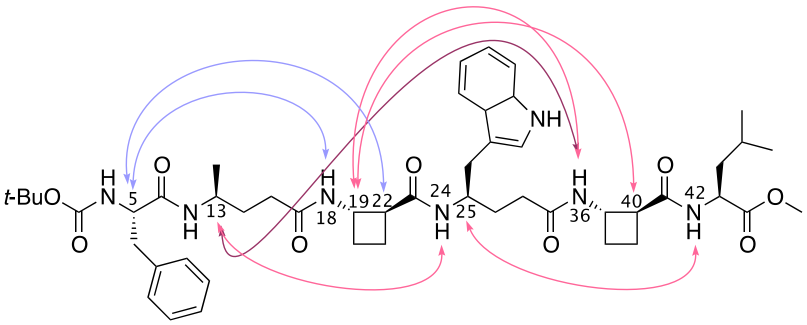

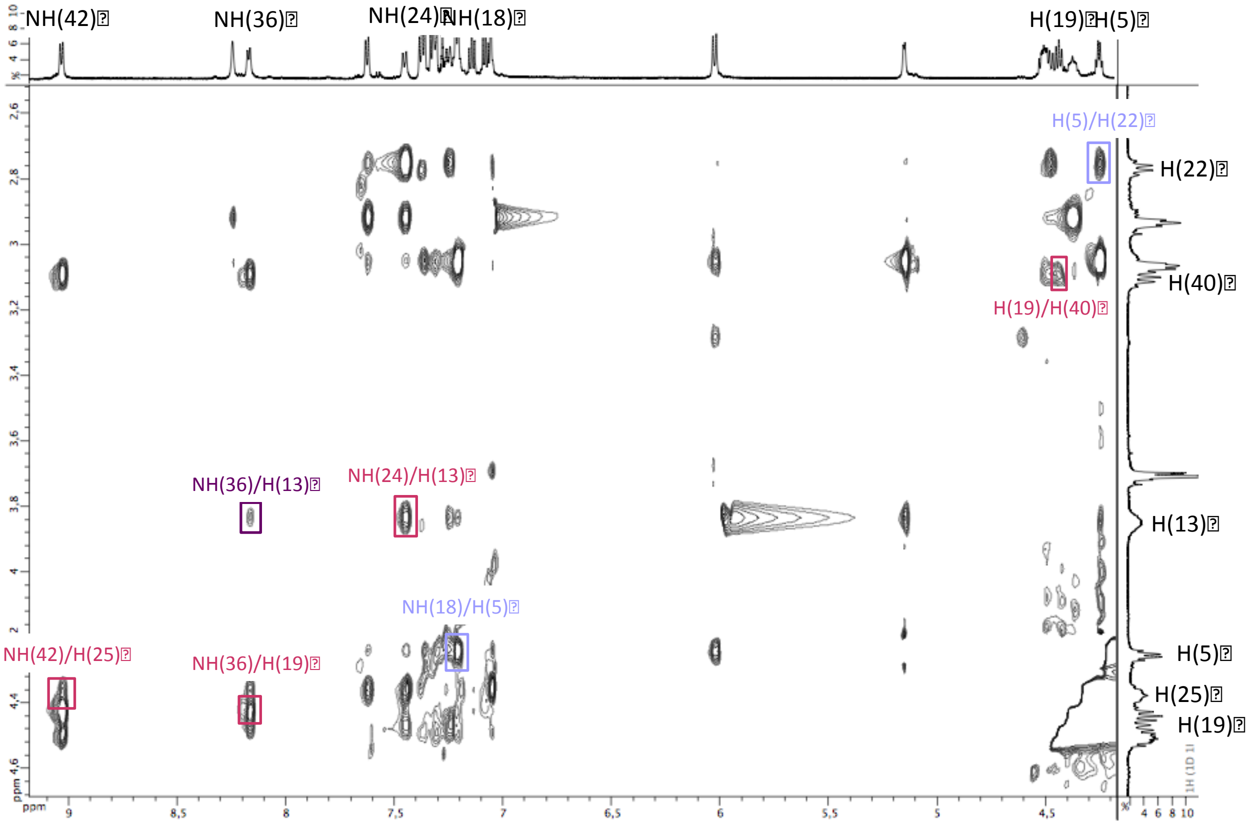

Boc–Phe–(*R*)-γ^4-Ala–(1*S*,2*S*)-ACBC–(*R*)-γ^4-Trp–(1*S*,2*S*)-ACBC–(*R*)-γ^4-Leu–OMe 2

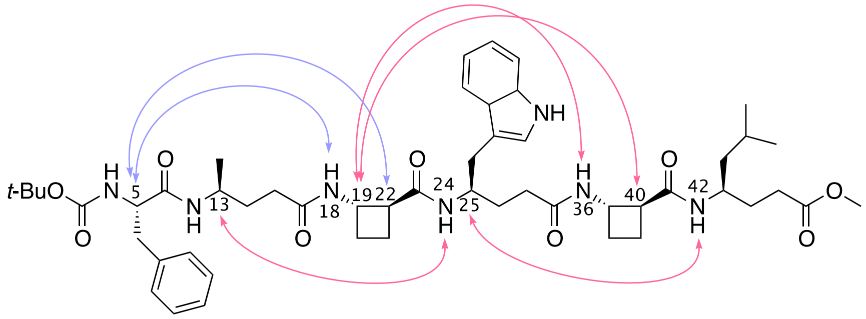

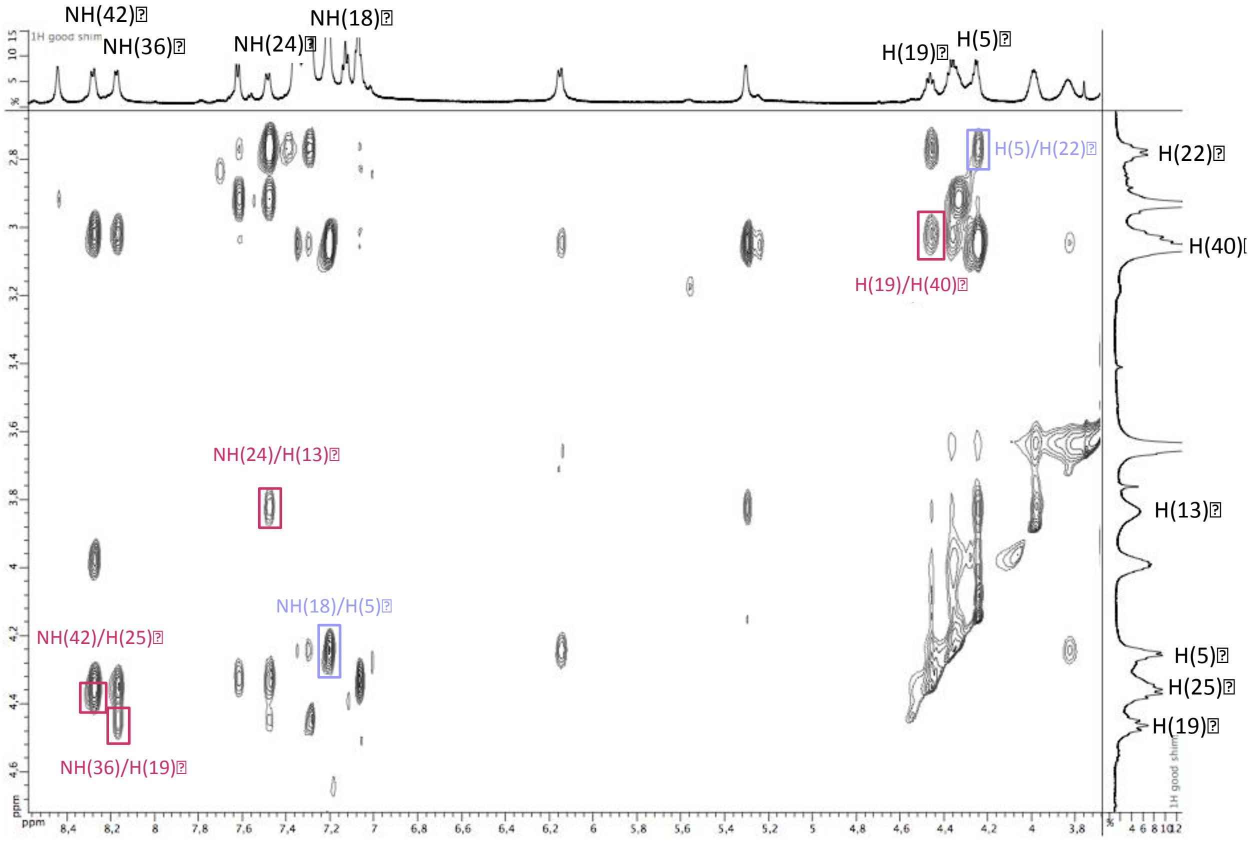

Boc–Phe–(*R*)-γ⁴-Ala–(1*S*,2*S*)-ACBC–(*R*)-γ⁴-Phe–(1*S*,2*S*)-ACBC–Leu–OMe 3

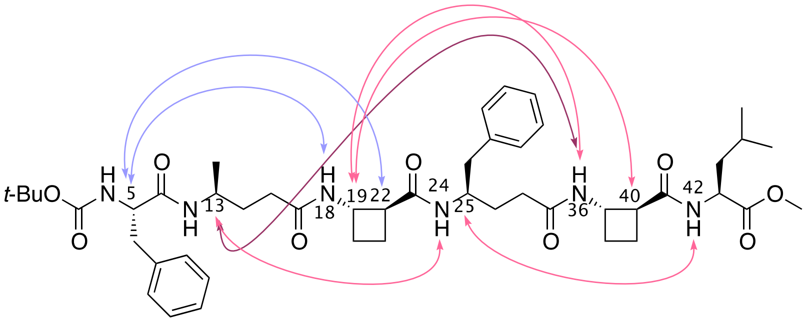

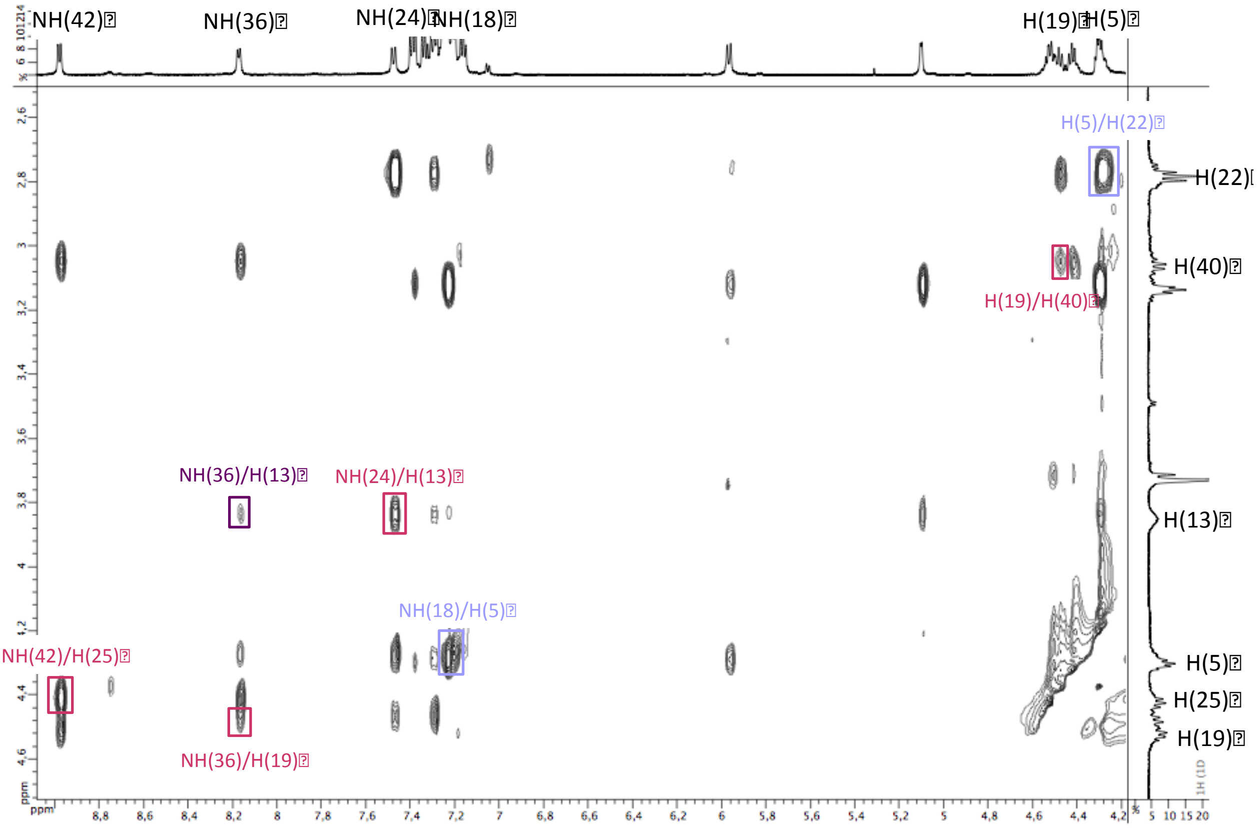

Boc–Phe–(*R*)-γ⁴-Ala–(1*S*,2*S*)-ACBC–(*R*)-γ⁴-Phe–(1*S*,2*S*)-ACBC–(*R*)-γ⁴-Leu–OMe 4

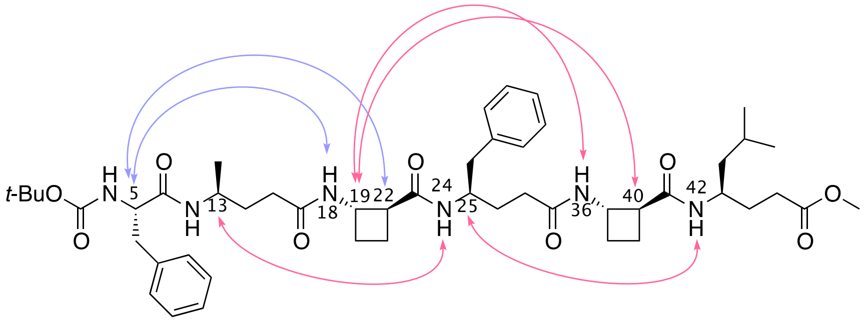

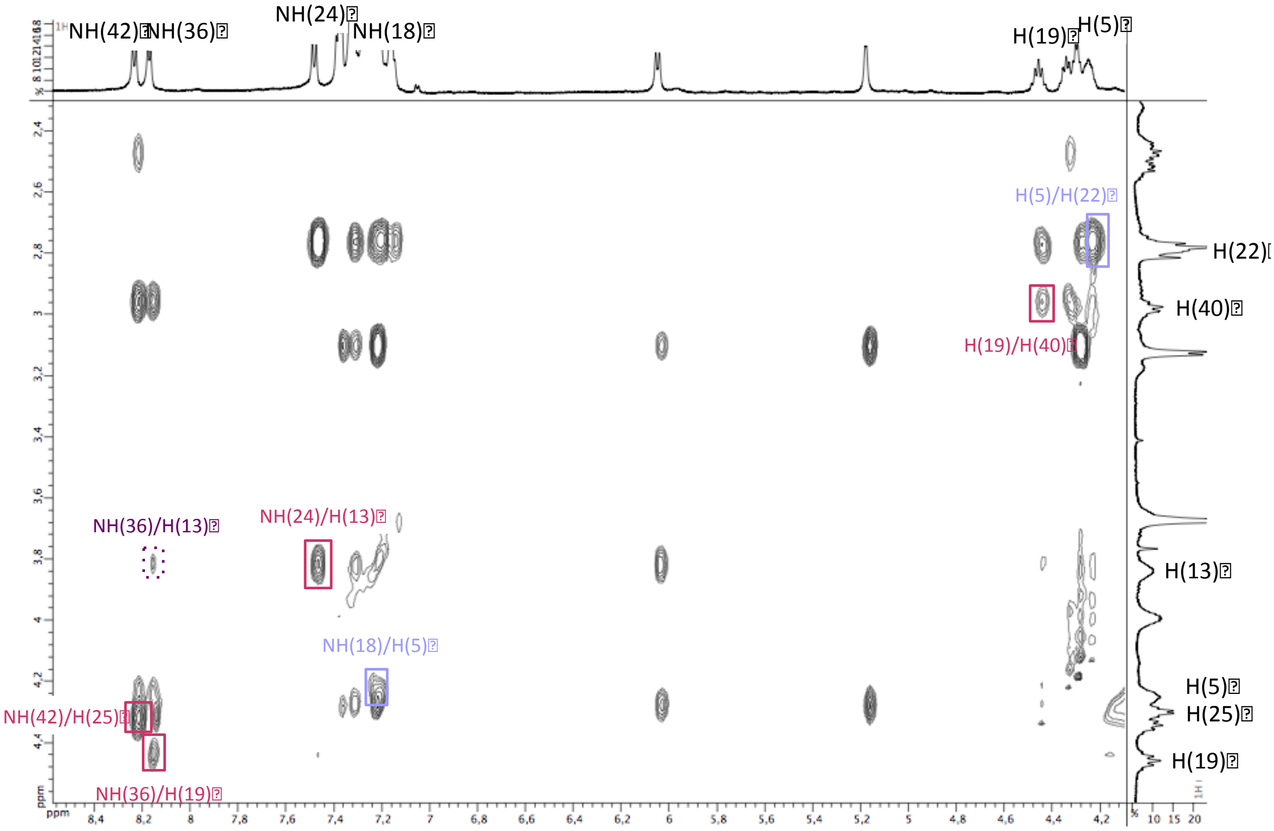

III. Molecular Modelling

1. *Hybrid MCMM calculation*

i. *Table of conformations obtained in $CHCl_3$, $C_8H_{17}OH$ and H_2O*

A hybrid Monte Carlo Molecular Mechanics (MCMM) conformational search was carried out on **1-4** in a chloroform and an octanol medium, on **5-8** in an octanol medium and on **1-8** in a water medium using Macromodel 04 from Schrödinger software and the MMFF force field without restraints. 10 000 conformers were generated by MCMM. Low energy conformers (up to 10 $kJ.mol^{-1}$ of relative energy) were retained. Different types of conformations were observed and were sorted following the hydrogen-bonded (H-bonded) ring systems they displayed:

- the 12,13-helix conformer contains only an N-terminal 12-membered hydrogen-bonded ring and successive 13-membered hydrogen-bonded rings.
- the other conformer are composed of different combinations of 8-, 13- and 15-membered H-bonded rings. Discreet, successive rings are separated by the symbol '-'.

The conformers are identified and their relative abundances indicated in the table below. The abundance of each conformer family is given as follows: (number of conformers of that family/total number of conformers; expressed as %) 12,13-Helix conformers are highlighted thus.

Conformations in $CHCl_3$	Abundance of each conformer family	Conformations in $C_8H_{17}OH$	Abundance of each conformer family	Conformations in H_2O	Abundance of each conformer family
Peptide **1** (67 conformers < 10 kJ.mol^{-1})		Peptide **1** (48 conformers < 10 kJ.mol^{-1})		Peptide **1** (90 conformers < 10 kJ.mol^{-1})	
12-13-13-13*	**100**	12-13-13-13*	**100**	12-13-13-13*	20
				/-13-13-13	**59**
				/-/-13-13	18
				8-/-13-13	3
Peptide **2** (25 conformers < 10 kJ.mol^{-1})		Peptide **2** (41 conformers < 10 kJ.mol^{-1})		Peptide **2** (110 conformers < 10 kJ.mol^{-1})	
12-13-13-13*	**100**	12-13-13-13*	**100**	12-13-13-13*	36
				/-13-13-13	**49**
				/-/-13-13	15
Peptide **3** (19 conformers < 10 kJ.mol^{-1})		Peptide **3** (21 conformers < 10 kJ.mol^{-1})		Peptide **3** (86 conformers < 10 kJ.mol^{-1})	
12-13-13-13*	**74**	12-13-13-13*	**100**	12-13-13-13*	20
12-13-13-8	26			/-13-13-13	27
				/-/-13-13	**46**
				8-/-13-13	5
				15-13-13	2
Peptide **4** (32 conformers < 10 kJ.mol^{-1})		Peptide **4** (13 conformers < 10 kJ.mol^{-1})		Peptide **4** (91 conformers < 10 kJ.mol^{-1})	
12-13-13-13	**67**	12-13-13-13*	**100**	12-13-13-13*	**34**
12-13-13-8	33			/-13-13-13	**34**
				/-/-13-13	28
				8-/-13-13	2
				/-9-13-13	2
		Peptide **5** (32 conformers < 10 kJ.mol^{-1})		Peptide **5** (97 conformers < 10 kJ.mol^{-1})	
		12-13-13-13*	**100**	12-13-13-13*	18
				/-13-13-13	**55**
				/-/-13-13	23
				8-/-13-13	4
		Peptide **6** (23 conformers < 10 kJ.mol^{-1})		Peptide **6** (100 conformers < 10 kJ.mol^{-1})	
		12-13-13-13*	**100**	12-13-13-13*	14
				/-13-13-13	**60**
				/-/-13-13	26
		Peptide **7** (42 conformers < 10 kJ.mol^{-1})		Peptide **7** (99 conformers < 10 kJ.mol^{-1})	
		12-13-13-13*	**100**	12-13-13-13*	14
				/-13-13-13	**47**
				/-/-13-13	30
				8-/-13-13	9
		Peptide **8** (50 conformers < 10 kJ.mol^{-1})		Peptide **8** (105 conformers < 10 kJ.mol^{-1})	
		12-13-13-13*	12-13-13-13*	12-13-13-13*	16
				/-13-13-13	**43**
				/-/-13-13	36
				8-/-13-13	5

ii. *Side and top views of the superimposed helical conformers (12-13-13-13 and /-13-13-13) of **1-8** in a water medium*

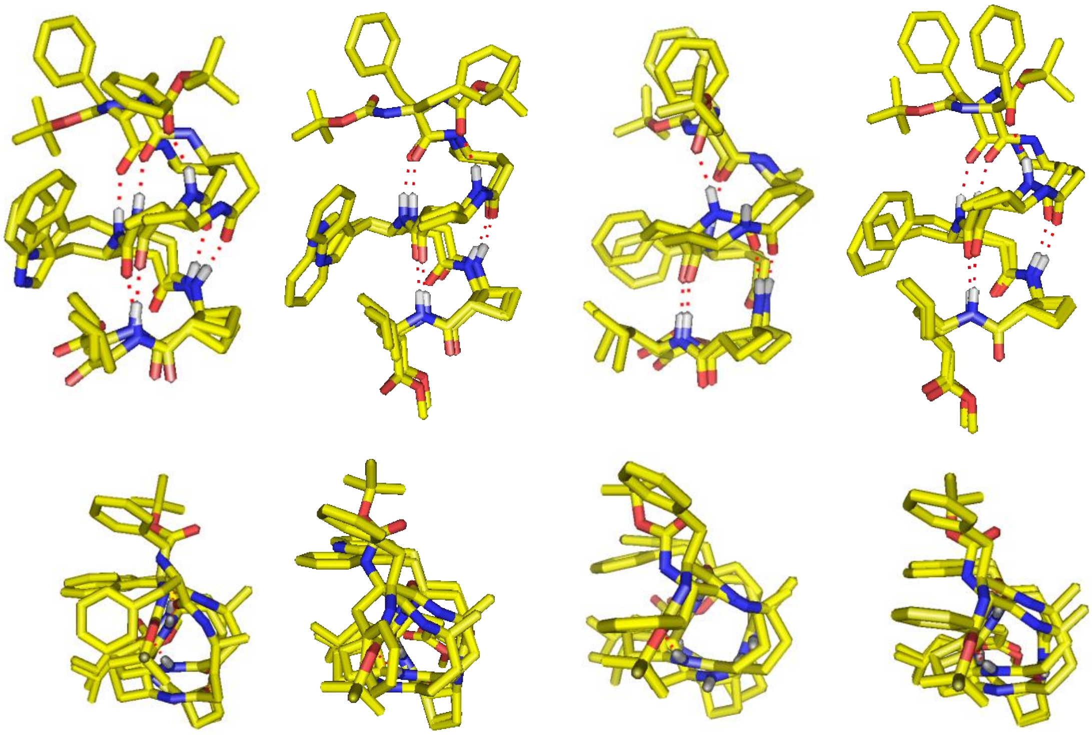

[1] [2] [3] [4]

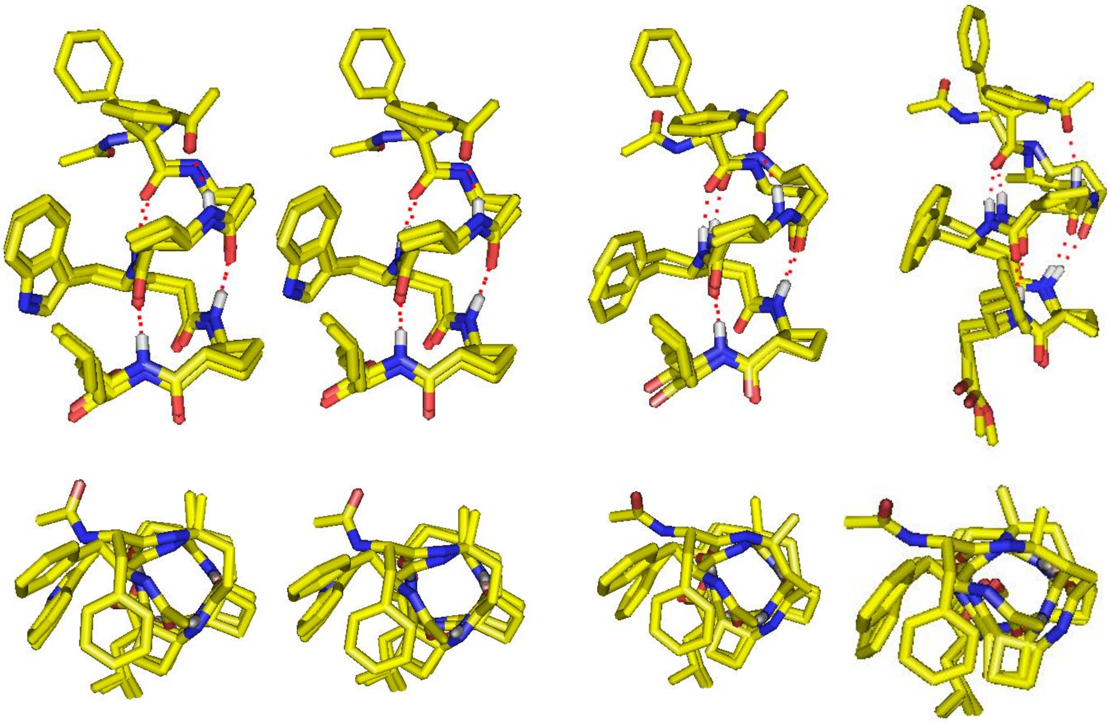

[5] [6] [7] [8]

2. Geometry optimization of the 12,13-helices by DFT

The geometries of the 12,13-helix conformers of peptides **1**-**8** obtained in the above MCMM search were each optimized by DFT using GAUSSIAN 09 and the B3LYP/6-311G(d,p) basis set in a chloroform medium.

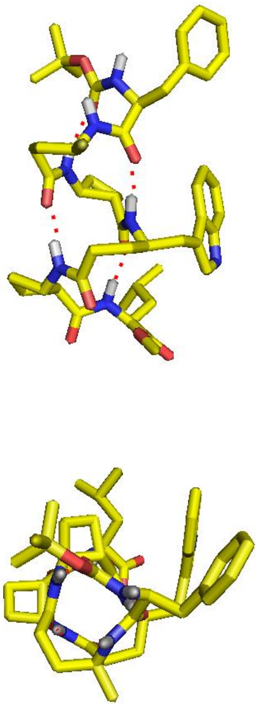

[1]

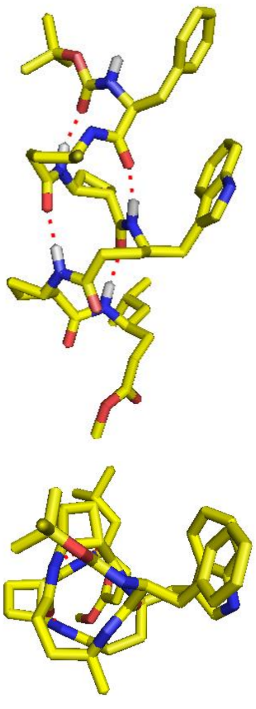

[2]

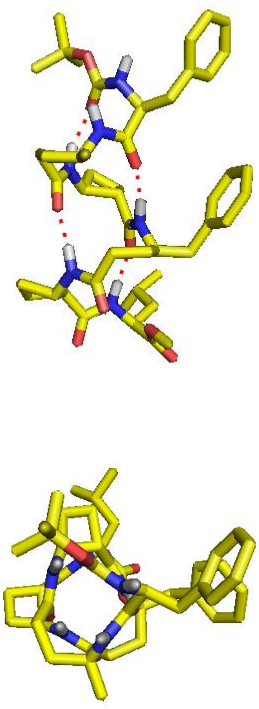

[3]

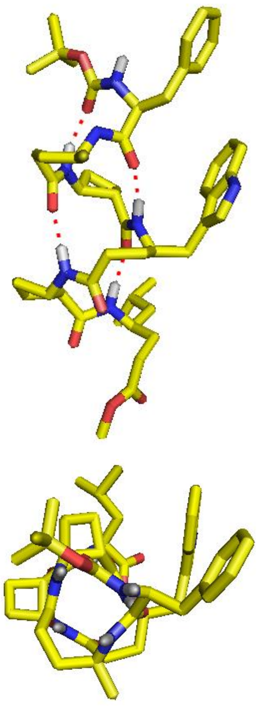

[4]

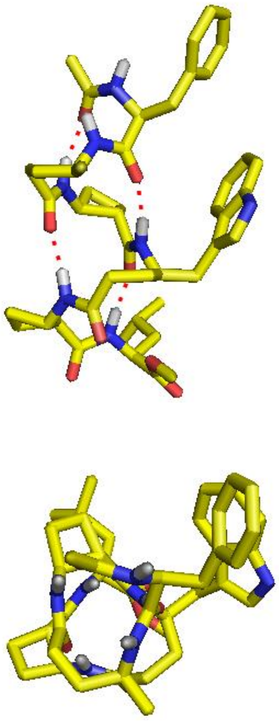

[5]

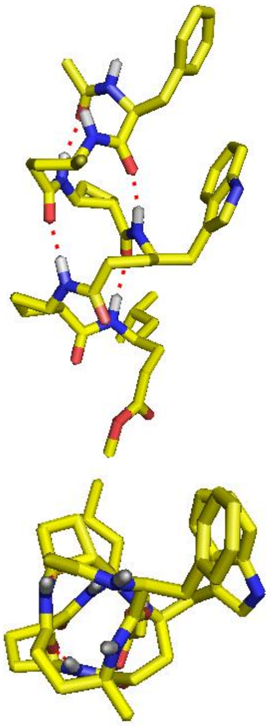

[6]

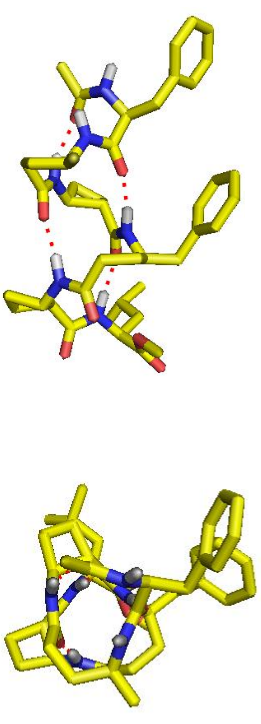

[7]

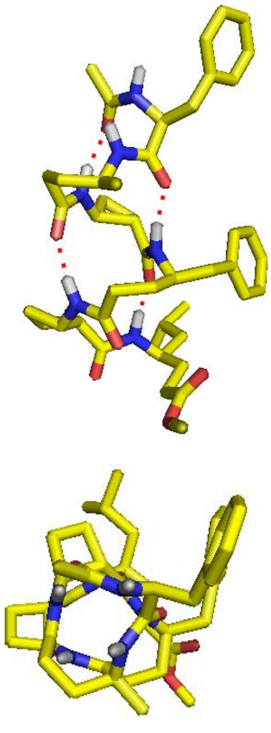

[8]

IV. Proteolysis studies of peptides 1-8

α-Chymotrypsin Type II from bovine pancreas (lyophilized powder, MW = 25 kDa, ≥ 40 units/mg protein) was purchased from Sigma-Aldrich and used without further purification.

α/β/γ-Peptides and p53(15-31) peptide (200 μM stock in PBS buffer pH 7.50, 2% DMSO) were treated with α-Chymotrypsin Type II from bovine pancreas (0.02 μM stock solutions in PBS buffer pH 7.50) in a 1:10000 enzyme/substrate ratio. The degradation was followed with analytical HPLC (Ascentis® Express Peptide Column, injection volume: 20 μL, acetonitrile/water (0.1% TFA) 5-95% gradient) and the data was analysed to extract kinetic values.

1. *HPLC traces of the positive control (native p53) in presence of α-Chymotrypsin*

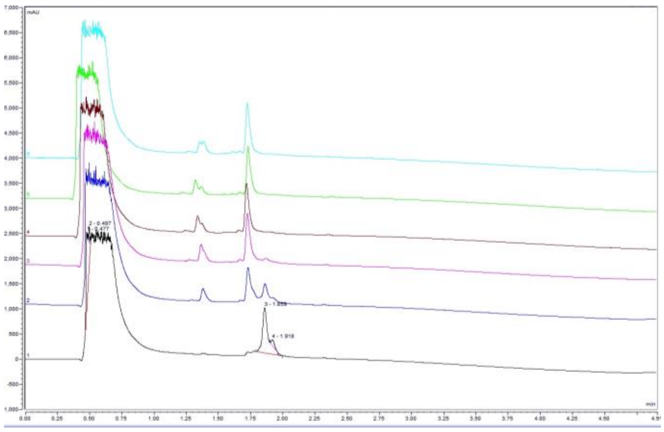

2. *HPLC traces of Boc-peptides **1-4** in presence of α-Chymotrypsin*

**1**	**2**
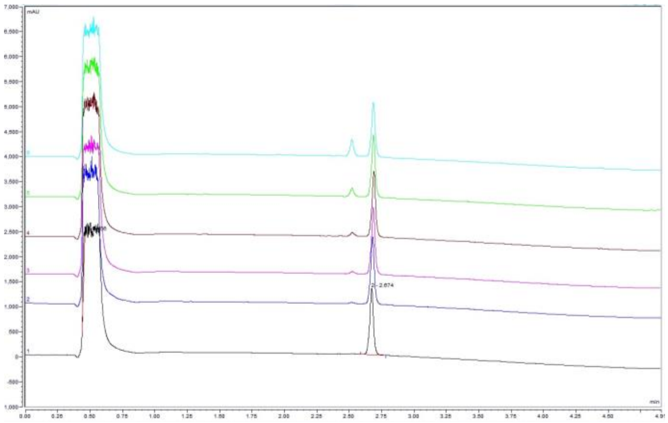	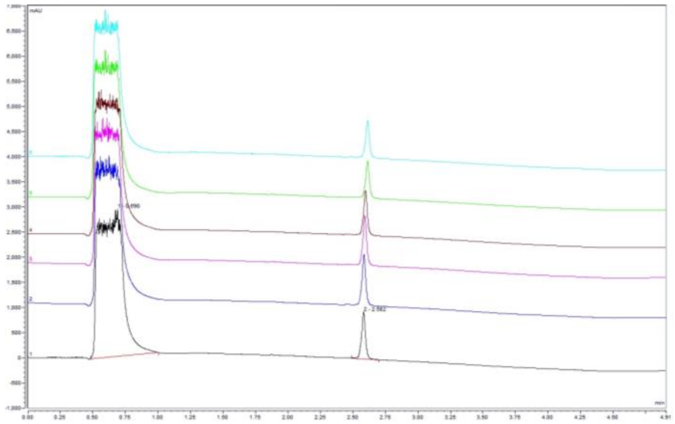
**3**	**4**
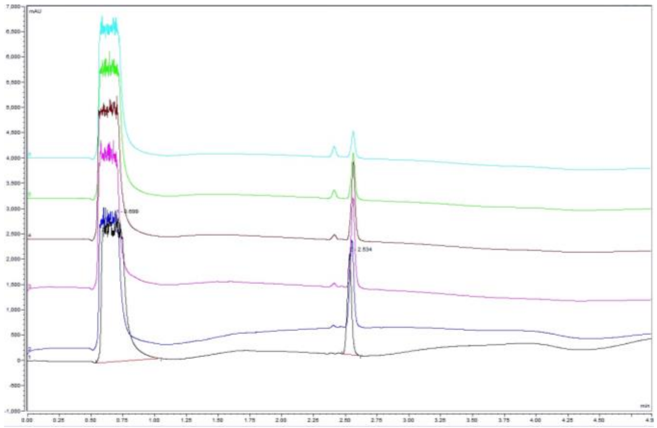	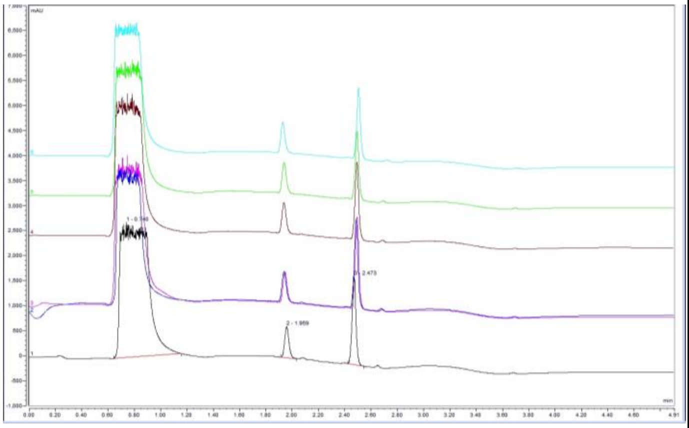

3. *HPLC traces of Boc-peptides **5-8** in presence of α-Chymotrypsin*

5

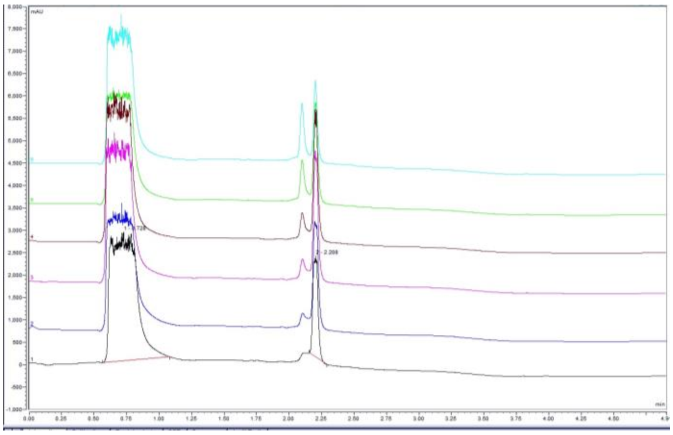

6

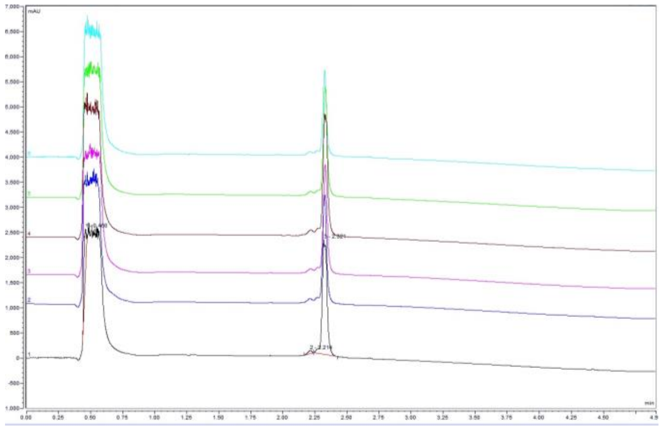

7

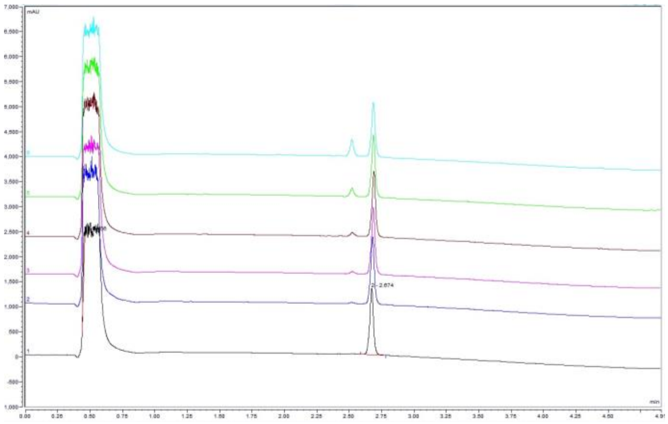

8

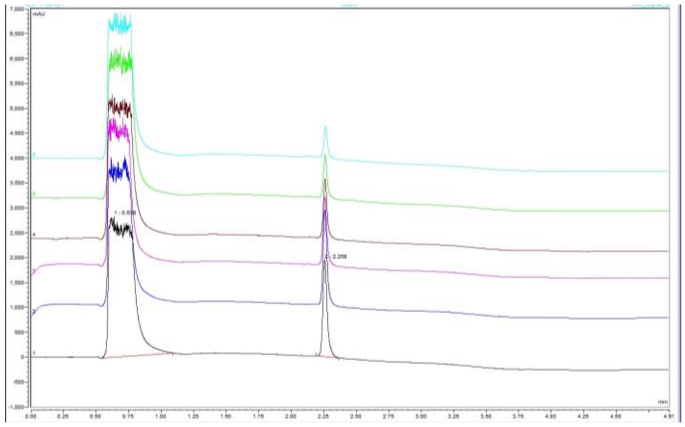

V. Fluorescence anisotropy competition assays with peptides 1-8

p53(15-31) transactivation domain peptide and its fluorescein-labelled analogue FITC-p53(15-31) were purchased from Peptide Protein Research Ltd and used without further purification. NOXA B(68-87) and its fluorescein-labelled analogue FITC-NOXA B(68-87) were synthesized and purified by Dr. Silvia Rodriguez Marin. Fluorescence anisotropy assays were performed in 384-well plates (Greiner Bio-one). Each experiment was run in triplicate and the fluorescence anisotropy measured using a Perkin Elmer EnVisionTM 2103 MultiLabel plate reader, with excitation at 480 nm (30 nM bandwidth), polarised dichroic mirror at 505 nm and emission at 535 nm (40 nM bandwidth, S and P polarised).

All experiments were performed in assay buffer: 40 mM phosphate buffer at pH 7.50, containing 200 mM NaCl and 0.02 mg mL-1 bovine serum albumin (BSA) and data analyzed following previously published methods.

20 μL of buffer were first added to each well. 40 μL of a solution of α-helix mimetics (1 mM in 90:10 (v/v) assay buffer: DMSO) were added to the first column. The solution was well mixed and 40 μL was taken out and added to the next column and so on. This operation consists on serial dilution of the peptides across the plate (starting point: 222 μM; 18-points, 3/4 serial dilution). For the p53/*h*DM2 FA competition assay, 20 μL of FITC-p53(15-31) Flu and 20 μL of *h*DM2(17-126) L33E were added to each well to give a final concentration of 50 nM and 150 nM, respectively. For the NOXA B/Mcl-1 FA competition assay, FITC-NOXA B(68-87) and Mcl-1(172-327) were added to each well to give a final concentration of 50 nM and 150 nM, respectively. For the Bak/Bcl-x_L FA competition assay, 20 μL of BODIPY-Bak and 20 μL of Bcl-x_L were added to each well to give a final concentration of 50 nM and 150 nM, respectively. For control wells, the tracer peptide was replaced with an identical volume of assay buffer. The total volume in each well was 60 μL. Plates were read after 1 h of incubation at room temperature.

The data for both the P (perpendicular intensity) and S (parallel (same) intensity) channels, resulting from this measurement and corrected by subtracting the corresponding control wells, were used to calculate the intensity and anisotropy for each well following Equations 1 and 2:

$$I = (2PG) + S \text{ (Equation 1)}$$

$$r = \frac{S - PG}{I} \text{ (Equation 2)}$$

Where I is the total intensity, G is an instrument factor which was set to 1 for all experiments and r is the anisotropy. The average anisotropy (across three experimental replicates) and the standard deviation of these values were then calculated and fit to a sigmoidal logistic model (Equation 3) using OriginPro 9.0 which provided the IC50 and error values.

$$y = r_{max} + \frac{r_{min} - r_{max}}{1 + \left(\frac{x}{x_0}\right)^p} \text{ (Equation 3)}$$

1. *Dose response curves of peptides **1-8** against BODIPY-BAK/Bcl-xL*

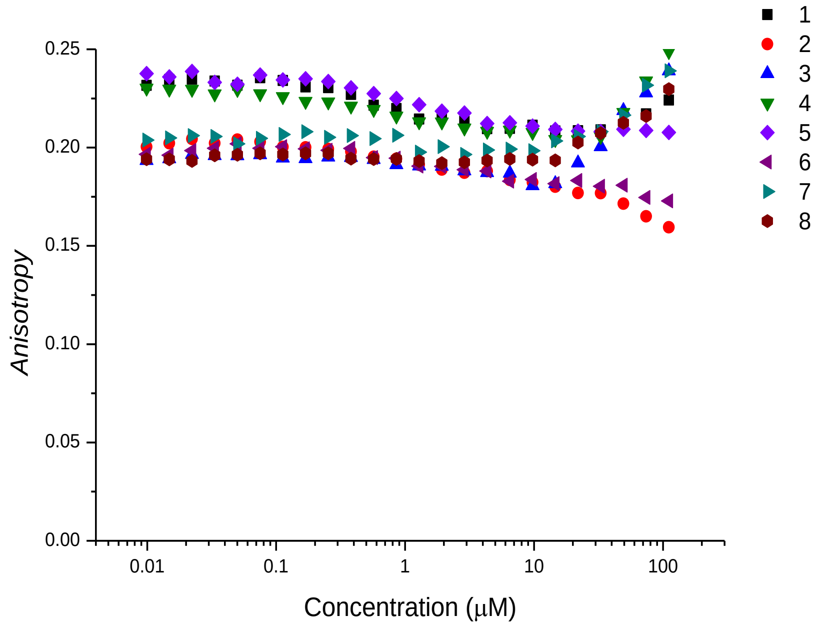

2. *Dose response curves of peptides **1-8** against FITC-NOXA B/Mcl-1*

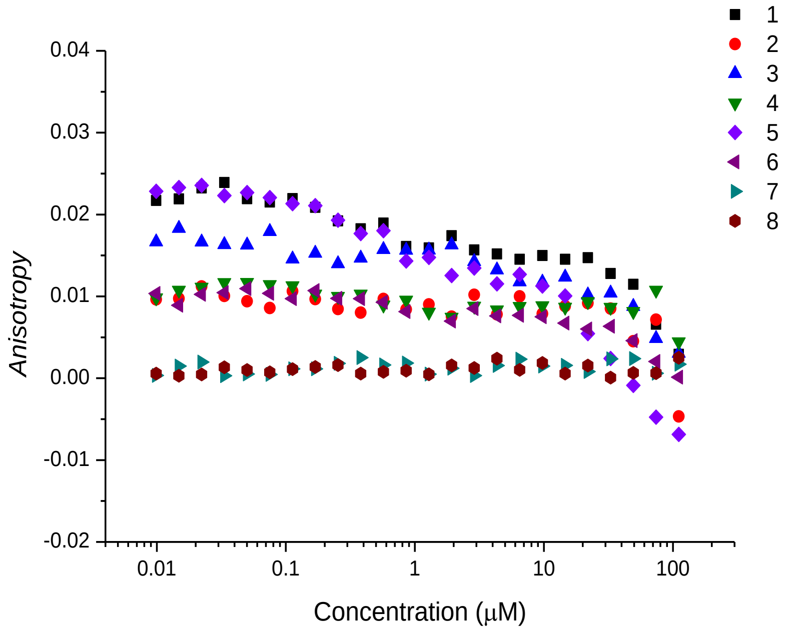

VI. ^{15}N-^{1}H HSQC NMR of peptides 2 and 8 in complex with *h*DM2

*h*DM2 (17-126) L33E was overexpressed in minimal media enriched with ^{15}N ammonium chloride to produce isotopically labelled proteins.

Each ^{1}H-^{15}N HSQC was obtained using 87 µM protein that had been incubated in the absence or presence of 200 µM compound overnight at 4°C. The buffer used for *h*DM2 spectra contained 100 mM sodium phosphate at pH 7.3, 2.5% of glycerol, 1 mM of DTT and 5% of DMSO. Sample volumes of 330 µL were placed in Shigemi BMS-005V tubes and included 10% D_2O v/v. All NMR datasets were acquired at 25 °C.

The crosspeaks were assigned from published structures, with BMRB entry 6621 used for *h*DM2.

The difference in chemical shift was calculated (Equation 4) where $\Delta\delta_{overall}$ is the overall change in chemical shift ΔδN is the change in the nitrogen dimension and ΔδH is the change in the hydrogen dimension. The change in hydrogen dimension is scaled by the ratio of the magnetogyric radius of nitrogen and hydrogen to account for the larger chemical shift range of nitrogen.

$$\Delta\delta_{overall} = \sqrt{(\Delta\delta_N)^2 + \left(\frac{\gamma_H}{\gamma_N}\right)^2 (\Delta\delta_H)^2} \quad \textbf{(Equation 4)}$$

*1. ^{15}N-^{1}H HSQC and changes in chemical shift of hDM2 in presence of peptide **8***

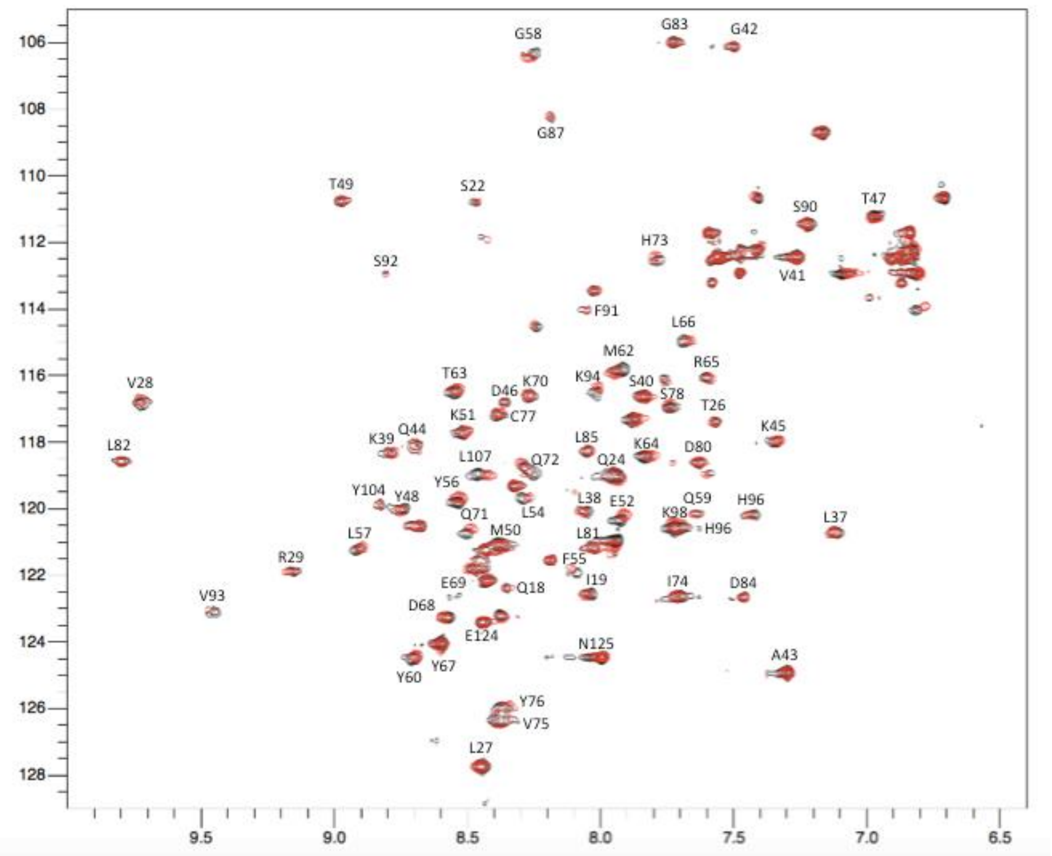

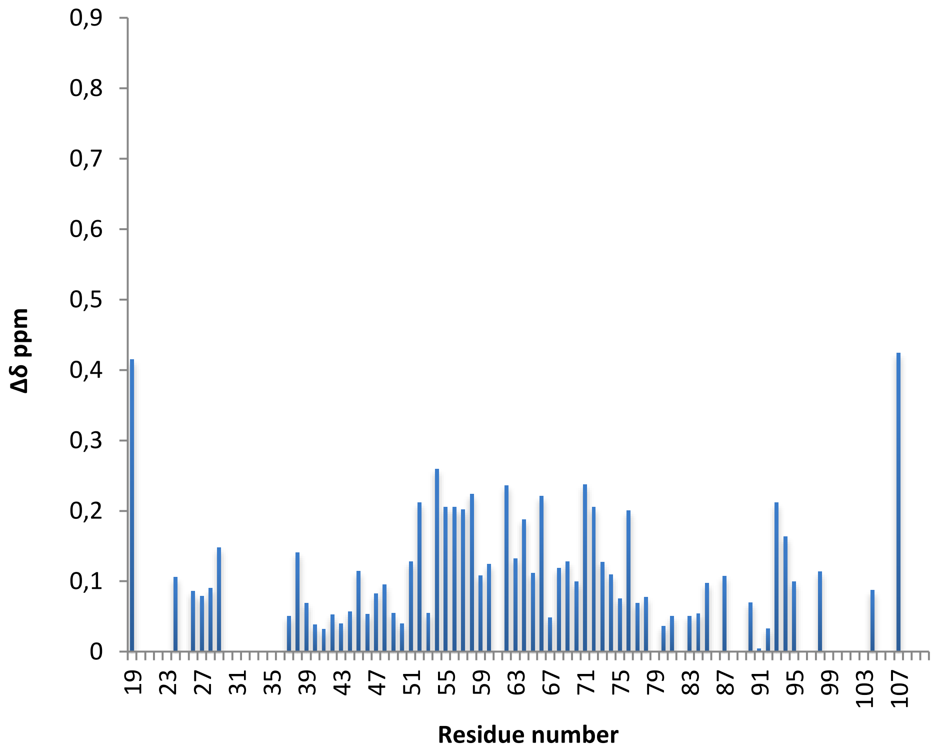

*2. ^{15}N-^{1}H HSQC and changes in chemical shift of hDM2 in presence of peptide **2***

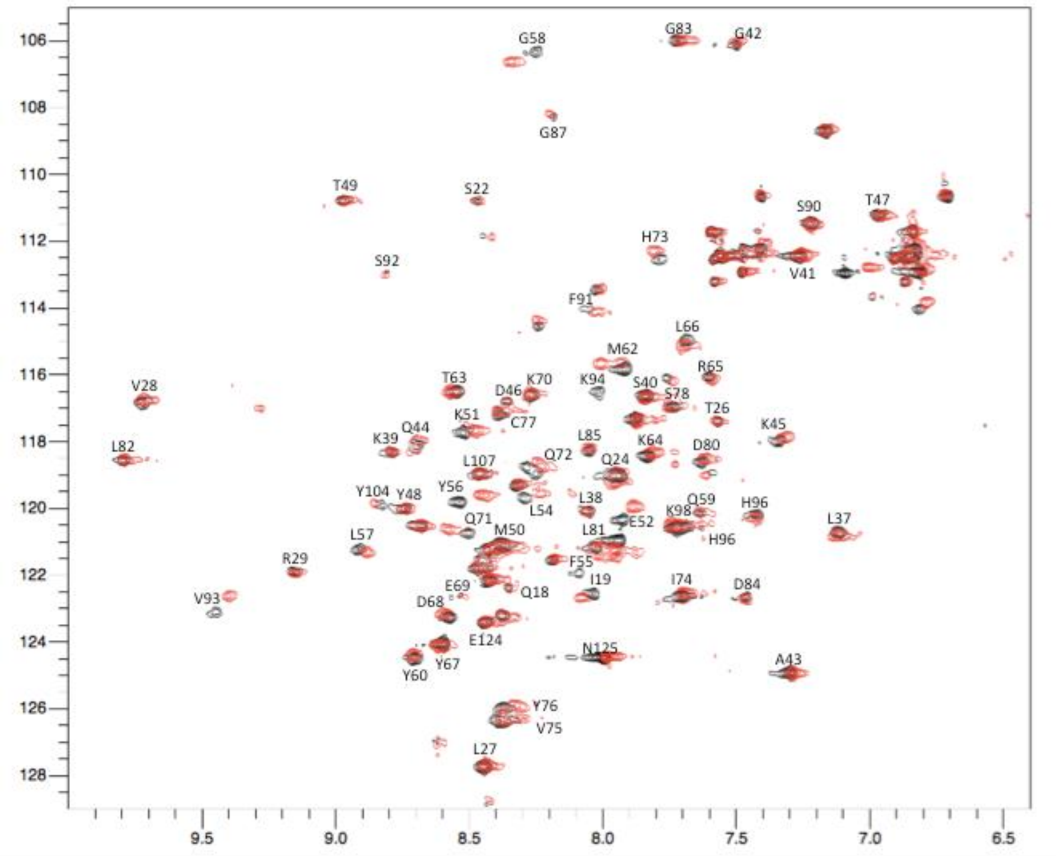

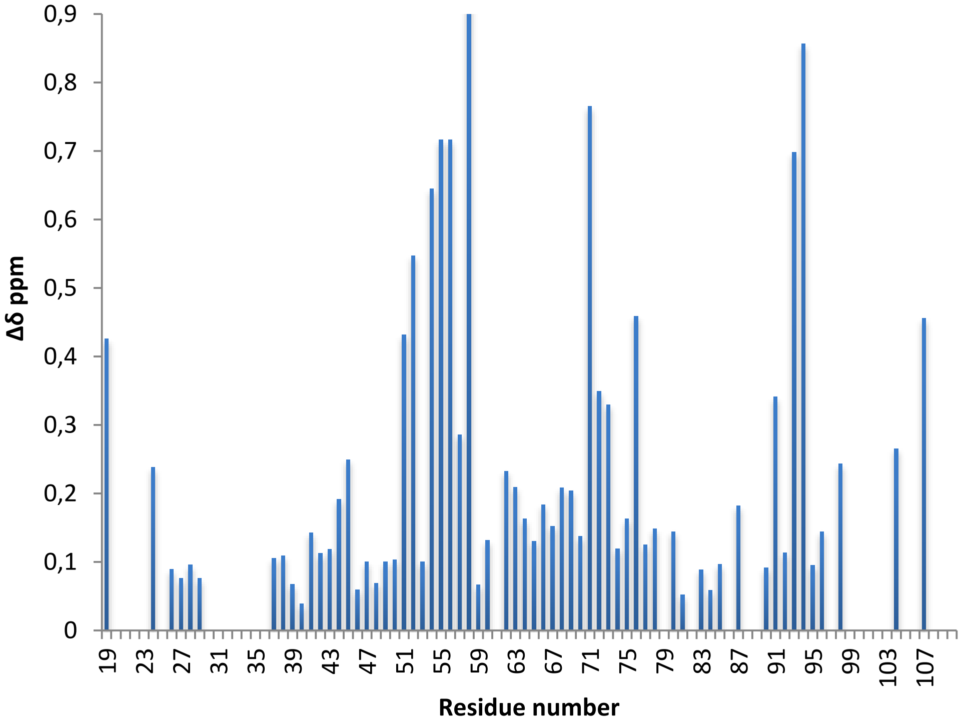

VII. References

[1] V. Declerck, D. J. Aitken, *Amino Acids* **2011**, *41*, 587.
[2] C. M. Grison, S. Robin and D. J. Aitken, Chem. Commun., **2015**, *51*, 16233.
[3] C. M. Grison, S. Robin, D. J. Aitken, *Chem. Commun.* **2016**, *52*, 7802.
